# Supplementary material for: Maternal socioeconomic status and infant feeding practices underlying pathways to child stunting in Cambodia: structural path analysis using cross-sectional population data
Source: BMJ Open. 2022 Nov 3;12(11):e055853. doi: 10.1136/bmjopen-2021-055853 (PMC9639063; doi:10.1136/bmjopen-2021-055853)
Supplement: Supplementary data [file bmjopen-2021-055853supp001.pdf]

Supplementary Table S1. Sample size selection: children aged 6 to <24 months with plausible anthropometric measurements, Cambodia (2014)

	n
Children 6-23.9 months	2,127
Children 6-23.9 months with anthropometric measurements	1,397
**Children 6-23.9 months with plausible† anthropometric measurements**	**1,365**

† Plausible HAZ scores should fall between -6 and +6 standard deviations, according to the WHO SD flag limits (WHO, 2006)

Supplementary Table S2. Distribution of dietary diversity tercile by child's age, adjusted estimates, Cambodia (2014)

	**Age in months (*n* = 1,381)**								
	**6-8 (*n*=242)**		**9-11 (*n*=208)**		**12-17 (*n*=463)**		**18-23 (*n*=468)**		**6-23 (*n*=1,381)**
**Dietary Diversity Tercile**	%	**DDS range**	**%**	**DDS range**	**%**	**DDS range**	**%**	**DDS range**	**%**
Low	44.9	0-1 groups	41.0	0-2 groups	49.5	0-3 groups	37.0	0-3 groups	**43.2**
Middle	17.9	2 groups	43.0	3-4 groups	26.2	4 groups	45.4	4-5 groups	**33.8**
High	37.2	≥ 3 groups	16.0	≥ 5 groups	24.4	≥ 5 groups	17.6	≥ 6 groups	**23.1**

Supplementary Table S3. Association between IYCF indicators, socio-demographic factors and mean height-for-age Z-scores in children aged 6-23 months in Cambodia (2014), (estimates adjusted for survey design using appropriate sample weights)

Indicator	%	N	Mean HAZ	Std. deviation	95% CI for mean	Test statistic
Inherent (biological) factors						
Age (months)						
6-8	17.5	242	-0.55	1.25	-0.75, -0.35	F(3, 1362)=22.20, *p*=<0.001
9-11	15.1	208	-0.92	1.48	-1.14, -0.70	
12-17	33.5	463	-1.41	1.23	-1.54, -1.28	
18-23	33.9	468	-1.46	1.32	-1.60, -1.31	
Sex						
Male	52.0	718	-1.28	1.34	-1.39, -1.16	F(1, 1364)=3.33, *p*=0.068
Female	48.0	663	-1.12	1.35	-1.24, -1.00	
Birth weight						
< 2.5kg	6.0	83	-1.48	1.50	-1.85, -1.11	F(3, 1362)=5.65, *p*=<0.001
2.5 < 4.0kg	83.4	1152	-1.16	1.33	-1.25, -1.07	
≥ 4.0kg	3.4	46	-0.82	1.10	-1.17, -0.46	
Missing/not weighed	7.2	100	-1.63	1.38	-1.91, -1.36	
Maternal Height						
Normal stature (≥145cm)	94.8	1308	-1.16	1.34	-1.25, -1.08	F(1, 1362)=20.26, *p*=<0.001
Short stature (<145cm)	5.2	71	-1.90	1.36	-2.21, -1.59	
Maternal BMI						
<18.5	15.6	215	-1.50	1.22	-1.68, -1.32	F(2, 1360)=5.72, *p*=0.003
18.5-24.99	69.2	952	-1.17	1.39	-1.27, -1.06	
≥ 25.0	15.2	209	-1.09	1.24	-1.31, -0.86	
Proximate factors						
Morbidity						
No	56.5	781	-1.16	1.40	-1.27, -1.05	F(1, 1364)=1.45, *p*=0.229
Yes	43.5	601	-1.26	1.28	-1.39, -1.13	
Breastfeeding						
No	29.9	413	-1.15	1.39	-1.30, -0.99	F(1, 1364)=0.66, *p*=0.4178
Yes	70.1	968	-1.22	1.33	-1.32, -1.12	
Bottle feeding						
No	66.0	911	-1.27	1.33	-1.38, -1.16	F(1, 1363)=5.39, *p*=0.020
Yes	34.0	469	-1.07	1.37	-1.20, -0.93	

**Indicator**	**%**	**N**	**Mean HAZ**	**Std. deviation**	**95% CI for mean**	**Test statistic**
Dietary Diversity Tercile						
Low	43.2	596	-1.30	1.20	-1.42, -1.17	F(2, 1363)=7.80, *p*=<0.001
Middle	33.8	467	-1.29	1.46	-1.44, -1.13	
High	23.1	318	-0.90	1.41	-1.07, -0.73	
Minimum meal frequency						
No	27.4	378	-1.32	1.38	-1.48, -1.16	F(1, 1364)=2.93, *p*=0.087
Yes	72.6	1003	-1.16	1.33	-1.25, -1.06	
Minimum acceptable diet						
No	68.4	945	-1.23	1.32	-1.33, -1.13	F(1, 1364)=1.13, *p*=0.287
Yes	31.6	436	-1.13	1.41	-1.29, -0.98	
Distal (underlying) factors						
Highest education level						
No education	12.0	166	-1.26	1.45	-1.50, -1.02	F(1, 1364)=6.90, *p*=0.009
Primary	55.6	768	-1.28	1.27	-1.40, -1.16	
Secondary/Higher	32.4	447	-1.04	1.42	-1.18, -0.91	
Labour force participation						
Not working	30.5	421	-1.19	1.22	-1.34, -1.04	F(2, 1359)=8.34, *p*=<0.001
Low	37.5	517	-1.40	1.42	-1.54, -1.26	
High	32.0	441	-0.98	1.34	-1.12, -0.84	
Household Wealth Index						
Poorest	25.4	345	-1.48	1.49	-1.65, -1.32	F(4, 1347)=7.53, *p*=<0.001
Poorer	18.2	248	-1.32	1.41	-1.50, -1.14	
Middle	19.9	271	-1.23	1.18	-1.41, -1.05	
Richer	17.7	241	-1.00	1.21	-1.20, -0.80	
Richest	18.8	256	-0.86	1.17	-1.04, -0.67	
Urban/rural residence						
Rural	86.4	1193	-1.25	1.23	-1.34, -1.16	F(1,1364)=13.04, *p*=<0.001
Urban	13.6	188	-0.88	1.97	-1.06, -0.70	
Sex of household head						
Male	77.0	1064	-1.20	1.33	-1.29, -1.10	F(1, 1364)=0.01, *p*=0.920
Female	23.0	317	-1.21	1.39	-1.40, -1.02	

Supplementary Table S4 Unstandardized and standardized results of the full structural path model examining the association between selected socioeconomic factors, mediating feeding practices and child HAZ, Cambodia (2014)

Path	Unstandardized (β)	Standardized (β)	R^2
Maternal employment (high)			0.15
Maternal education	0.167***	0.234***	
Household wealth			0.24
Maternal employment (high)	0.993***	0.327***	
Maternal education	0.706***	0.326***	
Dietary diversity			0.11
Maternal employment (high)	0.088	0.053	
Household wealth	0.097***	0.176***	
Breastfeeding	-0.191***	-0.111***	
Maternal education	0.081*	0.068*	
Breastfeeding			0.24
Maternal employment (high)	-0.071**	-0.073**	
Household wealth	-0.027**	-0.086**	
Maternal education	0.004	0.005	
HAZ			0.12
Maternal employment (high)	0.168	0.054	
Household wealth	0.058*	0.057*	
Dietary diversity	0.113*	0.061*	
Breastfeeding	-0.413***	-0.129***	
Maternal education	-0.019	-0.008	
**Overall R^2**			0.44
**χ^2 (df)**	65.70 (25)		
**RMSEA**	0.035		
**CFI**	0.972		

*p-value<0.05, **p-value<0.01, ***p-value<0.001

Supplementary Table S5 Proportion of women who reported breastfeeding at time of survey, by level of maternal participation in employment and child age group, Cambodia (2014)

	Age in months (*n*=1,365)				
	**6-8**	**9-11**	**12-17**	**18-23**	**6-23**
Not working or maternal employment (low)	95.3	93.6	85.7	42.3	75.5
Maternal employment (high)	82.6	78.8	72.0	34.0	59.0
X^2 test	X^2=8.52(1), *p*=0.004	X^2=7.06(1), *p*=0.008	X^2=7.52(1), *p*=0.006	X^2=2.03(1), *p*=0.155	X^2=24.72(1), *p*<0.001

Supplementary Table S6 Results of non-linear combinations for testing specific standardized indirect effects

**Path**	**Indirect Effect**			
	**Coef.**	**z**	**P-value**	**95% CI**
Maternal education -> Dietary diversity -> HAZ	0.004	1.63	0.104	(-0.00, 0.01)
Maternal education -> Breastfeeding -> HAZ	-0.001	-0.20	0.842	(-0.01, 0.01)
Maternal employment (high) -> Dietary diversity -> HAZ	0.003	1.41	0.159	(-0.00, 0.01)
Maternal employment (high) -> Breastfeeding -> HAZ	0.009	2.30	0.022	(0.00, 0.02)
Household wealth -> Dietary diversity -> HAZ	0.011	2.13	0.033	(0.00, 0.02)
Household wealth -> Breastfeeding -> HAZ	0.011	2.58	0.012	(0.00, 0.02)
