# Supplementary material for: Maternal socioeconomic status and infant feeding practices underlying pathways to child stunting in Cambodia: structural path analysis using cross-sectional population data
Source: BMJ Open. 2022 Nov 3;12(11):e055853. doi: 10.1136/bmjopen-2021-055853 (PMC9639063; doi:10.1136/bmjopen-2021-055853)
Supplement: Author's manuscript [file bmjopen-2021-055853.draft_revisions.pdf]

BMJ Open is committed to open peer review. As part of this commitment we make the peer review history of every article we publish publicly available.

When an article is published we post the peer reviewers' comments and the authors' responses online. We also post the versions of the paper that were used during peer review. These are the versions that the peer review comments apply to.

The versions of the paper that follow are the versions that were submitted during the peer review process. They are not the versions of record or the final published versions. They should not be cited or distributed as the published version of this manuscript.

BMJ Open is an open access journal and the full, final, typeset and author-corrected version of record of the manuscript is available on our site with no access controls, subscription charges or pay-per-view fees (http://bmjopen.bmj.com).

If you have any questions on BMJ Open's open peer review process please email info.bmjopen@bmj.com

BMJ Open

Maternal socioeconomic status and infant feeding practices underlying pathways to child stunting in Cambodia: structural path analysis using cross-sectional population data

Journal:	*BMJ Open*
Manuscript ID	bmjopen-2021-055853
Article Type:	Original research
Date Submitted by the Author:	08-Sep-2021

Complete List of Authors:	Harvey, Chloe; Independent Consultant<br>Newell, Marie-Louise; University of Southampton, Global Health Research Institute, School of Human Development and Health, Faculty of Medicine; University of the Witwatersrand, School of Public Health, Faculty of Health Science<br>Padmadas, sabu; University of Southampton, Social Statistics and Demography

Keywords:	Nutrition < TROPICAL MEDICINE, Community child health < PAEDIATRICS, PUBLIC HEALTH

SCHOLARONE™ Manuscripts

I, the Submitting Author has the right to grant and does grant on behalf of all authors of the Work (as defined in the below author licence), an exclusive licence and/or a non-exclusive licence for contributions from authors who are: i) UK Crown employees; ii) where BMJ has agreed a CC-BY licence shall apply, and/or iii) in accordance with the terms applicable for US Federal Government officers or employees acting as part of their official duties; on a worldwide, perpetual, irrevocable, royalty-free basis to BMJ Publishing Group Ltd ("BMJ") its licensees and where the relevant Journal is co-owned by BMJ to the co-owners of the Journal, to publish the Work in this journal and any other BMJ products and to exploit all rights, as set out in our licence.

The Submitting Author accepts and understands that any supply made under these terms is made by BMJ to the Submitting Author unless you are acting as an employee on behalf of your employer or a postgraduate student of an affiliated institution which is paying any applicable article publishing charge ("APC") for Open Access articles. Where the Submitting Author wishes to make the Work available on an Open Access basis (and intends to pay the relevant APC), the terms of reuse of such Open Access shall be governed by a Creative Commons licence – details of these licences and which Creative Commons licence will apply to this Work are set out in our licence referred to above.

Other than as permitted in any relevant BMJ Author's Self Archiving Policies, I confirm this Work has not been accepted for publication elsewhere, is not being considered for publication elsewhere and does not duplicate material already published. I confirm all authors consent to publication of this Work and authorise the granting of this licence.

For peer review only

Maternal socioeconomic status and infant feeding practices underlying pathways to child stunting in Cambodia: structural path analysis using cross-sectional population data

Chloe M. Harvey[1], Marie-Louise Newell[2,3] and Sabu S Padmadas[4]

[1]Independent Consultant, Bangkok, Thailand

[2]Global Health Research Institute, School of Human Development and Health, Faculty of Medicine, University of Southampton, Southampton, UK

[3]School of Public Health, Faculty of Health Science, University of the Witwatersrand, Johannesburg, South Africa

[4]Department of Social Statistics and Demography and Global Health Research Institute, University of Southampton, Southampton, UK

Corresponding author: Chloe M. Harvey, chloemercedes1991@gmail.com

Word count: 4563

For peer review only

ABSTRACT

Objectives: To identify and investigate complex pathways to stunting among children aged 6-24 months to determine the mediating effects of dietary diversity and continued breastfeeding on the association between socioeconomic factors and child stunting. Secondly, to assess whether these pathways are moderated by urban/rural residence.

Design, setting and participants: We analysed the most recent cross-sectional Demographic and Health Survey data from Cambodia (2014). We applied structural path analysis on a sample of 1,365 children, to model the complex and inter-related pathways of factors determining children's height-for-age. Explanatory variables included a composite indicator of maternal employment, household wealth, maternal education, current breastfeeding status and dietary diversity score. Results are presented both in terms of non-standardized and standardized coefficients. To examine the moderating effect of urban/rural residence on the proposed structural path models, both constrained and unconstrained solutions were tested.

Outcome measure: The primary outcome measure was height-for-age Z-scores (HAZ) as a continuous measure.

Results: Findings suggest that children's dietary diversity and continued breastfeeding mediate the association between socioeconomic status and children's height. Whilst there was no significant direct effect of maternal education on children's height, results suggested significant indirect pathways through which maternal education effects children's height; operating through household wealth, maternal employment, dietary diversity and continued breastfeeding status *(p*<0.001*)*. Most notably, 41% of the effect of maternal employment on children's height was mediated by either dietary diversity or continued breastfeeding. Furthermore, these associations differed between urban and rural areas, with results from simultaneous path analysis by urban-rural residence demonstrating that the strength of the associations between underlying socioeconomic factors and infant and young child feeding practices vary by urban-rural residence.

Conclusion: We provide evidence to support targeted nutrition interventions which account for the different ways in which poverty is experienced in urban and rural areas.

For peer review only - http://bmjopen.bmj.com/site/about/guidelines.xhtml

Article Summary

Strengths and limitations of this study

- Application of a causal hypothetical model to explain possible pathways to children's linear growth.
- Hypothetical models that can be applied to similar geographical contexts.
- The cross-sectional nature of DHS data, requires that results be interpreted with some caution.
- The theoretical model was built iteratively based on prior theory and thus, is not an exhaustive representation of the pathways to children's height.

For peer review only

For peer review only - http://bmjopen.bmj.com/site/about/guidelines.xhtml

INTRODUCTION

Across Southeast Asia, stunting in children under five years of age continues to pose a critical public health concern, with most countries in the region experiencing prevalence levels above 30%.[1] In Cambodia, more than a third of children under the age of five years are stunted;[1] stunting has been shown to be associated with distinct socioeconomic inequality, with sub-optimum feeding practices especially after 6 months of age, micronutrient deficiencies and recurrent infections.[2-3]

Impaired linear growth has adverse long-term health consequences for cognitive and immune system development, and is associated with increased risk of overweight and co-morbidities in later life.[2, 4, 5] Evidence shows that childhood stunting also has intergenerational implications, as stunting in pregnant women may lead to intrauterine growth restriction in her offspring,[6] thus continuing the vicious cycle of undernutrition. Interventions to tackle undernutrition in Southeast Asia have not been a top priority for governments and other stakeholders,[7] suggesting the need for strengthening further scientific evidence to understand the causal pathways of undernutrition, to identify appropriate strategies for policy and programme interventions.

Bio-behavioural and socioeconomic factors associated with children's height-for-age operate at varying levels of causation.[2] Limited dietary diversity has previously been reported to be significantly associated with impaired linear growth,[8] whereas the role of continued breastfeeding beyond six months remains inconclusive, with some studies even suggesting a negative association.[9, 10] However, it is not clear whether early feeding practices are directly and independently associated with height-for-age or whether they mediate the association between distal (underlying) factors and stunting.

Studies in Cambodia and other low- and middle-income countries have consistently shown maternal education, household wealth and maternal employment to be associated with inequalities in stunting prevalence.[2, 11-15] These three socioeconomic factors have also been associated with stark differentials in breastfeeding practices and dietary diversity within the Southeast Asian context.[16] However, associations underlying improved maternal socioeconomic status, continued breastfeeding and dietary diversity are paradoxical, as improvements in socioeconomic status (such as income, employment and education) are congruent with declines in breastfeeding, whereas improved socioeconomic status with increasing household income is positively associated with improved dietary diversity. Previous research has also indicated significant differentials in child nutritional status according to urban/rural residence,[2, 15] however, the intra-urban and intra-rural differentials are rarely considered, despite research suggesting starker socioeconomic inequalities within urban areas in low- and middle-income country settings.[17]

For peer review only - http://bmjopen.bmj.com/site/about/guidelines.xhtml

Most cross-sectional studies to date focusing on factors associated with linear growth have examined children's height-for-age as a dichotomous outcome, expressed as stunting. Evidence on the complex and inter-related nature of factors associated with a continuous measure of children's height-for-age is limited, including modelling techniques that account for dynamic interactions between possible explanatory variables, to disentangle direct effects from indirect effects with mediating variables.[18-20] To date, no such study has focussed on the Southeast Asian region, including Cambodia, where evidence-based interventions are critical for tackling the population burden of child undernutrition.

Using cross-sectional data from the most recent National Demographic and Health Survey (CDHS) in Cambodia, we identify and investigate theoretical pathways to stunting among children aged 6-24 months, when breastfeeding and dietary practices are most important for growth. We use structural path analysis to examine three hypotheses:(1) socioeconomic factors are directly associated with dietary diversity and continued breastfeeding;(2) dietary diversity and continued breastfeeding beyond six months of age mediate the association between socioeconomic factors and children's height-for-age, partially explaining the association between underlying socioeconomic factors and the outcome (children's height);(3) pathways are moderated by urban/rural residence (underlying socioeconomic factors have differing impacts on pathways to stunting, according to urban/rural residence).

METHODS

Data

We used the most recent Demographic and Health Survey (DHS) data from Cambodia,[21] which adopted a two-stage stratified sampling by separating rural and urban areas. The survey interviewed 17,578 women aged 15-49 years from 15,825 households, which included a sample of women from 5,667 urban and 11,911 rural areas. Anthropometric measurements, child health and health care data were collected for only those births that occurred five years preceding the survey. All eligible mothers were asked about the breastfeeding status of their children under 36 months at the time of survey visit; 24 hour-recall of additional liquids and solid foods was collected for the youngest child <36 months.

The sample of interest for this study was the youngest, living singleton child, between the ages of 6 and <24 months at the time of survey, living in the same household as their mother. As per CDHS

For peer review only - http://bmjopen.bmj.com/site/about/guidelines.xhtml

(2014) sampling design, anthropometric measurements were only taken in two-thirds of households, and after accounting for missing or implausible anthropometric information, a total of 1,365 Cambodian children between the ages of 6 and <24 months were selected (Supplementary Table S1). The children's file contains the records for every child born in the five years preceding the survey for women aged 15-49 years, who were selected randomly from the sampled households within each cluster.

The response rate for the women's survey from which information about the children is collected, was 98%. Additional information on the CHDS sampling design and survey methodology is reported elsewhere (CDHS, 2014). Item non-response for the selected explanatory variables was minimal, and the decision was made to exclude cases with missing data at random from the final analysis (<0.5% of total cases).

No additional data is available.

Dependent variable

Anthropometric data were collected by trained interviewers using internationally standard instruments. Height measurements were taken using a SECA measuring board.[21] For children aged less than 24 months, recumbent length measurements were taken and for older children, standing height was measured.[21]

Children's height-for-age Z-scores were calculated by the DHS using the WHO Child Growth Standards.[22] Stunting was defined as height-for-age Z-score below minus two standard deviations (-2 SD) from the mean of the reference population and severe stunting as below minus three standard deviations (-3 SD).[22] We used height-for-age Z-scores (HAZ) as a continuous measure, assuming that explanatory variables have varying effect on the respective Z-score distribution.

Explanatory variables

Selection of explanatory variables was based on previous research on the association between infant and young child feeding practices and child stunting, as well as two conceptual frameworks which depict the pathways through which different factors may affect child height/growth at different levels.[23, 24] Children's dietary diversity and breastfeeding at time of survey were selected as the two main proximate factors in the hypothetical structural path models. This was on the basis of evidence from the Cambodian DHS that suggests the main reasons for Cambodian children not meeting the minimum acceptable diet was due to not meeting the recommended minimum dietary diversity (consumption of four or more food groups in the previous 24 hours) but not due to meal

For peer review only - http://bmjopen.bmj.com/site/about/guidelines.xhtml

frequency.[21] Inclusion of continued breastfeeding status separately is important as while it is not included as a food group in the original WHO definition of minimum dietary diversity (MDD-7), it is recommended as an additional source of nutrients during the complementary feeding period.[25]

Terciles of dietary diversity were computed by age in months, based on the number of food groups consumed by children in the 24 hours preceding the survey,[26] to account for the gradual introduction of complementary foods.[27] Thus, children were regarded as having a low, middle or high diversity, relative to children of the same age. The definition of the terciles vary by age; Supplementary table S2 presents the distributions of the dietary diversity terciles by age.

Breastfeeding was categorised as a dichotomous variable as whether the child was being currently breastfed or not at child's age or time of survey.

The distal (underlying) factors included maternal education, household wealth and maternal employment. Maternal education reflected the highest level of completed education and was categorised into no education, primary, secondary and higher. Quintiles of household wealth were computed separately for urban and rural areas by DHS using principal component analysis, to ensure the representation of the poorest of the poor households, especially in rural areas.[28]

As children's nutritional status and feeding practices may be determined by a combination of different aspects and types of maternal employment,[29, 30] we created a composite indicator measuring maternal participation in the labour force based on an indicator computer by Sebayang, Efendi and Astutik.[31] Using principal component analysis, we incorporated five aspects of maternal employment: 1) working status over the past 12 months; 2) type of employment, whether self-employed or employed by family; 3) type of occupation; 4) type of earnings and 5) seasonality of work. Three distinctive categories of maternal employment were identified: not working, low level of participation, high level of participation, which was further dichotomised into a binary variable: (0) Not working/low participation; (1) High participation.

Statistical Analysis

Bivariate analyses included adjusted Wald tests for joint hypothesis testing to assess preliminary associations between explanatory variables and height-for-age Z-scores (Supplementary table S3). Statistical significance was considered at $p<0.05$ level.

Analysis was conducted using Stata/SE 15.1.[32] All descriptive and exploratory analyses accounted for the complex survey design of the CDHS using the appropriate sample weights.[21]

For peer review only - http://bmjopen.bmj.com/site/about/guidelines.xhtml

Structural path analysis is the structural component of structural equation modelling and involves the modelling of observed variables only.[33] Path analysis facilitates the study of direct and indirect effects of multiple independent and dependent variables simultaneously, based on a generalized multi-equation framework for examining multiple hypotheses about interdependencies between these variables.[34, 35] The first step of analysis involved creating a hypothetical "causal" pathway diagram between the independent variables of interest and the outcome HAZ (Figure 1). The hypothesised model was built iteratively based on prior theory and research, and therefore not an exhaustive representation of the pathways to children's height-for-age. It was selected as the appropriate methodology for this analysis as it allows for the identification of indirect and direct mechanisms through which socioeconomic factors influence height-for-age, where dietary diversity and breastfeeding may act as mediators in these associations.

Structural equation modelling assumes linearity of observed variables, and the assumption of linear normality amongst pseudo-continuous variables was tested with likelihood ratio (LR) tests and BIC/AIC diagnostics.[33] Most independent variables showed evidence of linearity, justifying their inclusion in the model as continuous variables. However, this was not the case for maternal employment as a continuous independent variable, and we thus used the dummy variable to adjust for high level of participation in employment, as this was the category of interest for this study.

To examine the moderating effect of urban/rural residence on the proposed structural path models, both constrained and unconstrained solutions were tested. The unconstrained solution where all parameters were allowed to vary by urban/rural residence provided a better fitting model and the results of this model are presented in this paper.

[Figure 1 Conceptual model of pathways between selected socioeconomic factors, dietary diversity, breastfeeding and height-for-age Z-scores (HAZ)]

Assessment of model fit

Structural path analysis was undertaken using the SEM program in Stata/SE 15.1. All models were estimated using maximum likelihood with missing values (MLMV). Goodness of fit of the data to the models were assessed using the root-mean-square error of approximation (RMSEA < 0.06) and the comparative fit index (CFI $\geq$ 0.95 for acceptance).[36] Both non-standardized and standardized coefficients are presented, but only standardized results are interpreted and presented on path diagrams (non-standardized coefficients use the raw/real life units of each variable and standardized

For peer review only - http://bmjopen.bmj.com/site/about/guidelines.xhtml

coefficients convert the coefficients into comparable units). Standardized coefficients are interpreted in terms of standard deviation differences.[34] To address the second hypothesis of this study that suggests dietary diversity and breastfeeding are mediators in the association between socioeconomic status and children's height-for-age Z-scores, standardized indirect effects were calculated.[33]

Finally, to test the moderating effect of urban-rural residence on path coefficients, as per the third hypothesis of this study, two approaches were taken. This part of the analysis involved comparing whether the associations presented in the hypothesised path model, varied by urban-rural residence and whether there were statistical differences for specific paths between urban-rural areas. Stata's SEM function facilitates the simultaneous analysis of the path model for different groups, to air comparison. The first approach involved an unconstrained model, where all parameters were permitted to vary by urban-rural residence and the second method involved a constrained solution in which all but one pathway was constrained to be the same. Results from both the unconstrained and constrained solution are presented.

Patient and public involvement

Secondary data was used for this analysis. Therefore, no patient consent was needed.

RESULTS

Sample characteristics

Approximately one-third of children were aged between 6-12 months and two-thirds were aged 12-23 months (Table 1), with 52% boys and 48% girls; 6% of children were born with a low birth weight (< 2.5kg). Just over two-fifth (44%) of children were reported to have had morbidity, either a fever, respiratory condition or diarrhoea, in the two weeks prior to interview. Five percent of mothers were of short stature (< 145cm); 16% had low (<18.5) and 15% high (≥25.0) BMI. Most mothers had completed primary level education (56%), and only 12% reported no formal education.
Approximately a third of women reported that they had not worked in the year prior to survey, a third of women had low level and the remainder high level of participation in employment. One in four of the children were from the poorest households and most households were located in rural areas (86%). About 70% of children aged 6-23 months were still being breastfed at the time of survey; 34% had been fed from a bottle. Approximately 43% of children had a low dietary diversity, but 73% of children consumed the recommended meal frequency.

For peer review only - http://bmjopen.bmj.com/site/about/guidelines.xhtml

Table 1 Summary characteristics of children 6-23 months from the Cambodian DHS (2014) (n=1,381*)

Indicator		%	N	Mean HAZ	Std. deviation	95% CI for mean	
						Lower bound	Upper bound
CHILD CHARACTERISTICS							
Age (months)	6-8	17.5	242	-0.55	1.25	-0.75	-0.35
	9-11	15.1	208	-0.92	1.48	-1.14	-0.7
	12-17	33.5	463	-1.41	1.23	-1.54	-1.28
	18-23	33.9	468	-1.46	1.32	-1.6	-1.31
Gender	Male	52.0	718	-1.28	1.34	-1.39	-1.16
	Female	48.0	663	-1.12	1.35	-1.24	-1
Birth weight	< 2.5kg	6.0	83	-1.48	1.5	-1.85	-1.11
	2.5 < 4.0kg	83.4	1152	-1.16	1.33	-1.25	-1.07
	≥ 4.0kg	3.4	46	-0.82	1.1	-1.17	-0.46
	Missing/not weighed	7.2	100	-1.63	1.38	-1.91	-1.36
Morbidity	No	56.5	781	-1.16	1.4	-1.27	-1.05
	Yes	43.5	601	-1.26	1.28	-1.39	-1.13
MATERNAL CHARACTERISTICS							
Height	Normal stature (≥145cm)	94.8	1308	-1.16	1.34	-1.25	-1.08
	Short stature (<145cm)	5.2	71	-1.9	1.36	-2.21	-1.59
BMI	<18.5	15.6	215	-1.5	1.22	-1.68	-1.32
	18.5-24.99	69.2	952	-1.17	1.39	-1.27	-1.06
	≥ 25.0	15.2	209	-1.09	1.24	-1.31	-0.86
Highest education level	No education	12.0	166	-1.26	1.45	-1.5	-1.02
	Primary	55.6	768	-1.28	1.27	-1.4	-1.16
	Secondary/Higher	32.4	447	-1.04	1.42	-1.18	-0.91
Maternal employment	Not working	30.5	421	-1.19	1.22	-1.34	-1.04
	Low	37.5	517	-1.4	1.42	-1.54	-1.26
	High	32.0	441	-0.98	1.34	-1.12	-0.84
HOUSEHOLD CHARACTERISTICS							
Household Wealth Index	Poorest	25.4	345	-1.48	1.49	-1.65	-1.32
	Poorer	18.2	248	-1.32	1.41	-1.5	-1.14
	Middle	19.9	271	-1.23	1.18	-1.41	-1.05
	Richer	17.7	241	-1	1.21	-1.2	-0.8
	Richest	18.8	256	-0.86	1.17	-1.04	-0.67
Urban/rural residence	Rural	86.4	1193	-1.25	1.23	-1.34	-1.16
	Urban	13.6	188	-0.88	1.97	-1.06	-0.7

For peer review only

For peer review only - http://bmjopen.bmj.com/site/about/guidelines.xhtml

Table 1 contd. Summary characteristics of children 6-23 months from the Cambodian DHS (2014) (n=1,381*)

Indicator		%	N	Mean HAZ	Std. deviation	95% CI for mean	
						Lower bound	Upper bound
Sex of household head	Male	77.0	1064	-1.2	1.33	-1.29	-1.1
	Female	23.0	317	-1.21	1.39	-1.4	-1.02
IYCF CHARACTERISTICS							
Breastfeeding	No	29.9	413	-1.15	1.39	-1.3	-0.99
	Yes	70.1	968	-1.22	1.33	-1.32	-1.12
Bottle feeding	No	66.0	911	-1.27	1.33	-1.38	-1.16
	Yes	34.0	469	-1.07	1.37	-1.2	-0.93
Dietary Diversity Tercile	Low	43.2	596	-1.3	1.2	-1.42	-1.17
	Middle	33.8	467	-1.29	1.46	-1.44	-1.13
	High	23.1	318	-0.9	1.41	-1.07	-0.73
Minimum meal frequency	No	27.4	378	-1.32	1.38	-1.48	-1.16
	Yes	72.6	1003	-1.16	1.33	-1.25	-1.06
Minimum acceptable diet	No	68.4	945	-1.23	1.32	-1.33	-1.13
	Yes	31.6	436	-1.13	1.41	-1.29	-0.98

* Sample size differs slightly from the sample used in the final models (*n*=1,365) due to missing data for some children on explanatory variables.

Results of structural path analysis

Underlying socioeconomic factors

Error! Reference source not found. Figure 2 represents the full structural path model; the standardized and unstandardized coefficients are presented in Supplementary Table S4. Overall, the fit of the model according to RMSEA (< 0.05) and CFI (>0.950) was good, and the model explained approximately 44% of the variance in HAZ scores (R^2=0.44).

Maternal education was significantly and positively associated with dietary diversity. One SD increase in the level of maternal education was associated with a 0.07 SD increase in dietary diversity (β = 0.07; p <0.05), but with no significant effect on breastfeeding status. Similarly, a one SD increase in household wealth was associated with a 0.18 SD increase in dietary diversity tercile (β = 0.18; p<0.001), but a one SD increase in household wealth was associated with -0.09 SD decrease in breastfeeding. A high level of maternal employment was associated with a positive, but

For peer review only - http://bmjopen.bmj.com/site/about/guidelines.xhtml

insignificant effect on dietary diversity tercile, but statistically significantly negatively associated with breastfeeding status (β=-0.07; p<0.01). Further analysis revealed that this effect on breastfeeding status was confirmed amongst younger age groups, but not in the 18-23 month age group (Supplementary table S5).

[Figure 2 Full structural path model, measuring how socioeconomic factors are associated with children's HAZ Z-scores directly and through mediators of dietary diversity and continued breastfeeding. Standardized coefficients. Cambodia (2014)]

Dietary diversity and continued breastfeeding beyond 6 months of age as mediating factors

Both hypothesised mediating variables – dietary diversity tercile and breastfeeding status – were independently and significantly associated with child HAZ score, although associations were in the opposite direction. One SD increase in dietary diversity was associated with a 0.06 SD increase in HAZ (p<0.05), whereas continued breastfeeding was associated with a -0.13 decrease in HAZ (p<0.001) (Figure 2 and Supplementary table S4). The only socioeconomic factor with a significant independent association with HAZ was household wealth, where a 1 SD increase in household wealth was associated with a 0.06 SD increase in HAZ (p<0.05).

To assess the role of dietary diversity and breastfeeding as mediators in the association between socioeconomic factors (maternal education, household wealth, maternal employment (high)) and HAZ, indirect pathways were examined (Table 2). In terms of maternal education, the standardized effects presented a case of 'inconsistent mediation',[37] where the direct and indirect effects do not have the same consistent sign. Furthermore, whilst the indirect effect is significant (p<0.001), the direct effect is not and hence, the indirect effect is larger than the total effect meaning that the effect of maternal education on HAZ is fully attributed to the indirect path which operates through household wealth, maternal employment, dietary diversity and breastfeeding status.

For peer review only

For peer review only - http://bmjopen.bmj.com/site/about/guidelines.xhtml

Table 2 Standardized coefficients of maternal education, household wealth, maternal employment and child height-for-age Z-score, with correlated residuals for dietary diversity and breastfeeding status

**Outcome**	**Direct effect (β)**	**Indirect effect (β)**	**Total effect**
HAZ			
Dietary diversity -> HAZ	0.061*	na	0.061*
Breastfeeding -> HAZ	-0.129***	-0.007	-0.136***
Maternal Education -> HAZ	-0.008	0.051***	0.043
Maternal employment (high) -> HAZ	0.054	0.039***	0.094**
Household wealth -> HAZ	0.057*	0.022**	0.080**

The significance levels are for the unstandardized solution.

*p-value<0.05, **p-value<0.01, ***p-value<0.001, na: not applicable

Although there was no direct effect of maternal employment on HAZ, statistically significant indirect effects suggested that 41% (0.039/0.094=0.41) of the effect of maternal employment on HAZ was mediated by either dietary diversity or breastfeeding status.

Household wealth was the only underlying (socioeconomic) factor with a statistically significant direct effect on HAZ, with 71% of the total effect on HAZ estimated to be direct (0.057/0.080=0.71), after controlling for dietary diversity and breastfeeding status, with the indirect effect of household wealth on HAZ an estimated 28% (0.022/0.080=0.28).

Further exploration of the specific indirect pathways between underlying (socioeconomic) factors and HAZ, through mediating variables of dietary diversity and breastfeeding, showed evidence of three indirect pathways that were statistically significant (Supplementary Table S6). High level of maternal employment had a small but significant standardized effect on HAZ, mediated by breastfeeding status of 0.01, z=2.30, p <0.05. Household wealth also had a small but statistically significant standardized effect on HAZ, mediated by both dietary diversity and breastfeeding status (0.01, z=2.13, p<0.05; 0.01, z=2.58, p<0.01 respectively).

For peer review only - http://bmjopen.bmj.com/site/about/guidelines.xhtml

Moderating effect of urban/rural residence

Exploratory analysis suggested differences in mean height-for-age Z-scores between urban and rural areas, with children in rural areas in Cambodia generally fairing worse (Supplementary figure S1). Results from an unconstrained solution (Table 3), which accounted for the moderating effects of urban/rural residence by allowing all parameters to vary, suggested that urban-rural residence had a significant moderating effect on some of the pathways in the hypothesised path analysis. Firstly, a 1 SD increase in maternal education was associated with a 0.07 SD increase in dietary diversity in rural areas (p<0.05), which was also evident in urban areas but this association was not significant. In terms of the underlying (socioeconomic) factors associated with breastfeeding, a high level of maternal employment was significantly negatively associated with breastfeeding status in rural areas, where a 1 SD increase in maternal employment was associated with a -0.07 SD decrease in breastfeeding (p<0.05), although this same association was insignificant in urban areas.

In urban areas, a 1 SD increase in household wealth was associated with a statistically significant decrease in breastfeeding by -0.26 SD (*p<0.01),* whereas there was a smaller, insignificant association observed in rural areas, suggesting a strong moderating effect of urban-rural residence on this pathway. Finally, the results suggested that both household wealth and dietary diversity were positively associated with HAZ through a statistically significant direct pathway, a finding which was not observed in urban areas (0.10, p<0.01; 0.07, p<0.05 respectively).

For peer review only

Table 3 Summary table for multiple-group analysis, unstandardized (B) and standardized coefficients (β)

**Path**	**Unconstrained solution**			
	**Rural (*n*=1001)**		**Urban (*n*=364)**	
	**B**	**β**	**B**	**β**
Maternal employment (high)				
Maternal education	0.157***	0.231***	0.197***	0.245***
Household wealth				
Maternal employment (high)	1.091***	0.325***	0.780***	0.277***
Maternal education	0.711***	0.312***	0.686***	0.305***
Dietary diversity				
Maternal employment (high)	0.057	0.032	0.161	0.101
Household wealth	0.084***	0.159***	0.128***	0.225***
Breastfeeding	-0.128*	-0.072*	-0.284**	-0.178**
Maternal education	0.087*	0.072*	0.070	0.054
Breastfeeding				
Maternal employment (high)	-0.072*	-0.072*	-0.065	-0.065
Household wealth	-0.007	-0.024	-0.093***	-0.262***
Maternal education	-0.010	-0.015	0.044	0.054
HAZ				
Maternal employment (high)	0.116	0.036	0.054	0.017
Household wealth	0.095**	0.099**	-0.004	-0.004
Dietary diversity	0.119*	0.066*	0.031	0.015
Breastfeeding	-0.370**	-0.115**	-0.549**	-0.168**
Maternal education	-0.135	-0.062	0.196	0.075
**χ^2 overall (df)**	81.69(40)			
**RMSEA**	0.039			
**CFI**	0.962			

*p-value<0.05, **p-value<0.01, ***p-value<0.001. **Bold:** Unconstrained parameter in the constrained model.

For peer review only

For peer review only - http://bmjopen.bmj.com/site/about/guidelines.xhtml

DISCUSSION

The current study extends previous research examining factors associated with child stunting, to present a robust statistical model, which reflects the complex interactions between variables associated with children's height-for-age. Structural path analysis allows for the replication of a theoretical framework with underlying and proximate factors, to examine both the indirect and direct effect of factors known to be associated with child stunting. The structural path model presented could be applicable to other low- and middle-income country contexts and could examine whether our proposed pathways to stunting are consistent across geographical contexts.

Overall, the results of this study confirmed that improvements in socioeconomic status such as maternal education, household wealth and participation in employment, were associated with an increase in children's dietary diversity, but a decrease in continued breastfeeding beyond 6 months of age. The findings also suggested that dietary diversity and continued breastfeeding mediate the associations between socioeconomic status and height-for-age Z-scores. High level of maternal employment had a small but significant association with HAZ that was mediated by breastfeeding status, while the significant association between household wealth and HAZ was mediated by both dietary diversity and breastfeeding status. This suggests that interventions to improve child nutritional status should make due consideration for the underlying socioeconomic drivers which may influence infant and young child feeding practices.

The independent effect of maternal education on dietary diversity was small but significant, consistent with the findings of other studies conducted in low- and middle-income countries,[38-41] in addition to studies which confirmed a strong association between increasing household wealth and improvements in dietary diversity.[15, 42] The significant pathway between breastfeeding and dietary diversity demonstrated an additional, interesting observation, that continued breastfeeding beyond 6 months of age, was associated with a less diverse diet, even after adjusting for age. This could be indicative of the situation in poorer households where prolonged breastfeeding may be practiced to make up for the lack of complementary foods, as suggested from a study of feeding practices in Thailand.[9]

In light of the nutrition transition, which has been occurring in many low- and middle-income countries, Popkin and Bisgrove[43] reported that duration of breastfeeding was declining in urban areas as a result of the increased maternal education and employment opportunities that arise from urbanisation. Furthermore, the negative association between increasing household wealth and continued breastfeeding is perhaps underscored by the increasing trend in use of breast milk substitutes among children aged over six months of age in Cambodia.[44]

For peer review only - http://bmjopen.bmj.com/site/about/guidelines.xhtml

Our study confirmed that dietary diversity and continued breastfeeding mediate associations between socioeconomic status and child stunting, however these findings were only significant for pathways originating from maternal employment and household wealth. The results of this study suggested a positive and direct association between Cambodian children's dietary diversity and height-for-age z-scores, which has also been supported by other studies conducted in Cambodia.[45, 46] Moreover, dietary diversity was a significant mediator of the association between household wealth and HAZ suggesting that the positive effect of increasing household wealth can partially be explained by improved dietary diversity. This highlights the need to prioritise targeted interventions within the poorest households to improve dietary diversity to prevent child stunting. A further 71% of the total effect of household wealth on HAZ was found to be direct, suggesting the importance of other mediating factors not included in this model but known to be associated with child stunting, such as access to health care, WASH facilities and immunization status.

On the other hand, breastfeeding after age 6 months was associated with a negative, direct effect on children's HAZ in Cambodia in the path model that adjusted for age of the child. This was also noted by Arimond and Ruel,[27] in a study using Cambodian DHS data from 2000, and in a review of 13 studies by Grummer-Strawn.[47] A negative effect of breastfeeding beyond age six months on children's height-for-age status has been discussed by many researchers. One proposed explanation is reverse causality, whereby the decision to wean is related to mother's perceptions of size and stature of the child, hence malnourished children may be more likely to be breastfed for longer durations.[48] However, as Martin[49] points out, due to the cross-sectional nature of structural path analysis, it is not possible to account for the direction in the relationship between prolonged breastfeeding and HAZ. In terms of the indirect effects, structural path analysis revealed that breastfeeding was a small but significant mediator in the association between maternal employment and HAZ, as well as household wealth and HAZ.

Finally, this study highlighted the moderating effect of urban-rural residence, in particular, the differences in individual factors that are associated with specific pathways to HAZ, but also that the strength of these associations varies by urban-rural residence. This analysis shed new light on the differences in pathways to stunting by urban-rural residence in Cambodia, and so far is one of the first studies which has separately analysed the effect of urban-rural residence. Results from simultaneous path analysis by urban-rural residence demonstrated the moderating effect of this variable, stressing the importance of designing nutritional interventions which account for the differences in the determinants of stunting between urban and rural areas. This is especially important for low- and middle-income contexts such as Cambodia, where there are distinct urban-

For peer review only - http://bmjopen.bmj.com/site/about/guidelines.xhtml

rural differentials in the incidence of stunting and where rapid urbanisation is causing a shift in the geographical patterning of stunting.

Moreover, as shown by an unconstrained model where all parameters were permitted to vary by urban-rural residence, maternal education was a significant and positive predictor of dietary diversity in rural areas, but not in urban areas. Although this difference between rural and urban areas was not statistically significant, it may provide evidence in support of community-based interventions that target nutrition education programmes amongst rural mothers. For example, a successful FAO-supported agricultural intervention known as MALIS (Improving market linkages for smallholder farmers) combined a nutritional education programme focused on infant and young child feeding (IYCF) practices in rural Cambodia, resulting in a significant increase in child dietary diversity, specifically increased consumption of vitamin A-rich foods and other fruits and vegetables[50]

Although increasing household wealth was significantly negatively associated with breastfeeding status in the full structural path model, further analysis controlling for the moderating effect of urban-rural residence revealed a statistically significant difference in the effect of household wealth on breastfeeding status between urban and rural areas. Household wealth was significantly and negatively associated with breastfeeding status in urban areas in both an unconstrained and constrained solution, which controlled for this moderating effect, suggesting extreme intra-urban inequality in breastfeeding practices between the urban-poor and the urban-rich. This finding emphasises the fact that within urban areas interventions to improve breastfeeding practices should be mindful of the socioeconomic disparities in practices that occur even within urban spaces.

STRENGTHS AND LIMITATIONS

This study used population-based data from the most recent large nationally representative household survey from Cambodia, and our findings are generalizable to Cambodia. DHS data uses standard instruments to measure height by professionally trained investigators, which ensures accuracy of anthropometric measurements, however, the way in which continued breastfeeding and dietary diversity are reported using current-status data (from a 24 hour recall questionnaire) may introduce measurement bias, although this would apply equally to stunted and non-stunted children. Application of a causal hypothetical model to explain possible pathways to children's linear growth, presents the first study of its kind using Cambodian DHS data, and lays the foundation for similar studies in other geographical contexts. However, the cross-sectional nature of DHS data and the use of structural path analysis, typically a form of causal modelling, requires that the results be

For peer review only - http://bmjopen.bmj.com/site/about/guidelines.xhtml

interpreted with some caution. Although structural path models may offer benefits over multivariable fixed effects models in allowing for complex interactions between variables, the theoretical model was built iteratively based on prior theory and thus, is by no means an exhaustive representation of the pathways to children's HAZ scores.

CONCLUSIONS

The theoretical structural path model presented in this study proposes a plausible illustration of the pathways associated with children's height-for-age in the Cambodian context. Overall, results confirmed that children's dietary diversity and continued breastfeeding status mediate the associations between socioeconomic status and linear growth, at least for pathways from maternal employment and household wealth. Analysis suggests that participation in maternal employment presents a significant barrier to continued breastfeeding beyond age six months of age, emphasising a need for maternity legislation that supports optimal breastfeeding practices, in line with WHO recommendations that infants be exclusively breastfed for six months, followed by continued breastfeeding in addition to complementary feeding up to two years of age.[25] Improvements to maternity legislation should also be inclusive of women working in the informal sector (of which 96% of employed women in Cambodia participate). Further simultaneous path analysis of the proposed model highlighted the moderating effect of urban-rural residence, emphasizing the need for nutrition interventions which not only target women and children of low socioeconomic status, but which also account for the different ways in which poverty is experienced in urban and rural areas.

CONTRIBUTORS

CMH conceptualised the study, prepared the dataset for research and conducted statistical analysis under the supervision of MLN and SSP. MLN and SSP contributed to the interpretation and revised paper for intellectual content. CMH, MLN and SPP conducted the final review and revised the manuscript for submission.

COMPETING INTERESTS

None declared.

For peer review only

FUNDING

This research was carried out as a part of a doctoral research project, supported by the UK Economic and Social Research Council (grant number ES/J500161/1). The Economic and Social Research Council had no role in the design, analysis or writing of this article.

DATA SHARING

No additional data available

ETHICS STATEMENT

This study is based on publicly available DHS data. CMH was granted access to the data by the MEASURE DHS/ICF International, Rockville, Maryland, USA.

REFERENCES

1. [dataset] United Nations Children's Fund (UNICEF). Data from: Stunting (Survey Estimates, National and Disaggregated). April 11, 2021. https://data.unicef.org/topic/nutrition/malnutrition/
2. Black RE, Victora CG, Walker SP, et al. Maternal and child undernutrition and overweight in low-income and middle-income countries. *The Lancet* 2013;382:427–451.
3. Dewey KG, Mayers DR. Early child growth: How do nutrition and infection interact? *Matern Child Nutr* 2011;*7*(Suppl 3):129–142.
4. Black RE, Allen LH, Bhutta ZA, et al. Maternal and child undernutrition: global and regional exposures and health consequences. *The Lancet* 2008;371:243–260
5. Victora CG, Adair L, Fall C, et al. Maternal and child undernutrition: consequences for adult health and human capital. *The Lancet* 2008;371:340–357.
6. Ozaltin E, Hill K, Subramanian SV. Association of maternal stature with offspring mortality, underweight, and stunting in low- to middle-income countries. *JAMA*, 2010;303:1507-1516.

For peer review only - http://bmjopen.bmj.com/site/about/guidelines.xhtml

7. Mutunga M, Frison S, Rava M, et al. The Forgotten Agenda of Wasting in Southeast Asia: Burden, Determinants and Overlap with Stunting: A Review of Nationally Representative Cross-Sectional Demographic and Health Surveys in Six Countries. *Nutrients* 2020;12:559.
8. Krasevec J, An X, Kumapley, R, et al. Diet quality and risk of stunting among infants and young children in low- and middle-income countries. *Matern Child Nutr* 2017;13(Suppl 2):1–11.
9. Cetthakrikul N, Topothai C, Suphanchaimat R, et al. Childhood stunting in Thailand: When prolonged breastfeeding interacts with household poverty. *BMC Pediatr* 2018;18: 1–9
10. Marquis GS, Habicht JP, Lanata CF, et al. Association of breastfeeding and stunting in Peruvian toddlers: An example of reverse causality. *Int J Epidemiol* 1997;26:349–356.
11. Boyle M, Georgiades K, Racine Y, et al. The influence of economic development level, household wealth and maternal education on child health in the developing world. *Soc Sci Med* 2006;63:2242–2254.
12. Caldwell JC. Education as a Factor in Mortality Decline An Examination of Nigerian Data. *Popul Stud (NY)* 1979;33:395–413.
13. Caldwell JC. How is greater maternal education translated into lower child mortality? *Health Transit Rev* 1994;4:224–229.
14. Popkin BM, Solon FS. Income, time, the working mother and child nutriture. *J Trop Pediatr* 1976;22:156–166.
15. Smith LC, Ruel MT, Ndiaye A. Why is child malnutrition lower in urban than in rural areas? Evidence from 36 developing countries. *World Dev* 2005;33:1285–1305.
16. Harvey CM, Newell ML, Padmadas SS. Socio-economic differentials in minimum dietary diversity among young children in minimum dietary diversity among young children in South-East Asia: evidence from Demographic and Health Surveys. *Public Health Nutr* 2018;21:3048-3057.
17. Menon P, Ruel MT, Morris SS. Socio-economic differentials in child stunting are consistently larger in urban than rural areas. *Food Nutr Bull* 2000;21:282-289.
18. Hanieh S, Ha TT, De Livera AM, et al. Antenatal and early infant predictors of postnatal growth in rural Vietnam: A prospective cohort study. *Arch Dis Child* 2015;100:165–173.
19. Lander RL, Williams SM, Costa-Ribeiro H, et al. Understanding the complex determinants of height and adiposity in disadvantaged daycare preschoolers in Salvador, NE Brazil through structural equation modelling. *BMC Public Health* 2015;15:1–8.

For peer review only - http://bmjopen.bmj.com/site/about/guidelines.xhtml

20. Cunningham K, Ferguson E, Ruel M, et al. Water, sanitation, and hygiene practices mediate the association between women's empowerment and child length-for-age z-scores in Nepal. *Matern Child Nutr* 2018;15:1–9.

21. [dataset] National Institute of Statistics, Directorate General for Health, & ICF International. Data from: Cambodia Demographic and Health Survey 2014. The DHS Program, August 17, 2018. https://dhsprogram.com/methodology/survey/survey-display-464.cfm

22. World Health Organization (WHO). *WHO Multicentre Growth Reference Study Group. WHO Child Growth Standards: Length/height-for-age, weight-for-age, weight-for-length, weight-for-height and body mass index-for-age: Methods and development*. Geneva: WHO, 2006. https://www.who.int/publications/i/item/924154693X

23. Stewart CP, Iannotti L, Dewey KG, et al. Contextualising complementary feeding in a broader framework for stunting prevention. *Matern Child Nutr* 2013;9(Suppl 2): 27–45.

24. Wamani H, Åstrøm AN, Peterson S, et al. Infant and young child feeding in Western Uganda: Knowledge, practices and socio-economic correlates. *J Trop Pediatr* 2005;51:356–361.

25. World Health Organization (WHO) Continued breastfeeding for healthy growth and development of children [Internet]. WHO, 2017. Available: https://www.who.int/elena/titles/bbc/continued_breastfeeding/en/ [Accessed 02 Sept 2019].

26. World Health Organization (WHO). *Indicators for assessing infant and young child feeding practices, Part 1: Definitions*. Geneva: WHO, 2008. https://apps.who.int/iris/bitstream/handle/10665/43895/9789241596664_eng.pdf;jsessionid=42E6F2DE11CA32FA51E4636A4301253A?sequence=1

27. Arimond M, Ruel MT. Dietary diversity is associated with child nutritional status: evidence from 11 demographic and health surveys. *J Nutr* 2004;*134:*2579–2585.

28. Rutstein SO. The DHS Wealth Index. DHS Working Paper. *USAID* 2008;60 [Working Paper] https://www.researchgate.net/publication/238706094_The_DHS_Wealth_Index_Approaches_for_Rural_and_Urban_Areas

29. Nankinga O, Kwagala B, Walakira EJ. Maternal employment and child nutrition status in Uganda. *PLoS One* 2019;14(e0226720). https://journals.plos.org/plosone/article?id=10.1371/journal.pone.0226720 (accessed 5 August 2020).

30. Debela BL, Gehrke E, Qaim M. Links between Maternal Employment and Child Nutrition in Rural Tanzania. *Am J Agric Econ* 2020;103:812-830.

For peer review only - http://bmjopen.bmj.com/site/about/guidelines.xhtml

For peer review only

31. Sebayang SK, Efendi F, Astutik E. Women's empowerment and the use of antenatal care services: analysis of demographic health surveys in five Southeast Asian countries. *Women Health* 2017;59:1-17.
32. [software] StataCorp. *Stata Statistical Software: Release 15*. StataCorp LLC 2017.
33. Acock A. Discovering Structural Equation Modeling Using Stata. Texas, US: Stata Press 2013.
34. Grace J, Bollen K. Interpreting the Results from Multiple Regression and Structural Equation Models. *Bulletin Ecological Society of America* 2005;86:283–295.
35. Hox JJ, Bechger T. An Introduction to Structural Equation Modeling. *Family Science Review* 1999;11:354–373.
36. Schreiber JB, Nora A, Stage FK. Reporting Structural Equation Modelling and Confirmatory Factor Analysis Results: A Review. *J Educ Res* 2006;99:323–338.
37. Mackinnon DP, Fairchild AJ, Fritz MS. Mediation Analysis. *Annu Rev Psychol* 2007;58:1–22.
38. Na M, Aguayo VM, Arimond M, et al. Trends and predictors of appropriate complementary feeding practices in Nepal: An analysis of national household survey data collected between 2001 and 2014. *Matern Child Nutr* 2018;14(Suppl 4):1–13.
39. Patel A, Pusdekar Y, Badhoniya N, et al. Determinants of inappropriate complementary feeding practices in young children in India: secondary analysis of National Family Health Survey 2005 – 2006. *Matern Child Nutr* 2012;8(Suppl 1):28–44.
40. Kabir I, Khanam M, Agho KE, et al. Determinants of inappropriate complementary feeding practices in infant and young children in Bangladesh: secondary data analysis of Demographic Health Survey 2007. *Matern Child Nutr* 2012;8(Suppl 1):11–27.
41. Senarath U, Agho KE, Akram DES, et al. Comparisons of complementary feeding indicators and associated factors in children aged 6-23 months across five South Asian countries. *Matern Child Nutr* 2012;8(Suppl 1):89–106.
42. Iannotti LL, Robles M, Pachón H, et al. Food Prices and Poverty Negatively Affect Micronutrient Intakes in Guatemala. *J Nutr* 2012;142:1568–1576.
43. Popkin BM, Bisgrove EZ. Nutrition and Urbanization (Part 2): Urbanization and Nutrition in low-Income countries. *Food Nutr Bull* 1988;10:1–22.
44. Prak S, Dahl MI, Oeurn S, et al. Breastfeeding Trends in Cambodia, and the Increased Use of Breast-Milk Substitute—Why Is It a Danger? *Nutrients* 2014;6:2920–2930.
45. Greffeuille V, Sophonneary P, Laillou A, et al. Persistent inequalities in child undernutrition in Cambodia from 2000 until today. *Nutrients* 2016;8:1–17.
46. Darapheak C, Takano T, Kizuki M, et al. Consumption of animal source foods and dietary diversity reduce stunting in children in Cambodia. *Int Arch Med* 2013;6:1–11.

For peer review only

47. Grummer-Strawn L. Regression analysis of current-status data: an application to breast-feeding. *J Am Stat Assoc* 1993;88:758–765.

48. Simondon K, Costes R, Delaunay V, et al. Children's height, health and appetite influence mothers' weaning decisions in rural Senegal. *Int J Epidemiol* 2001;30:476–481.

49. Martin RM. Commentary: Does breastfeeding for longer cause children to be shorter? *Int J Epidemiol* 2001;30:481–484.

50. Reinbott A, Schelling A, Kuchenbecker J, et al. Nutrition education linked to agricultural interventions improved child dietary diversity in rural Cambodia. *Br J Nutr* 2016;116:1457-1468.

For peer review only

For peer review only - http://bmjopen.bmj.com/site/about/guidelines.xhtml

Figure 1 Trends in pre-marital conception among ever-married/in union women 20-24 years who gave birth before 18 years, by survey year

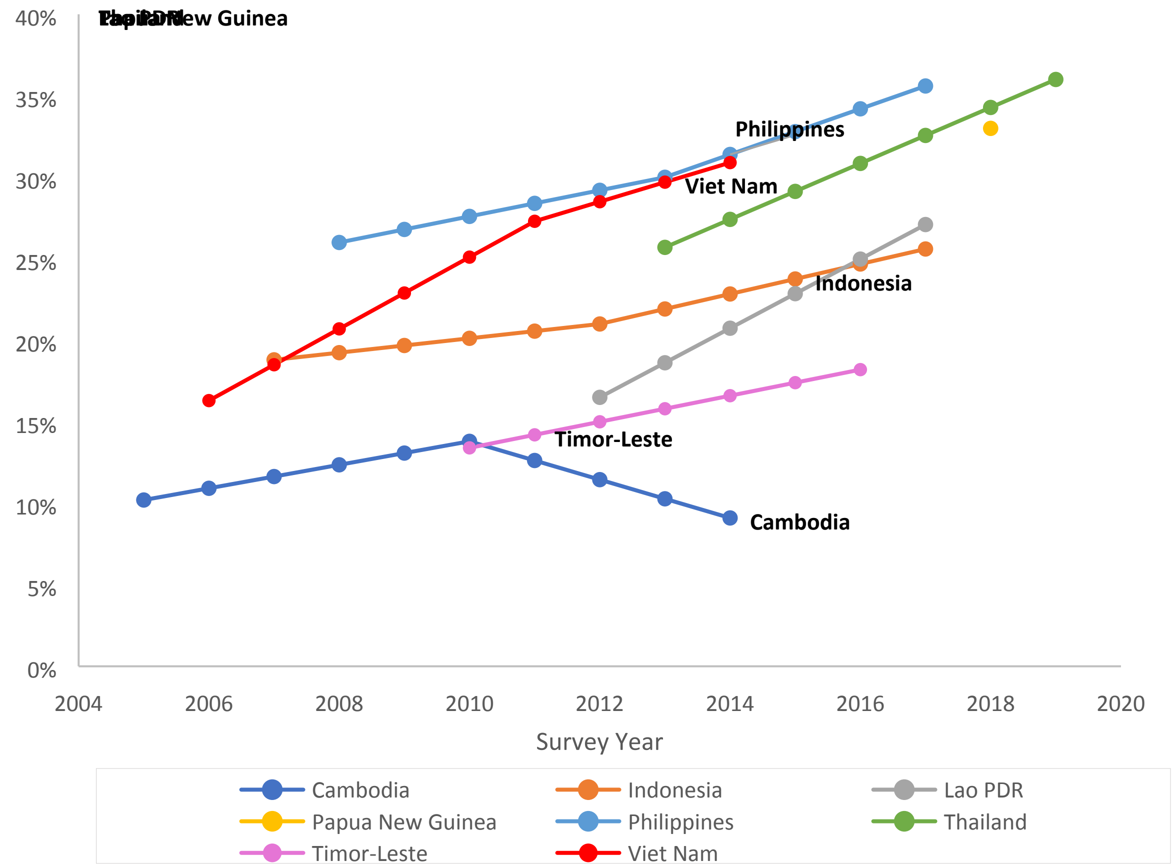

Note: Trend data for Papua New Guinea not presented as only one survey year is available.

For peer review only

Figure 1 Percentage of ever-married/in union women who conceived premaritally and were married/in union by the time of adolescent birth (before age 18 years)

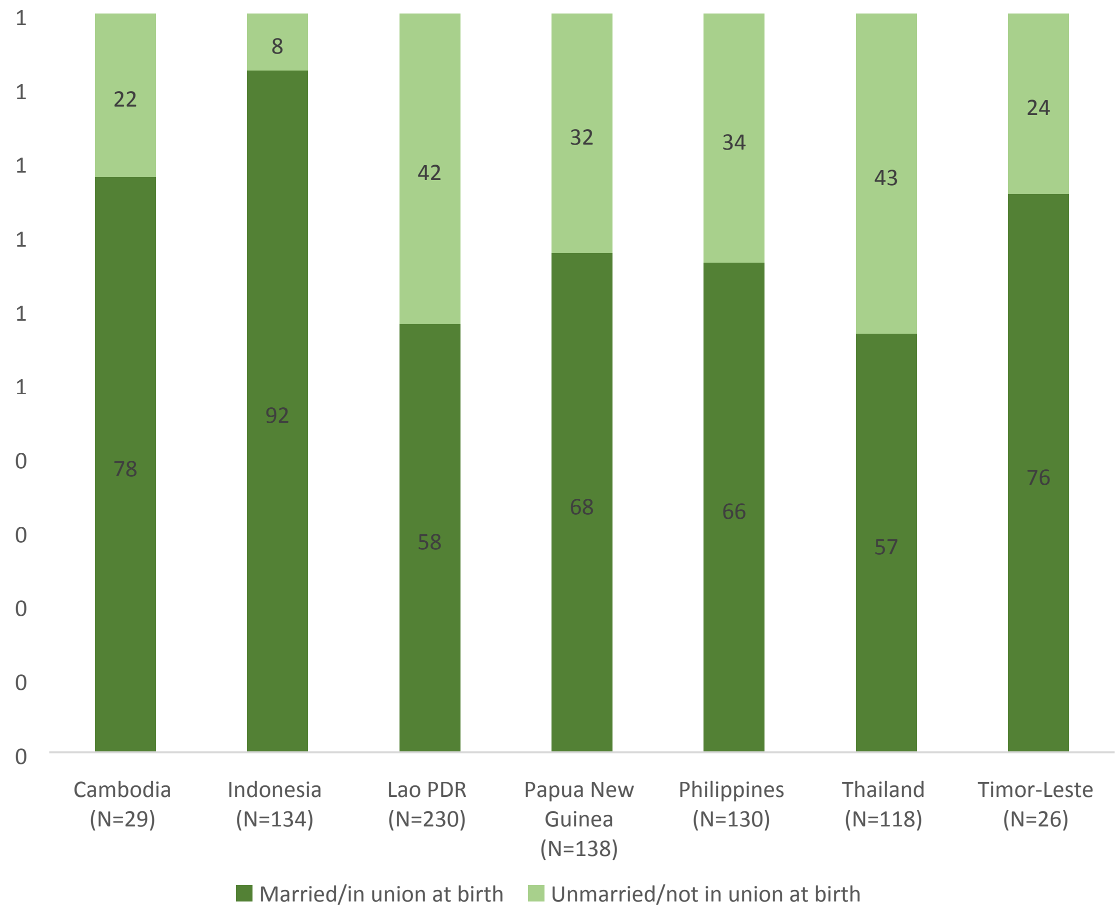

Note: Marriage is defined as being in a formal marriage/union/co-habitation with a partner.
Sample size for Cambodia (n=29) and Timor-Leste (n=26) is relatively small and should be interpreted with some caution. Viet Nam is not shown due to a small sample size <25.

Uses most recent survey data from Cambodia (DHS 2014); Indonesia (DHS 2017); Lao PDR (MICS 2017); Papua New Guinea (DHS 2016-18); Philippines (DHS 2017); Thailand (MICS 2019); Timor-Leste (DHS 2016); Vietnam (MICS 2013-14).

only

Supplementary Table S1. Sample size selection: children aged 6 to <24 months with plausible anthropometric measurements, Cambodia (2014)

	n
Children 6-23.9 months	2,127
Children 6-23.9 months with anthropometric measurements	1,397
**Children 6-23.9 months with plausible† anthropometric measurements**	**1,365**

† Plausible HAZ scores should fall between -6 and +6 standard deviations, according to the WHO SD flag limits (WHO, 2006)

Supplementary Table S2. Distribution of dietary diversity tercile by child's age, adjusted estimates, Cambodia (2014)

	**Age in months (*n* = 1,381)**								
	**6-8 (*n*=242)**		**9-11 (*n*=208)**		**12-17 (*n*=463)**		**18-23 (*n*=468)**		**6-23 (*n*=1,381)**
**Dietary Diversity Tercile**	%	**DDS range**	**%**	**DDS range**	**%**	**DDS range**	**%**	**DDS range**	**%**
Low	44.9	0-1 groups	41.0	0-2 groups	49.5	0-3 groups	37.0	0-3 groups	**43.2**
Middle	17.9	2 groups	43.0	3-4 groups	26.2	4 groups	45.4	4-5 groups	**33.8**
High	37.2	≥ 3 groups	16.0	≥ 5 groups	24.4	≥ 5 groups	17.6	≥ 6 groups	**23.1**

For peer review only

Supplementary Table S3. Association between IYCF indicators, socio-demographic factors and mean height-for-age Z-scores in children aged 6-23 months in Cambodia (2014), (estimates adjusted for survey design using appropriate sample weights)

Indicator	%	N	Mean HAZ	Std. deviation	95% CI for mean	Test statistic
Inherent (biological) factors						
Age (months)						
6-8	17.5	242	-0.55	1.25	-0.75, -0.35	F(3, 1362)=22.20, *p*=<0.001
9-11	15.1	208	-0.92	1.48	-1.14, -0.70	
12-17	33.5	463	-1.41	1.23	-1.54, -1.28	
18-23	33.9	468	-1.46	1.32	-1.60, -1.31	
Gender						
Male	52.0	718	-1.28	1.34	-1.39, -1.16	F(1, 1364)=3.33, *p*=0.068
Female	48.0	663	-1.12	1.35	-1.24, -1.00	
Birth weight						
< 2.5kg	6.0	83	-1.48	1.50	-1.85, -1.11	F(3, 1362)=5.65, *p*=<0.001
2.5 < 4.0kg	83.4	1152	-1.16	1.33	-1.25, -1.07	
≥ 4.0kg	3.4	46	-0.82	1.10	-1.17, -0.46	
Missing/not weighed	7.2	100	-1.63	1.38	-1.91, -1.36	
Maternal Height						
Normal stature (≥145cm)	94.8	1308	-1.16	1.34	-1.25, -1.08	F(1, 1362)=20.26, *p*=<0.001
Short stature (<145cm)	5.2	71	-1.90	1.36	-2.21, -1.59	
Maternal BMI						
<18.5	15.6	215	-1.50	1.22	-1.68, -1.32	F(2, 1360)=5.72, *p*=0.003
18.5-24.99	69.2	952	-1.17	1.39	-1.27, -1.06	
≥ 25.0	15.2	209	-1.09	1.24	-1.31, -0.86	
Proximate factors						
Morbidity						
No	56.5	781	-1.16	1.40	-1.27, -1.05	F(1, 1364)=1.45, *p*=0.229
Yes	43.5	601	-1.26	1.28	-1.39, -1.13	
Breastfeeding						
No	29.9	413	-1.15	1.39	-1.30, -0.99	F(1, 1364)=0.66, *p*=0.4178
Yes	70.1	968	-1.22	1.33	-1.32, -1.12	

For peer review only

Indicator	%	N	Mean HAZ	Std. deviation	95% CI for mean	Test statistic
Bottle feeding						
No	66.0	911	-1.27	1.33	-1.38, -1.16	F(1, 1363)=5.39, *p*=0.020
Yes	34.0	469	-1.07	1.37	-1.20, -0.93	
Dietary Diversity Tercile						
Low	43.2	596	-1.30	1.20	-1.42, -1.17	F(2, 1363)=7.80, *p*=<0.001
Middle	33.8	467	-1.29	1.46	-1.44, -1.13	
High	23.1	318	-0.90	1.41	-1.07, -0.73	
Minimum meal frequency						
No	27.4	378	-1.32	1.38	-1.48, -1.16	F(1, 1364)=2.93, *p*=0.087
Yes	72.6	1003	-1.16	1.33	-1.25, -1.06	
Minimum acceptable diet						
No	68.4	945	-1.23	1.32	-1.33, -1.13	F(1, 1364)=1.13, *p*=0.287
Yes	31.6	436	-1.13	1.41	-1.29, -0.98	
Distal (underlying) factors						
Highest education level						
No education	12.0	166	-1.26	1.45	-1.50, -1.02	F(1, 1364)=6.90, *p*=0.009
Primary	55.6	768	-1.28	1.27	-1.40, -1.16	
Secondary/Higher	32.4	447	-1.04	1.42	-1.18, -0.91	
Labour force participation						
Not working	30.5	421	-1.19	1.22	-1.34, -1.04	F(2, 1359)=8.34, *p*=<0.001
Low	37.5	517	-1.40	1.42	-1.54, -1.26	
High	32.0	441	-0.98	1.34	-1.12, -0.84	
Household Wealth Index						
Poorest	25.4	345	-1.48	1.49	-1.65, -1.32	F(4, 1347)=7.53, *p*=<0.001
Poorer	18.2	248	-1.32	1.41	-1.50, -1.14	
Middle	19.9	271	-1.23	1.18	-1.41, -1.05	
Richer	17.7	241	-1.00	1.21	-1.20, -0.80	
Richest	18.8	256	-0.86	1.17	-1.04, -0.67	
Urban/rural residence						
Rural	86.4	1193	-1.25	1.23	-1.34, -1.16	F(1,1364)=13.04, *p*=<0.001
Urban	13.6	188	-0.88	1.97	-1.06, -0.70	
Sex of household head						
Male	77.0	1064	-1.20	1.33	-1.29, -1.10	F(1, 1364)=0.01, *p*=0.920
Female	23.0	317	-1.21	1.39	-1.40, -1.02	

For peer review only

Supplementary Table S4 Unstandardized and standardized results of the full structural path model examining the association between selected socioeconomic factors, mediating feeding practices and child HAZ, Cambodia (2014)

Path	Unstandardized (β)	Standardized (β)	R^2
Maternal employment (high)			0.15
Maternal education	0.167***	0.234***	
Household wealth			0.24
Maternal employment (high)	0.993***	0.327***	
Maternal education	0.706***	0.326***	
Dietary diversity			0.11
Maternal employment (high)	0.088	0.053	
Household wealth	0.097***	0.176***	
Breastfeeding	-0.191***	-0.111***	
Maternal education	0.081*	0.068*	
Breastfeeding			0.24
Maternal employment (high)	-0.071**	-0.073**	
Household wealth	-0.027**	-0.086**	
Maternal education	0.004	0.005	
HAZ			0.12
Maternal employment (high)	0.168	0.054	
Household wealth	0.058*	0.057*	
Dietary diversity	0.113*	0.061*	
Breastfeeding	-0.413***	-0.129***	
Maternal education	-0.019	-0.008	
**Overall R^2**			0.44
**χ^2 (df)**	65.70 (25)		
**RMSEA**	0.035		
**CFI**	0.972		

*p-value<0.05, **p-value<0.01, ***p-value<0.001

For peer review only

Supplementary Table S5 Proportion of women who reported breastfeeding at time of survey, by level of maternal participation in employment and child age group, Cambodia (2014)

	Age in months (*n*=1,365)				
	**6-8**	**9-11**	**12-17**	**18-23**	**6-23**
Not working or maternal employment (low)	95.3	93.6	85.7	42.3	75.5
Maternal employment (high)	82.6	78.8	72.0	34.0	59.0
X^2 test	X^2=8.52(1), *p*=0.004	X^2=7.06(1), *p*=0.008	X^2=7.52(1), *p*=0.006	X^2=2.03(1), *p*=0.155	X^2=24.72(1), *p*<0.001

Supplementary Table S6 Results of non-linear combinations for testing specific standardized indirect effects

**Path**	**Indirect Effect**			
	**Coef.**	**z**	**P-value**	**95% CI**
Maternal education -> Dietary diversity -> HAZ	0.004	1.63	0.104	(-0.00, 0.01)
Maternal education -> Breastfeeding -> HAZ	-0.001	-0.20	0.842	(-0.01, 0.01)
Maternal employment (high) -> Dietary diversity -> HAZ	0.003	1.41	0.159	(-0.00, 0.01)
Maternal employment (high) -> Breastfeeding -> HAZ	0.009	2.30	0.022	(0.00, 0.02)
Household wealth -> Dietary diversity -> HAZ	0.011	2.13	0.033	(0.00, 0.02)
Household wealth -> Breastfeeding -> HAZ	0.011	2.58	0.01	(0.00, 0.02)

For peer review only

Supplementary Figure S1 Mean height-for-age Z-scores by child age group and urban/rural residence (upper SD represented by error bar)

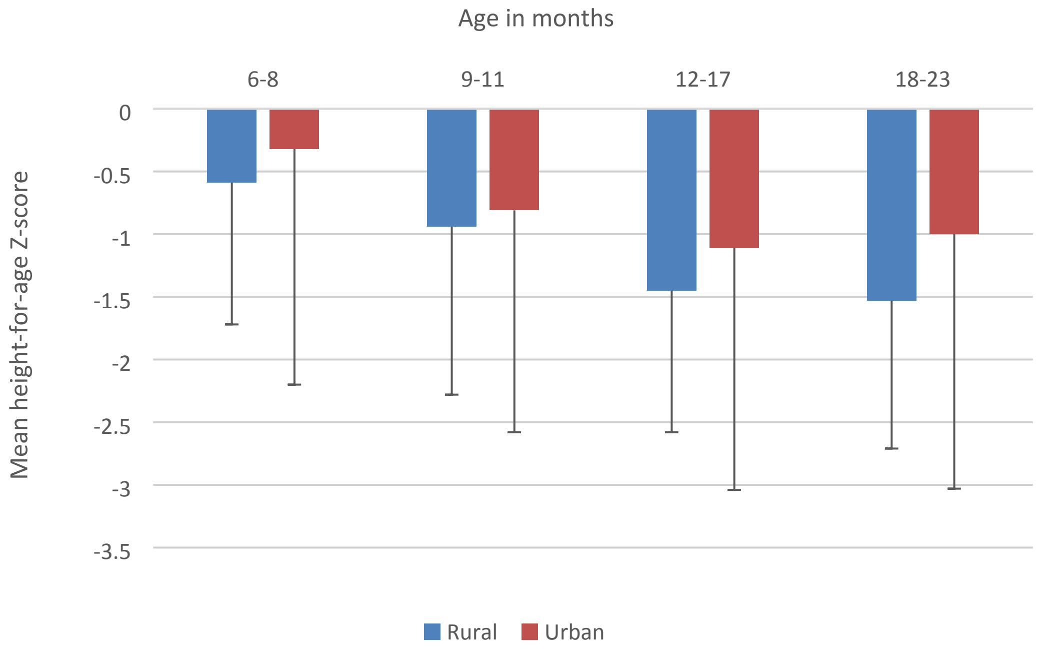

For peer review only

STROBE Statement—Checklist of items that should be included in reports of ***cross-sectional studies***

	Item No	Recommendation	Page No
**Title and abstract**	1	(*a*) Indicate the study's design with a commonly used term in the title or the abstract	1
		(*b*) Provide in the abstract an informative and balanced summary of what was done and what was found	2
**Introduction**			
Background/rationale	2	Explain the scientific background and rationale for the investigation being reported	4
Objectives	3	State specific objectives, including any prespecified hypotheses	5
**Methods**			
Study design	4	Present key elements of study design early in the paper	5
Setting	5	Describe the setting, locations, and relevant dates, including periods of recruitment, exposure, follow-up, and data collection	6
Participants	6	(*a*) Give the eligibility criteria, and the sources and methods of selection of participants	5
Variables	7	Clearly define all outcomes, exposures, predictors, potential confounders, and effect modifiers. Give diagnostic criteria, if applicable	6/7
Data sources/ measurement	8*	For each variable of interest, give sources of data and details of methods of assessment (measurement). Describe comparability of assessment methods if there is more than one group	*6/7*
Bias	9	Describe any efforts to address potential sources of bias	7-8
Study size	10	Explain how the study size was arrived at	
Quantitative variables	11	Explain how quantitative variables were handled in the analyses. If applicable, describe which groupings were chosen and why	5-6
Statistical methods	12	(*a*) Describe all statistical methods, including those used to control for confounding	7-9
		(*b*) Describe any methods used to examine subgroups and interactions	9
		(*c*) Explain how missing data were addressed	6
		(*d*) If applicable, describe analytical methods taking account of sampling strategy	6
		(*e*) Describe any sensitivity analyses	8/12-13
**Results**			
Participants	13*	(a) Report numbers of individuals at each stage of study—eg numbers potentially eligible, examined for eligibility, confirmed eligible, included in the study, completing follow-up, and analysed	5-6
		(b) Give reasons for non-participation at each stage	5-6
		(c) Consider use of a flow diagram	Supplementary S1
Descriptive data	14*	(a) Give characteristics of study participants (eg demographic, clinical, social) and information on exposures and potential confounders	10

For peer review only - http://bmjopen.bmj.com/site/about/guidelines.xhtml

		(b) Indicate number of participants with missing data for each variable of interest	10-12
Outcome data	15*	Report numbers of outcome events or summary measures	6
Main results	16	(*a*) Give unadjusted estimates and, if applicable, confounder-adjusted estimates and their precision (eg, 95% confidence interval). Make clear which confounders were adjusted for and why they were included	13/16
		(*b*) Report category boundaries when continuous variables were categorized	10
		(*c*) If relevant, consider translating estimates of relative risk into absolute risk for a meaningful time period	
Other analyses	17	Report other analyses done—eg analyses of subgroups and interactions, and sensitivity analyses	15-16
**Discussion**			
Key results	18	Summarise key results with reference to study objectives	17
Limitations	19	Discuss limitations of the study, taking into account sources of potential bias or imprecision. Discuss both direction and magnitude of any potential bias	19-20
Interpretation	20	Give a cautious overall interpretation of results considering objectives, limitations, multiplicity of analyses, results from similar studies, and other relevant evidence	17-20
Generalisability	21	Discuss the generalisability (external validity) of the study results	17-20
**Other information**			
Funding	22	Give the source of funding and the role of the funders for the present study and, if applicable, for the original study on which the present article is based	20

*Give information separately for exposed and unexposed groups.

Note: An Explanation and Elaboration article discusses each checklist item and gives methodological background and published examples of transparent reporting. The STROBE checklist is best used in conjunction with this article (freely available on the Web sites of PLoS Medicine at http://www.plosmedicine.org/, Annals of Internal Medicine at http://www.annals.org/, and Epidemiology at http://www.epidem.com/). Information on the STROBE Initiative is available at www.strobe-statement.org.

For peer review only

BMJ Open

Maternal socioeconomic status and infant feeding practices underlying pathways to child stunting in Cambodia: structural path analysis using cross-sectional population data

Journal:	*BMJ Open*
Manuscript ID	bmjopen-2021-055853.R1
Article Type:	Original research
Date Submitted by the Author:	16-Jun-2022
Complete List of Authors:	Harvey, Chloe; Independent Consultant<br>Newell, Marie-Louise; University of Southampton, Global Health Research Institute, School of Human Development and Health, Faculty of Medicine; University of the Witwatersrand, School of Public Health, Faculty of Health Science<br>Padmadas, sabu; University of Southampton, Social Statistics and Demography
**Primary Subject Heading**:	Nutrition and metabolism
Secondary Subject Heading:	Global health
Keywords:	Nutrition < TROPICAL MEDICINE, Community child health < PAEDIATRICS, PUBLIC HEALTH

SCHOLARONE™
Manuscripts

BMJ

I, the Submitting Author has the right to grant and does grant on behalf of all authors of the Work (as defined in the below author licence), an exclusive licence and/or a non-exclusive licence for contributions from authors who are: i) UK Crown employees; ii) where BMJ has agreed a CC-BY licence shall apply, and/or iii) in accordance with the terms applicable for US Federal Government officers or employees acting as part of their official duties; on a worldwide, perpetual, irrevocable, royalty-free basis to BMJ Publishing Group Ltd ("BMJ") its licensees and where the relevant Journal is co-owned by BMJ to the co-owners of the Journal, to publish the Work in this journal and any other BMJ products and to exploit all rights, as set out in our licence.

The Submitting Author accepts and understands that any supply made under these terms is made by BMJ to the Submitting Author unless you are acting as an employee on behalf of your employer or a postgraduate student of an affiliated institution which is paying any applicable article publishing charge ("APC") for Open Access articles. Where the Submitting Author wishes to make the Work available on an Open Access basis (and intends to pay the relevant APC), the terms of reuse of such Open Access shall be governed by a Creative Commons licence – details of these licences and which Creative Commons licence will apply to this Work are set out in our licence referred to above.

Other than as permitted in any relevant BMJ Author's Self Archiving Policies, I confirm this Work has not been accepted for publication elsewhere, is not being considered for publication elsewhere and does not duplicate material already published. I confirm all authors consent to publication of this Work and authorise the granting of this licence.

For peer review only

Maternal socioeconomic status and infant feeding practices underlying pathways to child stunting in Cambodia: structural path analysis using cross-sectional population data

Chloe M. Harvey[1], Marie-Louise Newell[2,3] and Sabu S Padmadas[4]

[1]Independent Consultant, Bangkok, Thailand

[2]Global Health Research Institute, School of Human Development and Health, Faculty of Medicine, University of Southampton, Southampton, UK

[3]School of Public Health, Faculty of Health Science, University of the Witwatersrand, Johannesburg, South Africa

[4]Department of Social Statistics and Demography and Global Health Research Institute, University of Southampton, Southampton, UK

Corresponding author: Chloe M. Harvey, chloemercedes1991@gmail.com

Word count: 4563

For peer review only - http://bmjopen.bmj.com/site/about/guidelines.xhtml

ABSTRACT

Objectives: To identify and investigate complex pathways to stunting among children aged 6-24 months to determine the mediating effects of dietary diversity and continued breastfeeding on the association between socioeconomic factors and child stunting. Secondly, to assess whether these pathways are moderated by urban/rural residence.

Design, setting and participants: We analysed the most recent cross-sectional Demographic and Health Survey data from Cambodia (2014). We applied structural path analysis on a sample of 1,365 children, to model the complex and inter-related pathways of factors determining children's height-for-age. Explanatory variables included a composite indicator of maternal employment, household wealth, maternal education, current breastfeeding status and dietary diversity score. Results are presented both in terms of non-standardized and standardized coefficients. To examine the moderating effect of urban/rural residence on the proposed structural path models, both constrained and unconstrained solutions were tested.

Outcome measure: The primary outcome measure was height-for-age Z-scores (HAZ) as a continuous measure.

Results: Findings suggest that children's dietary diversity and continued breastfeeding mediate the association between socioeconomic status and children's height. Whilst there was no significant direct effect of maternal education on children's height, results suggested significant indirect pathways through which maternal education effects children's height; operating through household wealth, maternal employment, dietary diversity and continued breastfeeding status *(p<0.001)*. Most notably, 41% of the effect of maternal employment on children's height was mediated by either dietary diversity or continued breastfeeding. Furthermore, these associations differed between urban and rural areas, with results from simultaneous path analysis by urban-rural residence demonstrating that the strength of the associations between underlying socioeconomic factors and infant and young child feeding practices vary by urban-rural residence.

Conclusion: We provide evidence to support targeted nutrition interventions which account for the different ways in which poverty is experienced in urban and rural areas.

For peer review only - http://bmjopen.bmj.com/site/about/guidelines.xhtml

Article Summary

Strengths and limitations of this study

- Application of a causal hypothetical model to explain possible pathways to children's linear growth.
- Hypothetical models that can be applied to similar geographical contexts.
- The cross-sectional nature of DHS data, requires that results be interpreted with some caution.
- The theoretical model was built iteratively based on prior theory and thus, is not an exhaustive representation of the pathways to children's height.

For peer review only

For peer review only - http://bmjopen.bmj.com/site/about/guidelines.xhtml

INTRODUCTION

Across Southeast Asia, stunting in children under five years of age continues to pose a critical public health concern, with most countries in the region experiencing prevalence levels above 30%.[1] In Cambodia, more than a third of children under the age of five years are stunted;[1] stunting has been shown to be associated with distinct socioeconomic inequality, with sub-optimum feeding practices especially after 6 months of age, micronutrient deficiencies and recurrent infections.[2-3]

Impaired linear growth has adverse long-term health consequences for cognitive and immune system development, and is associated with increased risk of overweight and co-morbidities in later life.[2, 4, 5] Evidence shows that childhood stunting also has intergenerational implications, as stunting in pregnant women may lead to intrauterine growth restriction in her offspring,[6] thus continuing the vicious cycle of undernutrition. Interventions to tackle undernutrition in Southeast Asia have not been a top priority for governments and other stakeholders,[7] suggesting the need for strengthening further scientific evidence to understand the causal pathways of undernutrition, to identify appropriate strategies for policy and programme interventions.

Bio-behavioural and socioeconomic factors associated with children's height-for-age operate at varying levels of causation.[2] Limited dietary diversity has previously been reported to be significantly associated with impaired linear growth,[8] whereas the role of continued breastfeeding beyond six months remains inconclusive, with some studies even suggesting a negative association.[9, 10] However, it is not clear whether early feeding practices are directly and independently associated with height-for-age or whether they mediate the association between distal (underlying) factors and stunting.

Studies in Cambodia and other low- and middle-income countries have consistently shown maternal education, household wealth and maternal employment to be associated with inequalities in stunting prevalence.[2, 11-15] These three socioeconomic factors have also been associated with stark differentials in breastfeeding practices and dietary diversity within the Southeast Asian context.[16] However, associations underlying improved maternal socioeconomic status, continued breastfeeding and dietary diversity are paradoxical, as improvements in socioeconomic status (such as income, employment and education) are congruent with declines in breastfeeding, whereas improved socioeconomic status with increasing household income is positively associated with improved dietary diversity. Previous research has also indicated significant differentials in child nutritional status according to urban/rural residence,[2, 15] however, the intra-urban and intra-rural differentials are rarely considered, despite research suggesting starker socioeconomic inequalities within urban areas in low- and middle-income country settings.[17]

For peer review only - http://bmjopen.bmj.com/site/about/guidelines.xhtml

For peer review only

Most cross-sectional studies to date focusing on factors associated with linear growth have examined children's height-for-age as a dichotomous outcome, expressed as stunting. Evidence on the complex and inter-related nature of factors associated with a continuous measure of children's height-for-age is limited, including modelling techniques that account for dynamic interactions between possible explanatory variables, to disentangle direct effects from indirect effects with mediating variables.[18-20] To date, no such study has focussed on the Southeast Asian region, including Cambodia, where evidence-based interventions are critical for tackling the population burden of child undernutrition.

Using cross-sectional data from the most recent National Demographic and Health Survey (CDHS) in Cambodia, we identify and investigate theoretical pathways to stunting among children aged 6-24 months, when breastfeeding and dietary practices are most important for growth. We use structural path analysis to examine three hypotheses:(1) socioeconomic factors are directly associated with dietary diversity and continued breastfeeding;(2) dietary diversity and continued breastfeeding beyond six months of age mediate the association between socioeconomic factors and children's height-for-age, partially explaining the association between underlying socioeconomic factors and the outcome (children's height);(3) pathways are moderated by urban/rural residence (underlying socioeconomic factors have differing impacts on pathways to stunting, according to urban/rural residence).

METHODS

Data

We used the most recent Demographic and Health Survey (DHS) data from Cambodia,[21] which adopted a two-stage stratified sampling by separating rural and urban areas. The survey interviewed 17,578 women aged 15-49 years from 15,825 households, which included a sample of women from 5,667 urban and 11,911 rural areas. Anthropometric measurements, child health and health care data were collected for only those births that occurred five years preceding the survey. All eligible mothers were asked about the breastfeeding status of their children under 36 months at the time of survey visit; 24 hour-recall of additional liquids and solid foods was collected for the youngest child <36 months.

The sample of interest for this study was the youngest, living singleton child, between the ages of 6 and <24 months at the time of survey, living in the same household as their mother. As per CDHS

For peer review only - http://bmjopen.bmj.com/site/about/guidelines.xhtml

(2014) sampling design, anthropometric measurements were only taken in two-thirds of households, and after accounting for missing or implausible anthropometric information, a total of 1,365 Cambodian children between the ages of 6 and <24 months were selected (Supplementary Table S1). The children's file contains the records for every child born in the five years preceding the survey for women aged 15-49 years, who were selected randomly from the sampled households within each cluster.

The response rate for the women's survey from which information about the children is collected, was 98%. Additional information on the CHDS sampling design and survey methodology is reported elsewhere (CDHS, 2014). Item non-response for the selected explanatory variables was minimal, and the decision was made to exclude cases with missing data at random from the final analysis (<0.5% of total cases).

No additional data is available.

Dependent variable

Anthropometric data were collected by trained interviewers using internationally standard instruments. Height measurements were taken using a SECA measuring board.[21] For children aged less than 24 months, recumbent length measurements were taken and for older children, standing height was measured.[21]

Children's height-for-age Z-scores were calculated by the DHS using the WHO Child Growth Standards.[22] Stunting was defined as height-for-age Z-score below minus two standard deviations (-2 SD) from the mean of the reference population and severe stunting as below minus three standard deviations (-3 SD).[22] We used height-for-age Z-scores (HAZ) as a continuous measure, assuming that explanatory variables have varying effect on the respective Z-score distribution.

Explanatory variables

Selection of explanatory variables was based on previous research on the association between infant and young child feeding practices and child stunting, as well as two conceptual frameworks which depict the pathways through which different factors may affect child height/growth at different levels.[23, 24] Children's dietary diversity and breastfeeding at time of survey were selected as the two main proximate factors in the hypothetical structural path models. This was on the basis of evidence from the Cambodian DHS that suggests the main reasons for Cambodian children not meeting the minimum acceptable diet was due to not meeting the recommended minimum dietary diversity (consumption of four or more food groups in the previous 24 hours) but not due to meal

For peer review only - http://bmjopen.bmj.com/site/about/guidelines.xhtml

frequency.[21] Inclusion of continued breastfeeding status separately is important as while it is not included as a food group in the original WHO definition of minimum dietary diversity (MDD-7), it is recommended as an additional source of nutrients during the complementary feeding period.[25]

Terciles of dietary diversity were computed by age in months, based on the number of food groups consumed by children in the 24 hours preceding the survey,[26] to account for the gradual introduction of complementary foods.[27] Thus, children were regarded as having a low, middle or high diversity, relative to children of the same age. The definition of the terciles vary by age; Supplementary table S2 presents the distributions of the dietary diversity terciles by age.

Breastfeeding was categorised as a dichotomous variable as whether the child was being currently breastfed or not at child's age or time of survey.

The distal (underlying) factors included maternal education, household wealth and maternal employment. Maternal education reflected the highest level of completed education and was categorised into no education, primary, secondary and higher. Quintiles of household wealth were computed separately for urban and rural areas by DHS using principal component analysis, to ensure the representation of the poorest of the poor households, especially in rural areas.[28]

As children's nutritional status and feeding practices may be determined by a combination of different aspects and types of maternal employment,[29, 30] we created a composite indicator measuring maternal participation in the labour force based on an indicator computer by Sebayang, Efendi and Astutik.[31] Using principal component analysis, we incorporated five aspects of maternal employment: 1) working status over the past 12 months; 2) type of employment, whether self-employed or employed by family; 3) type of occupation; 4) type of earnings and 5) seasonality of work. Three distinctive categories of maternal employment were identified: not working, low level of participation, high level of participation, which was further dichotomised into a binary variable: (0) Not working/low participation; (1) High participation.

Statistical Analysis

Bivariate analyses included adjusted Wald tests for joint hypothesis testing to assess preliminary associations between explanatory variables and height-for-age Z-scores (Supplementary table S3). Statistical significance was considered at $p<0.05$ level.

Analysis was conducted using Stata/SE 15.1.[32] All descriptive and exploratory analyses accounted for the complex survey design of the CDHS using the appropriate sample weights.[21]

For peer review only - http://bmjopen.bmj.com/site/about/guidelines.xhtml

209 Structural path analysis is the structural component of structural equation modelling and involves the modelling of observed variables only.[33] Path analysis facilitates the study of direct and indirect effects of multiple independent and dependent variables simultaneously, based on a generalized multi-equation framework for examining multiple hypotheses about interdependencies between these variables.[34, 35] The first step of analysis involved creating a hypothetical "causal" pathway diagram between the independent variables of interest and the outcome HAZ (Figure 1). The hypothesised model was built iteratively based on prior theory and research, and therefore not an exhaustive representation of the pathways to children's height-for-age. It was selected as the appropriate methodology for this analysis as it allows for the identification of indirect and direct mechanisms through which socioeconomic factors influence height-for-age, where dietary diversity and breastfeeding may act as mediators in these associations.

220 Structural equation modelling assumes linearity of observed variables, and the assumption of linear normality amongst pseudo-continuous variables was tested with likelihood ratio (LR) tests and BIC/AIC diagnostics.[33] Most independent variables showed evidence of linearity, justifying their inclusion in the model as continuous variables. However, this was not the case for maternal employment as a continuous independent variable, and we thus used the dummy variable to adjust for high level of participation in employment, as this was the category of interest for this study.

226 To examine the moderating effect of urban/rural residence on the proposed structural path models, both constrained and unconstrained solutions were tested. The unconstrained solution where all parameters were allowed to vary by urban/rural residence provided a better fitting model and the results of this model are presented in this paper.

230

231 [Figure 1 Conceptual model of pathways between selected socioeconomic factors, dietary diversity, breastfeeding and height-for-age (HAZ) Z-scores (HAZ)]

233

234 **Assessment of model fit**

235 Structural path analysis was undertaken using the SEM program in Stata/SE 15.1. All models were estimated using maximum likelihood with missing values (MLMV). Goodness of fit of the data to the models were assessed using the root-mean-square error of approximation (RMSEA < 0.06) and the comparative fit index (CFI ≥ 0.95 for acceptance).[36] Both non-standardized and standardized coefficients are presented, but only standardized results are interpreted and presented on path diagrams (non-standardized coefficients use the raw/real life units of each variable and standardized

For peer review only - http://bmjopen.bmj.com/site/about/guidelines.xhtml

coefficients convert the coefficients into comparable units). Standardized coefficients are interpreted in terms of standard deviation differences.[34] To address the second hypothesis of this study that suggests dietary diversity and breastfeeding are mediators in the association between socioeconomic status and children's height-for-age Z-scores, standardized indirect effects were calculated.[33]

Finally, to test the moderating effect of urban-rural residence on path coefficients, as per the third hypothesis of this study, two approaches were taken. This part of the analysis involved comparing whether the associations presented in the hypothesised path model, varied by urban-rural residence and whether there were statistical differences for specific paths between urban-rural areas. Stata's SEM function facilitates the simultaneous analysis of the path model for different groups, to air comparison. The first approach involved an unconstrained model, where all parameters were permitted to vary by urban-rural residence and the second method involved a constrained solution in which all but one pathway was constrained to be the same. Results from both the unconstrained and constrained solution are presented.

Patient and public involvement

Secondary data was used for this analysis. Therefore, no patient consent was needed.

RESULTS

Sample characteristics

Approximately one-third of children were aged between 6-12 months and two-thirds were aged 12-23 months (Table 1), with 52% boys and 48% girls; 6% of children were born with a low birth weight (< 2.5kg). Just over two-fifth (44%) of children were reported to have had morbidity, either a fever, respiratory condition or diarrhoea, in the two weeks prior to interview. Five percent of mothers were of short stature (< 145cm); 16% had low (<18.5) and 15% high (≥25.0) BMI. Most mothers had completed primary level education (56%), and only 12% reported no formal education.
Approximately a third of women reported that they had not worked in the year prior to survey, a third of women had low level and the remainder high level of participation in employment. One in four of the children were from the poorest households and most households were located in rural areas (86%). About 70% of children aged 6-23 months were still being breastfed at the time of survey; 34% had been fed from a bottle. Approximately 43% of children had a low dietary diversity, but 73% of children consumed the recommended meal frequency.

For peer review only

Table 1 Summary characteristics of children 6-23 months from the Cambodian DHS (2014) (n=1,381*)

**Indicator**		**%**	**N**	**Mean HAZ**	**Std. deviation**	**95% CI for mean**	
						**Lower bound**	**Upper bound**
CHILD CHARACTERISTICS							
Age (months)	6-8	17.5	242	-0.55	1.25	-0.75	-0.35
	9-11	15.1	208	-0.92	1.48	-1.14	-0.7
	12-17	33.5	463	-1.41	1.23	-1.54	-1.28
	18-23	33.9	468	-1.46	1.32	-1.6	-1.31
Gender	Male	52.0	718	-1.28	1.34	-1.39	-1.16
	Female	48.0	663	-1.12	1.35	-1.24	-1
Birth weight	< 2.5kg	6.0	83	-1.48	1.5	-1.85	-1.11
	2.5 < 4.0kg	83.4	1152	-1.16	1.33	-1.25	-1.07
	≥ 4.0kg	3.4	46	-0.82	1.1	-1.17	-0.46
	Missing/not weighed	7.2	100	-1.63	1.38	-1.91	-1.36
Morbidity	No	56.5	781	-1.16	1.4	-1.27	-1.05
	Yes	43.5	601	-1.26	1.28	-1.39	-1.13
MATERNAL CHARACTERISTICS							
Height	Normal stature (≥145cm)	94.8	1308	-1.16	1.34	-1.25	-1.08
	Short stature (<145cm)	5.2	71	-1.9	1.36	-2.21	-1.59
BMI	<18.5	15.6	215	-1.5	1.22	-1.68	-1.32
	18.5-24.99	69.2	952	-1.17	1.39	-1.27	-1.06
	≥ 25.0	15.2	209	-1.09	1.24	-1.31	-0.86
Highest education level	No education	12.0	166	-1.26	1.45	-1.5	-1.02
	Primary	55.6	768	-1.28	1.27	-1.4	-1.16
	Secondary/Higher	32.4	447	-1.04	1.42	-1.18	-0.91
Maternal employment	Not working	30.5	421	-1.19	1.22	-1.34	-1.04
	Low	37.5	517	-1.4	1.42	-1.54	-1.26
	High	32.0	441	-0.98	1.34	-1.12	-0.84
HOUSEHOLD CHARACTERISTICS							
Household Wealth Index	Poorest	25.4	345	-1.48	1.49	-1.65	-1.32
	Poorer	18.2	248	-1.32	1.41	-1.5	-1.14
	Middle	19.9	271	-1.23	1.18	-1.41	-1.05
	Richer	17.7	241	-1	1.21	-1.2	-0.8
	Richest	18.8	256	-0.86	1.17	-1.04	-0.67
Urban/rural residence	Rural	86.4	1193	-1.25	1.23	-1.34	-1.16
	Urban	13.6	188	-0.88	1.97	-1.06	-0.7

For peer review only

For peer review only - http://bmjopen.bmj.com/site/about/guidelines.xhtml

Table 1 contd. Summary characteristics of children 6-23 months from the Cambodian DHS (2014) (n=1,381*)

Indicator		%	N	Mean HAZ	Std. deviation	95% CI for mean	
						Lower bound	Upper bound
Sex of household head	Male	77.0	1064	-1.2	1.33	-1.29	-1.1
	Female	23.0	317	-1.21	1.39	-1.4	-1.02
IYCF CHARACTERISTICS							
Breastfeeding	No	29.9	413	-1.15	1.39	-1.3	-0.99
	Yes	70.1	968	-1.22	1.33	-1.32	-1.12
Bottle feeding	No	66.0	911	-1.27	1.33	-1.38	-1.16
	Yes	34.0	469	-1.07	1.37	-1.2	-0.93
Dietary Diversity Tercile	Low	43.2	596	-1.3	1.2	-1.42	-1.17
	Middle	33.8	467	-1.29	1.46	-1.44	-1.13
	High	23.1	318	-0.9	1.41	-1.07	-0.73
Minimum meal frequency	No	27.4	378	-1.32	1.38	-1.48	-1.16
	Yes	72.6	1003	-1.16	1.33	-1.25	-1.06
Minimum acceptable diet	No	68.4	945	-1.23	1.32	-1.33	-1.13
	Yes	31.6	436	-1.13	1.41	-1.29	-0.98

* Sample size differs slightly from the sample used in the final models (*n*=1,365) due to missing data for some children on explanatory variables.

For peer review only

Results of structural path analysis

Underlying socioeconomic factors

Figure 2 represents the full structural path model; the standardized and unstandardized coefficients are presented in Supplementary Table S4. Overall, the fit of the model according to RMSEA (< 0.05) and CFI (>0.950) was good, and the model explained approximately 44% of the variance in HAZ scores (R^2=0.44).

Maternal education was significantly and positively associated with dietary diversity. One SD increase in the level of maternal education was associated with a 0.07 SD increase in dietary diversity (β = 0.07; p <0.05), but with no significant effect on breastfeeding status. Similarly, a one SD increase in household wealth was associated with a 0.18 SD increase in dietary diversity tercile (β = 0.18; p<0.001), but a one SD increase in household wealth was associated with -0.09 SD decrease in breastfeeding. A high level of maternal employment was associated with a positive, but

For peer review only - http://bmjopen.bmj.com/site/about/guidelines.xhtml

insignificant effect on dietary diversity tercile, but statistically significantly negatively associated with breastfeeding status (β=-0.07; $p<0.01$). Further analysis revealed that this effect on breastfeeding status was confirmed amongst younger age groups, but not in the 18-23 month age group (Supplementary table S5).

[Figure 2 Full structural path model, measuring how socioeconomic factors are associated with children's HAZ Z-scores directly and through mediators of dietary diversity and continued breastfeeding. Standardized coefficients. Cambodia (2014)]

Dietary diversity and continued breastfeeding beyond 6 months of age as mediating factors

Both hypothesised mediating variables – dietary diversity tercile and breastfeeding status – were independently and significantly associated with child HAZ score, although associations were in the opposite direction. One SD increase in dietary diversity was associated with a 0.06 SD increase in HAZ ($p<0.05$), whereas continued breastfeeding was associated with a -0.13 decrease in HAZ ($p<0.001$) (Figure 2 and Supplementary table S4). The only socioeconomic factor with a significant independent association with HAZ was household wealth, where a 1 SD increase in household wealth was associated with a 0.06 SD increase in HAZ ($p<0.05$).

To assess the role of dietary diversity and breastfeeding as mediators in the association between socioeconomic factors (maternal education, household wealth, maternal employment (high)) and HAZ, indirect pathways were examined (Table 2). In terms of maternal education, the standardized effects presented a case of 'inconsistent mediation',[37] where the direct and indirect effects do not have the same consistent sign. Furthermore, whilst the indirect effect is significant ($p<0.001$), the direct effect is not and hence, the indirect effect is larger than the total effect meaning that the effect of maternal education on HAZ is fully attributed to the indirect path which operates through household wealth, maternal employment, dietary diversity and breastfeeding status.

For peer review only - http://bmjopen.bmj.com/site/about/guidelines.xhtml

Table 2 Standardized coefficients of maternal education, household wealth, maternal employment and child height-for-age Z-score, with correlated residuals for dietary diversity and breastfeeding status

**Outcome**	**Direct effect (β)**	**Indirect effect (β)**	**Total effect**
HAZ			
Dietary diversity -> HAZ	0.061*	na	0.061*
Breastfeeding -> HAZ	-0.129***	-0.007	-0.136***
Maternal Education -> HAZ	-0.008	0.051***	0.043
Maternal employment (high) -> HAZ	0.054	0.039***	0.094**
Household wealth -> HAZ	0.057*	0.022**	0.080**

The significance levels are for the unstandardized solution.

*p-value<0.05, **p-value<0.01, ***p-value<0.001, na: not applicable

Although there was no direct effect of maternal employment on HAZ, statistically significant indirect effects suggested that 41% (0.039/0.094=0.41) of the effect of maternal employment on HAZ was mediated by either dietary diversity or breastfeeding status.

Household wealth was the only underlying (socioeconomic) factor with a statistically significant direct effect on HAZ, with 71% of the total effect on HAZ estimated to be direct (0.057/0.080=0.71), after controlling for dietary diversity and breastfeeding status, with the indirect effect of household wealth on HAZ an estimated 28% (0.022/0.080=0.28).

Further exploration of the specific indirect pathways between underlying (socioeconomic) factors and HAZ, through mediating variables of dietary diversity and breastfeeding, showed evidence of three indirect pathways that were statistically significant (Supplementary Table S6). High level of maternal employment had a small but significant standardized effect on HAZ, mediated by breastfeeding status of 0.01, z=2.30, p <0.05. Household wealth also had a small but statistically significant standardized effect on HAZ, mediated by both dietary diversity and breastfeeding status (0.01, z=2.13, p<0.05; 0.01, z=2.58, p<0.01 respectively).

For peer review only - http://bmjopen.bmj.com/site/about/guidelines.xhtml

Moderating effect of urban/rural residence

Exploratory analysis suggested differences in mean height-for-age Z-scores between urban and rural areas, with children in rural areas in Cambodia generally fairing worse (Supplementary figure S1). Results from an unconstrained solution (Table 3), which accounted for the moderating effects of urban/rural residence by allowing all parameters to vary, suggested that urban-rural residence had a significant moderating effect on some of the pathways in the hypothesised path analysis. Firstly, a 1 SD increase in maternal education was associated with a 0.07 SD increase in dietary diversity in rural areas ($p<0.05$), which was also evident in urban areas but this association was not significant. In terms of the underlying (socioeconomic) factors associated with breastfeeding, a high level of maternal employment was significantly negatively associated with breastfeeding status in rural areas, where a 1 SD increase in maternal employment was associated with a -0.07 SD decrease in breastfeeding ($p<0.05$), although this same association was insignificant in urban areas.

In urban areas, a 1 SD increase in household wealth was associated with a statistically significant decrease in breastfeeding by -0.26 SD (*$p<0.01$),* whereas there was a smaller, insignificant association observed in rural areas, suggesting a strong moderating effect of urban-rural residence on this pathway. Finally, the results suggested that both household wealth and dietary diversity were positively associated with HAZ through a statistically significant direct pathway, a finding which was not observed in urban areas (0.10, $p<0.01$; 0.07, $p<0.05$ respectively).

For peer review only - http://bmjopen.bmj.com/site/about/guidelines.xhtml

Table 3 Summary table for multiple-group analysis, unstandardized (B) and standardized coefficients (β)

Path	Unconstrained solution			
	Rural (n=1001)		Urban (n=364)	
	B	β	B	β
Maternal employment (high)				
Maternal education	0.157***	0.231***	0.197***	0.245***
Household wealth				
Maternal employment (high)	1.091***	0.325***	0.780***	0.277***
Maternal education	0.711***	0.312***	0.686***	0.305***
Dietary diversity				
Maternal employment (high)	0.057	0.032	0.161	0.101
Household wealth	0.084***	0.159***	0.128***	0.225***
Breastfeeding	-0.128*	-0.072*	-0.284**	-0.178**
Maternal education	0.087*	0.072*	0.070	0.054
Breastfeeding				
Maternal employment (high)	-0.072*	-0.072*	-0.065	-0.065
Household wealth	-0.007	-0.024	-0.093***	-0.262***
Maternal education	-0.010	-0.015	0.044	0.054
HAZ				
Maternal employment (high)	0.116	0.036	0.054	0.017
Household wealth	0.095**	0.099**	-0.004	-0.004
Dietary diversity	0.119*	0.066*	0.031	0.015
Breastfeeding	-0.370**	-0.115**	-0.549**	-0.168**
Maternal education	-0.135	-0.062	0.196	0.075
**χ^2 overall (df)**	81.69(40)			
**RMSEA**	0.039			
**CFI**	0.962			

*p-value<0.05, **p-value<0.01, ***p-value<0.001. **Bold:** Unconstrained parameter in the constrained model.

For peer review only

DISCUSSION

The current study extends previous research examining factors associated with child stunting, to present a robust statistical model, which reflects the complex interactions between variables associated with children's height-for-age. Structural path analysis allows for the replication of a theoretical framework with underlying and proximate factors, to examine both the indirect and direct effect of factors known to be associated with child stunting. The structural path model presented could be applicable to other low- and middle-income country contexts and could examine whether our proposed pathways to stunting are consistent across geographical contexts.

Overall, the results of this study confirmed that improvements in socioeconomic status such as maternal education, household wealth and participation in employment, were associated with an increase in children's dietary diversity, but a decrease in continued breastfeeding beyond 6 months of age. The findings also suggested that dietary diversity and continued breastfeeding mediate the associations between socioeconomic status and height-for-age Z-scores. High level of maternal employment had a small but significant association with HAZ that was mediated by breastfeeding status, while the significant association between household wealth and HAZ was mediated by both dietary diversity and breastfeeding status. This suggests that interventions to improve child nutritional status should make due consideration for the underlying socioeconomic drivers which may influence infant and young child feeding practices. Women's employment conditions is one such factor that has been found to influence infant feeding practices globally, especially early weaning among mothers returning to work. [38] Although the female labour force participation rate is relatively high in Cambodia, with 74% of women participating in the labour force, [39] Cambodia has one of the highest proportions of informally employed (93.1 % among men and women), [40] which is often associated with a lack of access to maternity protection. [41]

The independent effect of maternal education on dietary diversity was small but significant, consistent with the findings of other studies conducted in low- and middle-income countries,[42-45] in addition to studies which confirmed a strong association between increasing household wealth and improvements in dietary diversity.[15, 46] The significant pathway between breastfeeding and dietary diversity demonstrated an additional, interesting observation, that continued breastfeeding beyond 6 months of age, was associated with a less diverse diet, even after adjusting for age. This could be indicative of the situation in poorer households where prolonged breastfeeding may be practiced to make up for the lack of complementary foods, as suggested from a study of feeding practices in Thailand.[9]

For peer review only - http://bmjopen.bmj.com/site/about/guidelines.xhtml

In light of the nutrition transition, which has been occurring in many low- and middle-income countries, Popkin and Bisgrove[47] reported that duration of breastfeeding was declining in urban areas as a result of the increased maternal education and employment opportunities that arise from urbanisation. Furthermore, the negative association between increasing household wealth and continued breastfeeding is perhaps underscored by the increasing trend in use of breast milk substitutes among children aged over six months of age in Cambodia.[48] Although the Cambodian government adopted many aspects of the *International Code of Marketing of Breast-milk Substitutes* [49] in the Cambodian *Sub-Decree on Marketing of Products for Infant and Young Child Feeding (Sub-Decree 133),* [50] a recent pilot implementation of a monitoring and enforcement system found significant non-compliance with this Sub-Decree at point-of-sales and in health facilities. [51]

Our study confirmed that dietary diversity and continued breastfeeding mediate associations between socioeconomic status and child stunting, however these findings were only significant for pathways originating from maternal employment and household wealth. The results of this study suggested a positive and direct association between Cambodian children's dietary diversity and height-for-age z-scores, which has also been supported by other studies conducted in Cambodia.[52, 53] Moreover, dietary diversity was a significant mediator of the association between household wealth and HAZ suggesting that the positive effect of increasing household wealth can partially be explained by improved dietary diversity. This highlights the need to prioritise targeted interventions within the poorest households to improve dietary diversity to prevent child stunting. A further 71% of the total effect of household wealth on HAZ was found to be direct, suggesting the importance of other mediating factors not included in this model but known to be associated with child stunting, such as access to health care, WASH facilities and immunization status.

On the other hand, breastfeeding after age 6 months was associated with a negative, direct effect on children's HAZ in Cambodia in the path model that adjusted for age of the child. This was also noted by Arimond and Ruel,[27] in a study using Cambodian DHS data from 2000, and in a review of 13 studies by Grummer-Strawn.[54] A negative effect of breastfeeding beyond age six months on children's height-for-age status has been discussed by many researchers. One proposed explanation is reverse causality, whereby the decision to wean is related to mother's perceptions of size and stature of the child, hence malnourished children may be more likely to be breastfed for longer durations.[55] However, as Martin[56] points out, due to the cross-sectional nature of structural path analysis, it is not possible to account for the direction in the relationship between prolonged breastfeeding and HAZ. In terms of the indirect effects, structural path analysis revealed that

For peer review only

breastfeeding was a small but significant mediator in the association between maternal employment and HAZ, as well as household wealth and HAZ.

Finally, this study highlighted the moderating effect of urban-rural residence, in particular, the differences in individual factors that are associated with specific pathways to HAZ, but also that the strength of these associations varies by urban-rural residence. This analysis shed new light on the differences in pathways to stunting by urban-rural residence in Cambodia, and so far is one of the first studies which has separately analysed the effect of urban-rural residence. Results from simultaneous path analysis by urban-rural residence demonstrated the moderating effect of this variable, stressing the importance of designing nutritional interventions which account for the differences in the determinants of stunting between urban and rural areas. This is especially important for low- and middle-income contexts such as Cambodia, where there are distinct urban-rural differentials in the incidence of stunting and where rapid urbanisation is causing a shift in the geographical patterning of stunting.

Moreover, as shown by an unconstrained model where all parameters were permitted to vary by urban-rural residence, maternal education was a significant and positive predictor of dietary diversity in rural areas, but not in urban areas. Although this difference between rural and urban areas was not statistically significant, it may provide evidence in support of community-based interventions that target nutrition education programmes amongst rural mothers. For example, a successful FAO-supported agricultural intervention known as MALIS (Improving market linkages for smallholder farmers) combined a nutritional education programme focused on infant and young child feeding (IYCF) practices in rural Cambodia, resulting in a significant increase in child dietary diversity, specifically increased consumption of vitamin A-rich foods and other fruits and vegetables[57]

Although increasing household wealth was significantly negatively associated with breastfeeding status in the full structural path model, further analysis controlling for the moderating effect of urban-rural residence revealed a statistically significant difference in the effect of household wealth on breastfeeding status between urban and rural areas. Household wealth was significantly and negatively associated with breastfeeding status in urban areas in both an unconstrained and constrained solution, which controlled for this moderating effect, suggesting extreme intra-urban inequality in breastfeeding practices between the urban-poor and the urban-rich. This finding emphasises the fact that within urban areas interventions to improve breastfeeding practices should be mindful of the socioeconomic disparities in practices that occur even within urban spaces.

For peer review only

STRENGTHS AND LIMITATIONS

This study used population-based data from the most recent nationally representative household survey from Cambodia, and our findings are generalizable to Cambodia. DHS data uses standard instruments to measure height by professionally trained investigators, which ensures accuracy of anthropometric measurements, however, the way in which continued breastfeeding and dietary diversity are reported using current-status data (from a 24 hour recall questionnaire) may introduce measurement bias, although this would apply equally to stunted and non-stunted children. Although the 2014 Cambodian DHS is the latest available household survey data for Cambodia, findings should be interpreted with caution when referring to the current context as the data is eight years old. Application of a causal hypothetical model to explain possible pathways to children's linear growth, presents the first study of its kind using Cambodian DHS data, and lays the foundation for similar studies in other geographical contexts. However, the cross-sectional nature of DHS data and the use of structural path analysis, typically a form of causal modelling, requires that the results be interpreted with some caution. Although structural path models may offer benefits over multivariable fixed effects models in allowing for complex interactions between variables, the theoretical model was built iteratively based on prior theory and thus, is by no means an exhaustive representation of the pathways to children's HAZ scores.

CONCLUSIONS

The theoretical structural path model presented in this study proposes a plausible illustration of the pathways associated with children's height-for-age in the Cambodian context. Overall, results confirmed that children's dietary diversity and continued breastfeeding status mediate the associations between socioeconomic status and linear growth, at least for pathways from maternal employment and household wealth. Analysis suggests that participation in maternal employment presents a significant barrier to continued breastfeeding beyond age six months of age, emphasising a need for maternity legislation that supports optimal breastfeeding practices, in line with WHO recommendations that infants be exclusively breastfed for six months, followed by continued breastfeeding in addition to complementary feeding up to two years of age.[25] Improvements to maternity legislation should also be inclusive of women working in the informal sector (of which 96% of employed women in Cambodia participate). Further simultaneous path analysis of the proposed model highlighted the moderating effect of urban-rural residence, emphasizing the need for

For peer review only - http://bmjopen.bmj.com/site/about/guidelines.xhtml

nutrition interventions which not only target women and children of low socioeconomic status, but which also account for the different ways in which poverty is experienced in urban and rural areas.

CONTRIBUTORS

CMH conceptualised the study, prepared the dataset for research and conducted statistical analysis under the supervision of MLN and SSP. MLN and SSP contributed to the interpretation and revised paper for intellectual content. CMH, MLN and SPP conducted the final review and revised the manuscript for submission.

For peer review only

COMPETING INTERESTS

None declared.

FUNDING

This research was carried out as a part of a doctoral research project, supported by the UK Economic and Social Research Council (grant number ES/J500161/1). The Economic and Social Research Council had no role in the design, analysis or writing of this article.

DATA SHARING

No additional data available

ETHICS STATEMENT

This study is based on publicly available DHS data. CMH was granted access to the data by the MEASURE DHS/ICF International, Rockville, Maryland, USA.

For peer review only - http://bmjopen.bmj.com/site/about/guidelines.xhtml

REFERENCES

1. [dataset] United Nations Children's Fund (UNICEF). Data from: Stunting (Survey Estimates, National and Disaggregated). April 11, 2021. https://data.unicef.org/topic/nutrition/malnutrition/
2. Black RE, Victora CG, Walker SP, et al. Maternal and child undernutrition and overweight in low-income and middle-income countries. *The Lancet* 2013;382:427–451.
3. Dewey KG, Mayers DR. Early child growth: How do nutrition and infection interact? *Matern Child Nutr* 2011;*7*(Suppl 3):129–142.
4. Black RE, Allen LH, Bhutta ZA, et al. Maternal and child undernutrition: global and regional exposures and health consequences. *The Lancet* 2008;371:243–260
5. Victora CG, Adair L, Fall C, et al. Maternal and child undernutrition: consequences for adult health and human capital. *The Lancet* 2008;371:340–357.
6. Ozaltin E, Hill K, Subramanian SV. Association of maternal stature with offspring mortality, underweight, and stunting in low- to middle-income countries. *JAMA*, 2010;303:1507-1516.
7. Mutunga M, Frison S, Rava M, et al. The Forgotten Agenda of Wasting in Southeast Asia: Burden, Determinants and Overlap with Stunting: A Review of Nationally Representative Cross-Sectional Demographic and Health Surveys in Six Countries. *Nutrients* 2020;12:559.
8. Krasevec J, An X, Kumapley, R, et al. Diet quality and risk of stunting among infants and young children in low- and middle-income countries. *Matern Child Nutr* 2017;13(Suppl 2):1–11.
9. Cetthakrikul N, Topothai C, Suphanchaimat R, et al. Childhood stunting in Thailand: When prolonged breastfeeding interacts with household poverty. *BMC Pediatr* 2018;18: 1–9
10. Marquis GS, Habicht JP, Lanata CF, et al. Association of breastfeeding and stunting in Peruvian toddlers: An example of reverse causality. *Int J Epidemiol* 1997;26:349–356.
11. Boyle M, Georgiades K, Racine Y, et al. The influence of economic development level, household wealth and maternal education on child health in the developing world. *Soc Sci Med* 2006;63:2242–2254.
12. Caldwell JC. Education as a Factor in Mortality Decline An Examination of Nigerian Data. *Popul Stud (NY)* 1979;33:395–413.
13. Caldwell JC. How is greater maternal education translated into lower child mortality? *Health Transit Rev* 1994;4:224–229.

For peer review only - http://bmjopen.bmj.com/site/about/guidelines.xhtml

14. Popkin BM, Solon FS. Income, time, the working mother and child nutriture. *J Trop Pediatr* 1976;22:156–166.
15. Smith LC, Ruel MT, Ndiaye A. Why is child malnutrition lower in urban than in rural areas? Evidence from 36 developing countries. *World Dev* 2005;33:1285–1305.
16. Harvey CM, Newell ML, Padmadas SS. Socio-economic differentials in minimum dietary diversity among young children in minimum dietary diversity among young children in South-East Asia: evidence from Demographic and Health Surveys. *Public Health Nutr* 2018;21:3048-3057.
17. Menon P, Ruel MT, Morris SS. Socio-economic differentials in child stunting are consistently larger in urban than rural areas. *Food Nutr Bull* 2000;21:282-289.
18. Hanieh S, Ha TT, De Livera AM, et al. Antenatal and early infant predictors of postnatal growth in rural Vietnam: A prospective cohort study. *Arch Dis Child* 2015;100:165–173.
19. Lander RL, Williams SM, Costa-Ribeiro H, et al. Understanding the complex determinants of height and adiposity in disadvantaged daycare preschoolers in Salvador, NE Brazil through structural equation modelling. *BMC Public Health* 2015;15:1–8.
20. Cunningham K, Ferguson E, Ruel M, et al. Water, sanitation, and hygiene practices mediate the association between women's empowerment and child length-for-age z-scores in Nepal. *Matern Child Nutr* 2018;15:1–9.
21. [dataset] National Institute of Statistics, Directorate General for Health, & ICF International. Data from: Cambodia Demographic and Health Survey 2014. The DHS Program, August 17, 2018. https://dhsprogram.com/methodology/survey/survey-display-464.cfm
22. World Health Organization (WHO). *WHO Multicentre Growth Reference Study Group. WHO Child Growth Standards: Length/height-for-age, weight-for-age, weight-for-length, weight-for-height and body mass index-for-age: Methods and development.* Geneva: WHO, 2006. https://www.who.int/publications/i/item/924154693X
23. Stewart CP, Iannotti L, Dewey KG, et al. Contextualising complementary feeding in a broader framework for stunting prevention. *Matern Child Nutr* 2013;9(Suppl 2): 27–45.
24. Wamani H, Åstrøm AN, Peterson S, et al. Infant and young child feeding in Western Uganda: Knowledge, practices and socio-economic correlates. *J Trop Pediatr* 2005;51:356–361.
25. World Health Organization (WHO) Continued breastfeeding for healthy growth and development of children [Internet]. WHO, 2017. Available: https://www.who.int/elena/titles/bbc/continued_breastfeeding/en/ [Accessed 02 Sept 2019].

For peer review only - http://bmjopen.bmj.com/site/about/guidelines.xhtml

26. World Health Organization (WHO). *Indicators for assessing infant and young child feeding practices, Part 1: Definitions*. Geneva: WHO, 2008. https://apps.who.int/iris/bitstream/handle/10665/43895/9789241596664_eng.pdf;jsessionid=42E6F2DE11CA32FA51E4636A4301253A?sequence=1
27. Arimond M, Ruel MT. Dietary diversity is associated with child nutritional status: evidence from 11 demographic and health surveys. *J Nutr* 2004;*134:*2579–2585.
28. Rutstein SO. The DHS Wealth Index. DHS Working Paper. *USAID* 2008;60 [Working Paper] https://www.researchgate.net/publication/238706094_The_DHS_Wealth_Index_Approaches_for_Rural_and_Urban_Areas
29. Nankinga O, Kwagala B, Walakira EJ. Maternal employment and child nutrition status in Uganda. *PloS One* 2019;14(e0226720). https://journals.plos.org/plosone/article?id=10.1371/journal.pone.0226720 (accessed 5 August 2020).
30. Debela BL, Gehrke E, Qaim M. Links between Maternal Employment and Child Nutrition in Rural Tanzania. *Am J Agric Econ* 2020;103:812-830.
31. Sebayang SK, Efendi F, Astutik E. Women's empowerment and the use of antenatal care services: analysis of demographic health surveys in five Southeast Asian countries. *Women Health* 2017;59:1-17.
32. [software] StataCorp. *Stata Statistical Software: Release 15*. StataCorp LLC 2017.
33. Acock A. Discovering Structural Equation Modeling Using Stata. Texas, US: Stata Press 2013.
34. Grace J, Bollen K. Interpreting the Results from Multiple Regression and Structural Equation Models. *Bulletin Ecological Society of America* 2005;86:283–295.
35. Hox JJ, Bechger T. An Introduction to Structural Equation Modeling. *Family Science Review* 1999;11:354–373.
36. Schreiber JB, Nora A, Stage FK. Reporting Structural Equation Modelling and Confirmatory Factor Analysis Results: A Review. *J Educ Res* 2006;99:323–338.
37. Mackinnon DP, Fairchild AJ, Fritz MS. Mediation Analysis. *Annu Rev Psychol* 2007 ;58 :1–22.
38. Rollins NC, Bhandari N, Hajeebhoy N, et al. Why invest, and what it will take to improve breastfeeding practices? *Lancet* 2016;387:491-504.
39. ILOSTAT. Labour force participation rate, female – Cambodia. 2021. Available : https://data.worldbank.org/indicator/SL.TLF.CACT.FE.ZS?locations=KH (accessed 5 August 2020)
40. International Labour Organization (ILO). *Women and men in the informal economy: a statistical picture (third edition).* Geneva: ILO, 2018.

For peer review only - http://bmjopen.bmj.com/site/about/guidelines.xhtml

https://www.ilo.org/wcmsp5/groups/public/---dgreports/---dcomm/documents/publication/wcms_626831.pdf

41. Addati L, Cassirer N, Gilchrist K. *Maternity and paternity at work: Law and practice across the world.* Geneva: ILO, 2014. https://www.ilo.org/wcmsp5/groups/public/---dgreports/---dcomm/---publ/documents/publication/wcms_242615.pdf
42. Na M, Aguayo VM, Arimond M, et al. Trends and predictors of appropriate complementary feeding practices in Nepal: An analysis of national household survey data collected between 2001 and 2014. *Matern Child Nutr* 2018;14(Suppl 4):1–13.
43. Patel A, Pusdekar Y, Badhoniya N, et al. Determinants of inappropriate complementary feeding practices in young children in India: secondary analysis of National Family Health Survey 2005 – 2006. *Matern Child Nutr* 2012;8(Suppl 1):28–44.
44. Kabir I, Khanam M, Agho KE, et al. Determinants of inappropriate complementary feeding practices in infant and young children in Bangladesh: secondary data analysis of Demographic Health Survey 2007. *Matern Child Nutr* 2012;8(Suppl 1):11–27.
45. Senarath U, Agho KE, Akram DES, et al. Comparisons of complementary feeding indicators and associated factors in children aged 6-23 months across five South Asian countries. *Matern Child Nutr* 2012;8(Suppl 1):89–106.
46. Iannotti LL, Robles M, Pachón H, et al. Food Prices and Poverty Negatively Affect Micronutrient Intakes in Guatemala. *J Nutr* 2012;142:1568–1576.
47. Popkin BM, Bisgrove EZ. Nutrition and Urbanization (Part 2): Urbanization and Nutrition in low-Income countries. *Food Nutr Bull* 1988;10:1–22.
48. Prak S, Dahl MI, Oeurn S, et al. Breastfeeding Trends in Cambodia, and the Increased Use of Breast-Milk Substitute—Why Is It a Danger? *Nutrients* 2014;6:2920–2930.
49. World Health Organization (WHO). *International Code of Marketing of Breastmilk Substitutes.* Geneva: WHO, 1981. https://apps.who.int/iris/handle/10665/40382
50. Royal Government of Cambodia. *Sub-Decree on Marketing of Products for Infant and Young Child Feeding, No. 133, A Nor Kra Bor.* Phnom Penh: Royal Government of Cambodia, 2005.
51. Hou L, Green M, Chum S, et al. Pilot implementation of monitoring and enforcement system for the International Code of Marketing of Breast-milk Substitutes in Cambodia. *Maternal and Child Nutrition* 2019; e12795.
52. Greffeuille V, Sophonneary P, Laillou A, et al. Persistent inequalities in child undernutrition in Cambodia from 2000 until today. *Nutrients* 2016;8:1–17.
53. Darapheak C, Takano T, Kizuki M, et al. Consumption of animal source foods and dietary diversity reduce stunting in children in Cambodia. *Int Arch Med* 2013;6:1–11.

For peer review only

For peer review only - http://bmjopen.bmj.com/site/about/guidelines.xhtml

54. Grummer-Strawn L. Regression analysis of current-status data: an application to breast-feeding. *J Am Stat Assoc* 1993;88:758–765.

55. Simondon K, Costes R, Delaunay V, et al. Children's height, health and appetite influence mothers' weaning decisions in rural Senegal. *Int J Epidemiol* 2001;30:476–481.

56. Martin RM. Commentary: Does breastfeeding for longer cause children to be shorter? *Int J Epidemiol* 2001;30:481–484.

57. Reinbott A, Schelling A, Kuchenbecker J, et al. Nutrition education linked to agricultural interventions improved child dietary diversity in rural Cambodia. *Br J Nutr* 2016;116:1457-1468.

For peer review only

For peer review only - http://bmjopen.bmj.com/site/about/guidelines.xhtml

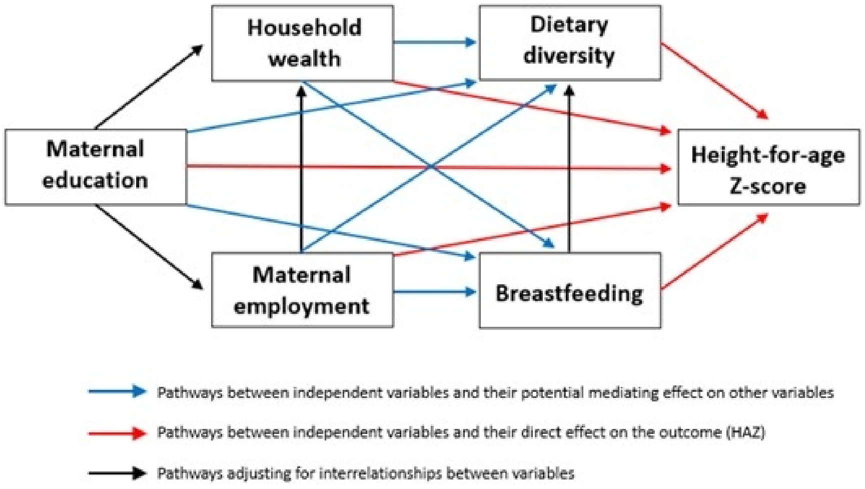

Figure 1 Conceptual model of pathways between selected socioeconomic factors, dietary diversity, breastfeeding and height-for-age (HAZ) Z-scores (HAZ)

162x107mm (96 x 96 DPI)

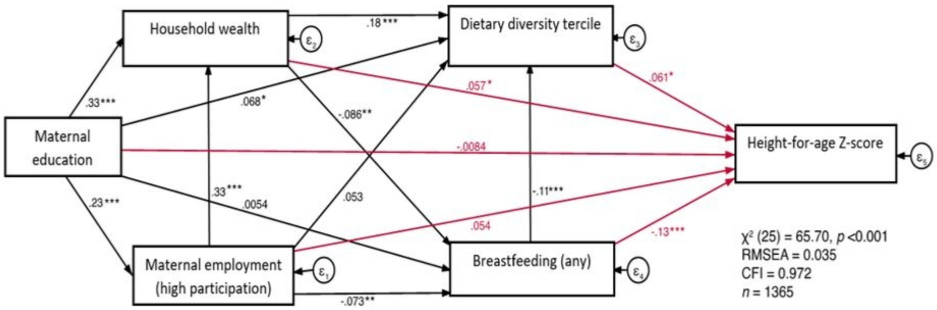

*p-value<0.05, **p-value<0.01, ***p-value<0.001

Note: Model is adjusted for age, sex, birth weight, maternal stature and urban/rural residence

RMSEA (<0.05) and CFI (>0.95) indicate an overall good fit of the model

Figure 2 Full structural path model, measuring how socioeconomic factors are associated with children's HAZ Z-scores directly and through mediators of dietary diversity and continued breastfeeding. Standardized coefficients. Cambodia (2014)

259x137mm (96 x 96 DPI)

Supplementary Table S1. Sample size selection: children aged 6 to <24 months with plausible anthropometric measurements, Cambodia (2014)

	n
Children 6-23.9 months	2,127
Children 6-23.9 months with anthropometric measurements	1,397
**Children 6-23.9 months with plausible† anthropometric measurements**	**1,365**

† Plausible HAZ scores should fall between -6 and +6 standard deviations, according to the WHO SD flag limits (WHO, 2006)

Supplementary Table S2. Distribution of dietary diversity tercile by child's age, adjusted estimates, Cambodia (2014)

	**Age in months (*n* = 1,381)**								
	**6-8 (*n*=242)**		**9-11 (*n*=208)**		**12-17 (*n*=463)**		**18-23 (*n*=468)**		**6-23 (*n*=1,381)**
**Dietary Diversity Tercile**	%	**DDS range**	**%**	**DDS range**	**%**	**DDS range**	**%**	**DDS range**	**%**
Low	44.9	0-1 groups	41.0	0-2 groups	49.5	0-3 groups	37.0	0-3 groups	**43.2**
Middle	17.9	2 groups	43.0	3-4 groups	26.2	4 groups	45.4	4-5 groups	**33.8**
High	37.2	≥ 3 groups	16.0	≥ 5 groups	24.4	≥ 5 groups	17.6	≥ 6 groups	**23.1**

For peer review only

Supplementary Table S3. Association between IYCF indicators, socio-demographic factors and mean height-for-age Z-scores in children aged 6-23 months in Cambodia (2014), (estimates adjusted for survey design using appropriate sample weights)

**Indicator**	**%**	**N**	**Mean HAZ**	**Std. deviation**	**95% CI for mean**	**Test statistic**
Inherent (biological) factors						
Age (months)						
6-8	17.5	242	-0.55	1.25	-0.75, -0.35	F(3, 1362)=22.20, *p*=<0.001
9-11	15.1	208	-0.92	1.48	-1.14, -0.70	
12-17	33.5	463	-1.41	1.23	-1.54, -1.28	
18-23	33.9	468	-1.46	1.32	-1.60, -1.31	
Gender						
Male	52.0	718	-1.28	1.34	-1.39, -1.16	F(1, 1364)=3.33, *p*=0.068
Female	48.0	663	-1.12	1.35	-1.24, -1.00	
Birth weight						
< 2.5kg	6.0	83	-1.48	1.50	-1.85, -1.11	F(3, 1362)=5.65, *p*=<0.001
2.5 < 4.0kg	83.4	1152	-1.16	1.33	-1.25, -1.07	
≥ 4.0kg	3.4	46	-0.82	1.10	-1.17, -0.46	
Missing/not weighed	7.2	100	-1.63	1.38	-1.91, -1.36	
Maternal Height						
Normal stature (≥145cm)	94.8	1308	-1.16	1.34	-1.25, -1.08	F(1, 1362)=20.26, *p*=<0.001
Short stature (<145cm)	5.2	71	-1.90	1.36	-2.21, -1.59	
Maternal BMI						
<18.5	15.6	215	-1.50	1.22	-1.68, -1.32	F(2, 1360)=5.72, *p*=0.003
18.5-24.99	69.2	952	-1.17	1.39	-1.27, -1.06	
≥ 25.0	15.2	209	-1.09	1.24	-1.31, -0.86	
Proximate factors						
Morbidity						
No	56.5	781	-1.16	1.40	-1.27, -1.05	F(1, 1364)=1.45, *p*=0.229
Yes	43.5	601	-1.26	1.28	-1.39, -1.13	
Breastfeeding						
No	29.9	413	-1.15	1.39	-1.30, -0.99	F(1, 1364)=0.66, *p*=0.4178
Yes	70.1	968	-1.22	1.33	-1.32, -1.12	
Bottle feeding						
No	66.0	911	-1.27	1.33	-1.38, -1.16	F(1, 1363)=5.39, *p*=0.020
Yes	34.0	469	-1.07	1.37	-1.20, -0.93	

For peer review only

Indicator	%	N	Mean HAZ	Std. deviation	95% CI for mean	Test statistic
Dietary Diversity Tercile						
Low	43.2	596	-1.30	1.20	-1.42, -1.17	F(2, 1363)=7.80, *p*=<0.001
Middle	33.8	467	-1.29	1.46	-1.44, -1.13	
High	23.1	318	-0.90	1.41	-1.07, -0.73	
Minimum meal frequency						
No	27.4	378	-1.32	1.38	-1.48, -1.16	F(1, 1364)=2.93, *p*=0.087
Yes	72.6	1003	-1.16	1.33	-1.25, -1.06	
Minimum acceptable diet						
No	68.4	945	-1.23	1.32	-1.33, -1.13	F(1, 1364)=1.13, *p*=0.287
Yes	31.6	436	-1.13	1.41	-1.29, -0.98	
Distal (underlying) factors						
Highest education level						
No education	12.0	166	-1.26	1.45	-1.50, -1.02	F(1, 1364)=6.90, *p*=0.009
Primary	55.6	768	-1.28	1.27	-1.40, -1.16	
Secondary/Higher	32.4	447	-1.04	1.42	-1.18, -0.91	
Labour force participation						
Not working	30.5	421	-1.19	1.22	-1.34, -1.04	F(2, 1359)=8.34, *p*=<0.001
Low	37.5	517	-1.40	1.42	-1.54, -1.26	
High	32.0	441	-0.98	1.34	-1.12, -0.84	
Household Wealth Index						
Poorest	25.4	345	-1.48	1.49	-1.65, -1.32	F(4, 1347)=7.53, *p*=<0.001
Poorer	18.2	248	-1.32	1.41	-1.50, -1.14	
Middle	19.9	271	-1.23	1.18	-1.41, -1.05	
Richer	17.7	241	-1.00	1.21	-1.20, -0.80	
Richest	18.8	256	-0.86	1.17	-1.04, -0.67	
Urban/rural residence						
Rural	86.4	1193	-1.25	1.23	-1.34, -1.16	F(1,1364)=13.04, *p*=<0.001
Urban	13.6	188	-0.88	1.97	-1.06, -0.70	
Sex of household head						
Male	77.0	1064	-1.20	1.33	-1.29, -1.10	F(1, 1364)=0.01, *p*=0.920
Female	23.0	317	-1.21	1.39	-1.40, -1.02	

For peer review only

Supplementary Table S4 Unstandardized and standardized results of the full structural path model examining the association between selected socioeconomic factors, mediating feeding practices and child HAZ, Cambodia (2014)

Path	Unstandardized (β)	Standardized (β)	R^2
Maternal employment (high)			0.15
Maternal education	0.167***	0.234***	
Household wealth			0.24
Maternal employment (high)	0.993***	0.327***	
Maternal education	0.706***	0.326***	
Dietary diversity			0.11
Maternal employment (high)	0.088	0.053	
Household wealth	0.097***	0.176***	
Breastfeeding	-0.191***	-0.111***	
Maternal education	0.081*	0.068*	
Breastfeeding			0.24
Maternal employment (high)	-0.071**	-0.073**	
Household wealth	-0.027**	-0.086**	
Maternal education	0.004	0.005	
HAZ			0.12
Maternal employment (high)	0.168	0.054	
Household wealth	0.058*	0.057*	
Dietary diversity	0.113*	0.061*	
Breastfeeding	-0.413***	-0.129***	
Maternal education	-0.019	-0.008	
**Overall R^2**			0.44
**χ^2 (df)**	65.70 (25)		
**RMSEA**	0.035		
**CFI**	0.972		

*p-value<0.05, **p-value<0.01, ***p-value<0.001

For peer review only

Supplementary Table S5 Proportion of women who reported breastfeeding at time of survey, by level of maternal participation in employment and child age group, Cambodia (2014)

	Age in months (*n*=1,365)				
	**6-8**	**9-11**	**12-17**	**18-23**	**6-23**
Not working or maternal employment (low)	95.3	93.6	85.7	42.3	75.5
Maternal employment (high)	82.6	78.8	72.0	34.0	59.0
X^2 test	X^2=8.52(1), *p*=0.004	X^2=7.06(1), *p*=0.008	X^2=7.52(1), *p*=0.006	X^2=2.03(1), *p*=0.155	X^2=24.72(1), *p*<0.001

Supplementary Table S6 Results of non-linear combinations for testing specific standardized indirect effects

**Path**	**Indirect Effect**			
	**Coef.**	**z**	**P-value**	**95% CI**
Maternal education -> Dietary diversity -> HAZ	0.004	1.63	0.104	(-0.00, 0.01)
Maternal education -> Breastfeeding -> HAZ	-0.001	-0.20	0.842	(-0.01, 0.01)
Maternal employment (high) -> Dietary diversity -> HAZ	0.003	1.41	0.159	(-0.00, 0.01)
Maternal employment (high) -> Breastfeeding -> HAZ	0.009	2.30	0.022	(0.00, 0.02)
Household wealth -> Dietary diversity -> HAZ	0.011	2.13	0.033	(0.00, 0.02)
Household wealth -> Breastfeeding -> HAZ	0.011	2.58	0.01	(0.00, 0.02)

For peer review only

Supplementary Figure S1 Mean height-for-age Z-scores by child age group and urban/rural residence (upper SD represented by error bar)

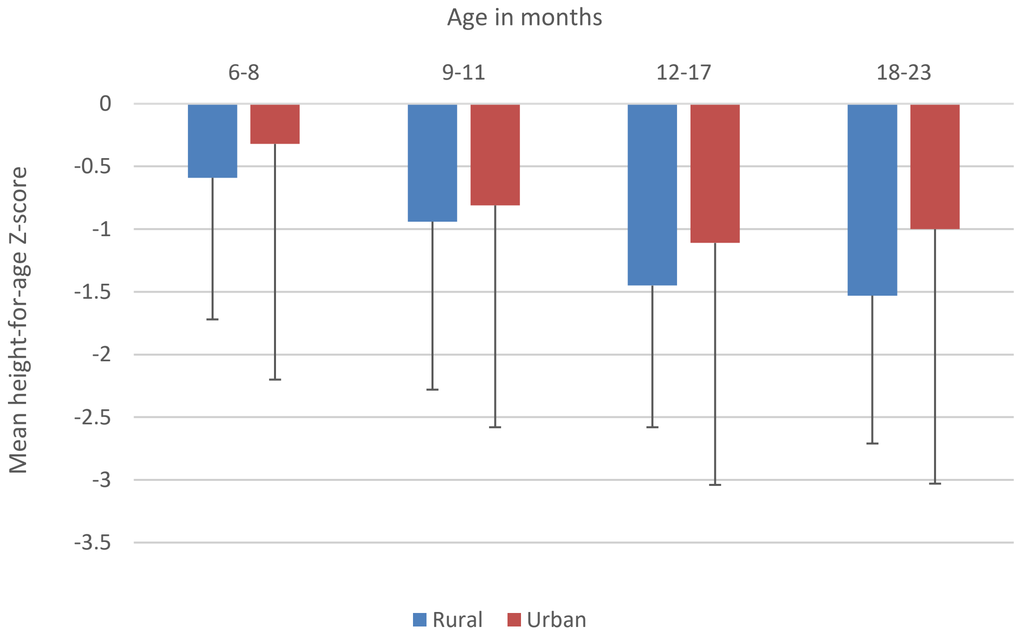

STROBE Statement—Checklist of items that should be included in reports of ***cross-sectional studies***

	Item No	Recommendation	Page No
**Title and abstract**	1	(*a*) Indicate the study's design with a commonly used term in the title or the abstract	1
		(*b*) Provide in the abstract an informative and balanced summary of what was done and what was found	2
**Introduction**			
Background/rationale	2	Explain the scientific background and rationale for the investigation being reported	4
Objectives	3	State specific objectives, including any prespecified hypotheses	5
**Methods**			
Study design	4	Present key elements of study design early in the paper	5
Setting	5	Describe the setting, locations, and relevant dates, including periods of recruitment, exposure, follow-up, and data collection	6
Participants	6	(*a*) Give the eligibility criteria, and the sources and methods of selection of participants	5
Variables	7	Clearly define all outcomes, exposures, predictors, potential confounders, and effect modifiers. Give diagnostic criteria, if applicable	6/7
Data sources/ measurement	8*	For each variable of interest, give sources of data and details of methods of assessment (measurement). Describe comparability of assessment methods if there is more than one group	*6/7*
Bias	9	Describe any efforts to address potential sources of bias	7-8
Study size	10	Explain how the study size was arrived at	
Quantitative variables	11	Explain how quantitative variables were handled in the analyses. If applicable, describe which groupings were chosen and why	5-6
Statistical methods	12	(*a*) Describe all statistical methods, including those used to control for confounding	7-9
		(*b*) Describe any methods used to examine subgroups and interactions	9
		(*c*) Explain how missing data were addressed	6
		(*d*) If applicable, describe analytical methods taking account of sampling strategy	6
		(*e*) Describe any sensitivity analyses	8/12-13
**Results**			
Participants	13*	(a) Report numbers of individuals at each stage of study—eg numbers potentially eligible, examined for eligibility, confirmed eligible, included in the study, completing follow-up, and analysed	5-6
		(b) Give reasons for non-participation at each stage	5-6
		(c) Consider use of a flow diagram	Supplementary S1
Descriptive data	14*	(a) Give characteristics of study participants (eg demographic, clinical, social) and information on exposures and potential confounders	10

For peer review only

		(b) Indicate number of participants with missing data for each variable of interest	10-12
Outcome data	15*	Report numbers of outcome events or summary measures	6
Main results	16	(*a*) Give unadjusted estimates and, if applicable, confounder-adjusted estimates and their precision (eg, 95% confidence interval). Make clear which confounders were adjusted for and why they were included	13/16
		(*b*) Report category boundaries when continuous variables were categorized	10
		(*c*) If relevant, consider translating estimates of relative risk into absolute risk for a meaningful time period	
Other analyses	17	Report other analyses done—eg analyses of subgroups and interactions, and sensitivity analyses	15-16
**Discussion**			
Key results	18	Summarise key results with reference to study objectives	17
Limitations	19	Discuss limitations of the study, taking into account sources of potential bias or imprecision. Discuss both direction and magnitude of any potential bias	19-20
Interpretation	20	Give a cautious overall interpretation of results considering objectives, limitations, multiplicity of analyses, results from similar studies, and other relevant evidence	17-20
Generalisability	21	Discuss the generalisability (external validity) of the study results	17-20
**Other information**			
Funding	22	Give the source of funding and the role of the funders for the present study and, if applicable, for the original study on which the present article is based	20

*Give information separately for exposed and unexposed groups.

Note: An Explanation and Elaboration article discusses each checklist item and gives methodological background and published examples of transparent reporting. The STROBE checklist is best used in conjunction with this article (freely available on the Web sites of PLoS Medicine at http://www.plosmedicine.org/, Annals of Internal Medicine at http://www.annals.org/, and Epidemiology at http://www.epidem.com/). Information on the STROBE Initiative is available at www.strobe-statement.org.

For peer review only

BMJ Open

Maternal socioeconomic status and infant feeding practices underlying pathways to child stunting in Cambodia: structural path analysis using cross-sectional population data

Journal:	*BMJ Open*
Manuscript ID	bmjopen-2021-055853.R2
Article Type:	Original research
Date Submitted by the Author:	01-Sep-2022
Complete List of Authors:	Harvey, Chloe; Independent Consultant<br>Newell, Marie-Louise; University of Southampton, Global Health Research Institute, School of Human Development and Health, Faculty of Medicine; University of the Witwatersrand, School of Public Health, Faculty of Health Science<br>Padmadas, sabu; University of Southampton, Social Statistics and Demography
**Primary Subject Heading**:	Nutrition and metabolism
Secondary Subject Heading:	Global health
Keywords:	Nutrition < TROPICAL MEDICINE, Community child health < PAEDIATRICS, PUBLIC HEALTH

SCHOLARONE™
Manuscripts

I, the Submitting Author has the right to grant and does grant on behalf of all authors of the Work (as defined in the below author licence), an exclusive licence and/or a non-exclusive licence for contributions from authors who are: i) UK Crown employees; ii) where BMJ has agreed a CC-BY licence shall apply, and/or iii) in accordance with the terms applicable for US Federal Government officers or employees acting as part of their official duties; on a worldwide, perpetual, irrevocable, royalty-free basis to BMJ Publishing Group Ltd ("BMJ") its licensees and where the relevant Journal is co-owned by BMJ to the co-owners of the Journal, to publish the Work in this journal and any other BMJ products and to exploit all rights, as set out in our licence.

The Submitting Author accepts and understands that any supply made under these terms is made by BMJ to the Submitting Author unless you are acting as an employee on behalf of your employer or a postgraduate student of an affiliated institution which is paying any applicable article publishing charge ("APC") for Open Access articles. Where the Submitting Author wishes to make the Work available on an Open Access basis (and intends to pay the relevant APC), the terms of reuse of such Open Access shall be governed by a Creative Commons licence – details of these licences and which Creative Commons licence will apply to this Work are set out in our licence referred to above.

Other than as permitted in any relevant BMJ Author's Self Archiving Policies, I confirm this Work has not been accepted for publication elsewhere, is not being considered for publication elsewhere and does not duplicate material already published. I confirm all authors consent to publication of this Work and authorise the granting of this licence.

For peer review only

Maternal socioeconomic status and infant feeding practices underlying pathways to child stunting in Cambodia: structural path analysis using cross-sectional population data

Chloe M. Harvey[1], Marie-Louise Newell[2,3] and Sabu S Padmadas[4]

[1]Independent Consultant, Bangkok, Thailand

[2]Global Health Research Institute, School of Human Development and Health, Faculty of Medicine, University of Southampton, Southampton, UK

[3]School of Public Health, Faculty of Health Science, University of the Witwatersrand, Johannesburg, South Africa

[4]Department of Social Statistics and Demography and Global Health Research Institute, University of Southampton, Southampton, UK

Corresponding author: Chloe M. Harvey, chloemercedes1991@gmail.com

Word count: 4563

For peer review only - http://bmjopen.bmj.com/site/about/guidelines.xhtml

ABSTRACT

Objectives: To identify and investigate complex pathways to stunting among children aged 6-24 months to determine the mediating effects of dietary diversity and continued breastfeeding on the association between socioeconomic factors and child stunting.

Design, setting and participants: We analysed the most recent cross-sectional Demographic and Health Survey data from Cambodia (2014). We applied structural path analysis on a sample of 1,365 children, to model the complex and inter-related pathways of factors determining children's height-for-age. Explanatory variables included a composite indicator of maternal employment, household wealth, maternal education, current breastfeeding status and dietary diversity score. Results are presented both in terms of non-standardized and standardized coefficients.

Outcome measure: The primary outcome measure was height-for-age Z-scores (HAZ) as a continuous measure.

Results: Findings suggest that children's dietary diversity and continued breastfeeding mediate the association between socioeconomic status and children's height. Whilst there was no significant direct effect of maternal education on children's height, results suggested significant indirect pathways through which maternal education effects children's height; operating through household wealth, maternal employment, dietary diversity and continued breastfeeding status *(p<0.001)*. Most notably, 41% of the effect of maternal employment on children's height was mediated by either dietary diversity or continued breastfeeding.

Conclusion: We provide evidence to support targeted nutrition interventions which account for the different ways in which underlying socioeconomic factors influence infant and young child feeding practices, and the potential impact on child nutritional status.

For peer review only - http://bmjopen.bmj.com/site/about/guidelines.xhtml

Article Summary

Strengths and limitations of this study

- Application of a causal hypothetical model to explain possible pathways to children's linear growth.
- Hypothetical models that can be applied to similar geographical contexts.
- The cross-sectional nature of DHS data, requires that results be interpreted with some caution.
- The theoretical model was built iteratively based on prior theory and thus, is not an exhaustive representation of the pathways to children's height.

For peer review only

For peer review only - http://bmjopen.bmj.com/site/about/guidelines.xhtml

INTRODUCTION

Across Southeast Asia, stunting in children under five years of age continues to pose a critical public health concern, with most countries in the region experiencing prevalence levels above 30%.[[dataset] [1]] In Cambodia, more than a third of children under the age of five years are stunted;[[dataset] [1]] stunting has been shown to be associated with distinct socioeconomic inequality, with sub-optimum feeding practices especially after 6 months of age, micronutrient deficiencies and recurrent infections.[[1]-[3]]

Impaired linear growth has adverse long-term health consequences for cognitive and immune system development, and is associated with increased risk of overweight and co-morbidities in later life.[[1], [4], [5]] Evidence shows that childhood stunting also has intergenerational implications, as stunting in pregnant women may lead to intrauterine growth restriction in her offspring,[[6]] thus continuing the vicious cycle of undernutrition. Interventions to tackle undernutrition in Southeast Asia have not been a top priority for governments and other stakeholders,[[7]] suggesting the need for strengthening further scientific evidence to understand the causal pathways of undernutrition, to identify appropriate strategies for policy and programme interventions.

Bio-behavioural and socioeconomic factors associated with children's height-for-age operate at varying levels of causation.[[1]] Limited dietary diversity has previously been reported to be significantly associated with impaired linear growth,[[8]] whereas the role of continued breastfeeding beyond six months remains inconclusive, with some studies even suggesting a negative association.[[9], [10]] However, it is not clear whether early feeding practices are directly and independently associated with height-for-age or whether they mediate the association between distal (underlying) factors and stunting.

Studies in Cambodia and other low- and middle-income countries have consistently shown maternal education, household wealth and maternal employment to be associated with inequalities in stunting prevalence.[[1], [11]-[15]] These three socioeconomic factors have also been associated with stark differentials in breastfeeding practices and dietary diversity within the Southeast Asian context.[[16]] However, associations underlying improved maternal socioeconomic status, continued breastfeeding and dietary diversity are paradoxical, as improvements in socioeconomic status (such as income, employment and education) are congruent with declines in breastfeeding, whereas improved socioeconomic status with increasing household income is positively associated with improved dietary diversity. Previous research has also indicated significant differentials in child nutritional status according to urban/rural residence,[[1], [15]] however, the intra-urban and intra-

For peer review only - http://bmjopen.bmj.com/site/about/guidelines.xhtml

rural differentials are rarely considered, despite research suggesting starker socioeconomic inequalities within urban areas in low- and middle-income country settings.[[17]]

Most cross-sectional studies to date focusing on factors associated with linear growth have examined children's height-for-age as a dichotomous outcome, expressed as stunting. Evidence on the complex and inter-related nature of factors associated with a continuous measure of children's height-for-age is limited, including modelling techniques that account for dynamic interactions between possible explanatory variables, to disentangle direct effects from indirect effects with mediating variables.[[18]-[20]] To date, no such study has focussed on the Southeast Asian region, including Cambodia, where evidence-based interventions are critical for tackling the population burden of child undernutrition.

Using cross-sectional data from the most recent National Demographic and Health Survey (CDHS) in Cambodia, we identify and investigate theoretical pathways to stunting among children aged 6-24 months, when breastfeeding and dietary practices are most important for growth. We use structural path analysis to examine two hypotheses:(1) socioeconomic factors are directly associated with dietary diversity and continued breastfeeding;(2) dietary diversity and continued breastfeeding beyond six months of age mediate the association between socioeconomic factors and children's height-for-age, partially explaining the association between underlying socioeconomic factors and the outcome (children's height).

METHODS

Data

We used the most recent Demographic and Health Survey (DHS) data from Cambodia,[[dataset] [2]] which adopted a two-stage stratified sampling by separating rural and urban areas. The survey interviewed 17,578 women aged 15-49 years from 15,825 households, which included a sample of women from 5,667 urban and 11,911 rural areas. Anthropometric measurements, child health and health care data were collected for only those births that occurred five years preceding the survey. All eligible mothers were asked about the breastfeeding status of their children under 36 months at the time of survey visit; 24 hour-recall of additional liquids and solid foods was collected for the youngest child <36 months.

The sample of interest for this study was the youngest, living singleton child, between the ages of 6 and <24 months at the time of survey, living in the same household as their mother. As per CDHS

For peer review only - http://bmjopen.bmj.com/site/about/guidelines.xhtml

(2014) sampling design, anthropometric measurements were only taken in two-thirds of households, and after accounting for missing or implausible anthropometric information, a total of 1,365 Cambodian children between the ages of 6 and <24 months were selected (Supplementary Table S1). The children's file contains the records for every child born in the five years preceding the survey for women aged 15-49 years, who were selected randomly from the sampled households within each cluster.

The response rate for the women's survey from which information about the children is collected, was 98%. Additional information on the CHDS sampling design and survey methodology is reported elsewhere (CDHS, 2014). Item non-response for the selected explanatory variables was minimal, and the decision was made to exclude cases with missing data at random from the final analysis (<0.5% of total cases).

No additional data is available.

Dependent variable

Anthropometric data were collected by trained interviewers using internationally standard instruments. Height measurements were taken using a SECA measuring board.[[dataset] [2]] For children aged less than 24 months, recumbent length measurements were taken and for older children, standing height was measured.[[dataset] [2]]

Children's height-for-age Z-scores were calculated by the DHS using the WHO Child Growth Standards.[[21]] Stunting was defined as height-for-age Z-score below minus two standard deviations (-2 SD) from the mean of the reference population and severe stunting as below minus three standard deviations (-3 SD).[[21]] We used height-for-age Z-scores (HAZ) as a continuous measure, assuming that explanatory variables have varying effect on the respective Z-score distribution.

Explanatory variables

Selection of explanatory variables was based on previous research on the association between infant and young child feeding practices and child stunting, as well as two conceptual frameworks which depict the pathways through which different factors may affect child height/growth at different levels.[[23], [24]] Children's dietary diversity and breastfeeding at time of survey were selected as the two main proximate factors in the hypothetical structural path models. This was on the basis of evidence from the Cambodian DHS that suggests the main reasons for Cambodian children not meeting the minimum acceptable diet was due to not meeting the recommended minimum dietary

For peer review only

For peer review only - http://bmjopen.bmj.com/site/about/guidelines.xhtml

diversity (consumption of four or more food groups in the previous 24 hours) but not due to meal frequency.[[dataset] [2]] Inclusion of continued breastfeeding status separately is important as while it is not included as a food group in the original WHO definition of minimum dietary diversity (MDD-7), it is recommended as an additional source of nutrients during the complementary feeding period.[[25]]

Terciles of dietary diversity were computed by age in months, based on the number of food groups consumed by children in the 24 hours preceding the survey,[[26]] to account for the gradual introduction of complementary foods.[[27]] Thus, children were regarded as having a low, middle or high diversity, relative to children of the same age. The definition of the terciles vary by age; Supplementary table S2 presents the distributions of the dietary diversity terciles by age.

Breastfeeding was categorised as a dichotomous variable as whether the child was being currently breastfed or not at child's age or time of survey.

The distal (underlying) factors included maternal education, household wealth and maternal employment. Maternal education reflected the highest level of completed education and was categorised into no education, primary, secondary and higher. Quintiles of household wealth were computed separately for urban and rural areas by DHS using principal component analysis, to ensure the representation of the poorest of the poor households, especially in rural areas.[[28]]

As children's nutritional status and feeding practices may be determined by a combination of different aspects and types of maternal employment,[[29], [30]] we created a composite indicator measuring maternal participation in the labour force based on an indicator computer by Sebayang, Efendi and Astutik.[[31]] Using principal component analysis, we incorporated five aspects of maternal employment: 1) working status over the past 12 months; 2) type of employment, whether self-employed or employed by family; 3) type of occupation; 4) type of earnings and 5) seasonality of work. Three distinctive categories of maternal employment were identified: not working, low level of participation, high level of participation, which was further dichotomised into a binary variable: (0) Not working/low participation; (1) High participation.

Statistical Analysis

Bivariate analyses included adjusted Wald tests for joint hypothesis testing to assess preliminary associations between explanatory variables and height-for-age Z-scores (Supplementary table S3). Statistical significance was considered at $p<0.05$ level.

For peer review only - http://bmjopen.bmj.com/site/about/guidelines.xhtml

205 Analysis was conducted using Stata/SE 15.1.[[32]] All descriptive and exploratory analyses accounted for the complex survey design of the CDHS using the appropriate sample weights.[[dataset] [2]]

208 Structural path analysis is the structural component of structural equation modelling and involves the modelling of observed variables only.[[33]] Path analysis facilitates the study of direct and indirect effects of multiple independent and dependent variables simultaneously, based on a generalized multi-equation framework for examining multiple hypotheses about interdependencies between these variables.[[34], [35]] The first step of analysis involved creating a hypothetical "causal" pathway diagram between the independent variables of interest and the outcome HAZ (Figure 1). The hypothesised model was built iteratively based on prior theory and research, and therefore not an exhaustive representation of the pathways to children's height-for-age. It was selected as the appropriate methodology for this analysis as it allows for the identification of indirect and direct mechanisms through which socioeconomic factors influence height-for-age, where dietary diversity and breastfeeding may act as mediators in these associations.

219 Structural equation modelling assumes linearity of observed variables, and the assumption of linear normality amongst pseudo-continuous variables was tested with likelihood ratio (LR) tests and BIC/AIC diagnostics.[[33]] Most independent variables showed evidence of linearity, justifying their inclusion in the model as continuous variables. However, this was not the case for maternal employment as a continuous independent variable, and we thus used the dummy variable to adjust for high level of participation in employment, as this was the category of interest for this study.

[Figure 1 Conceptual model of pathways between selected socioeconomic factors, dietary diversity, breastfeeding and height-for-age (HAZ) Z-scores (HAZ)]

Assessment of model fit

230 Structural path analysis was undertaken using the SEM program in Stata/SE 15.1. All models were estimated using maximum likelihood with missing values (MLMV). Goodness of fit of the data to the models were assessed using the root-mean-square error of approximation (RMSEA < 0.06) and the comparative fit index (CFI ≥ 0.95 for acceptance).[[36]] Both non-standardized and standardized coefficients are presented, but only standardized results are interpreted and presented on path diagrams (non-standardized coefficients use the raw/real life units of each variable and standardized coefficients convert the coefficients into comparable units). Standardized coefficients are

For peer review only - http://bmjopen.bmj.com/site/about/guidelines.xhtml

interpreted in terms of standard deviation differences.[[34]] To address the second hypothesis of this study that suggests dietary diversity and breastfeeding are mediators in the association between socioeconomic status and children's height-for-age Z-scores, standardized indirect effects were calculated.[[33]]

Patient and public involvement

Secondary data was used for this analysis. Therefore, no patient consent was needed.

RESULTS

Sample characteristics

Approximately one-third of children were aged between 6-12 months and two-thirds were aged 12-23 months (Table 1), with 52% boys and 48% girls; 6% of children were born with a low birth weight (< 2.5kg). Just over two-fifth (44%) of children were reported to have had morbidity, either a fever, respiratory condition or diarrhoea, in the two weeks prior to interview. Five percent of mothers were of short stature (< 145cm); 16% had low (<18.5) and 15% high (≥25.0) BMI. Most mothers had completed primary level education (56%), and only 12% reported no formal education. Approximately a third of women reported that they had not worked in the year prior to survey, a third of women had low level and the remainder high level of participation in employment. One in four of the children were from the poorest households and most households were located in rural areas (86%). About 70% of children aged 6-23 months were still being breastfed at the time of survey; 34% had been fed from a bottle. Approximately 43% of children had a low dietary diversity, but 73% of children consumed the recommended meal frequency.

For peer review only

For peer review only - http://bmjopen.bmj.com/site/about/guidelines.xhtml

Table 1 Summary characteristics of children 6-23 months from the Cambodian DHS (2014) (n=1,381*)

Indicator		%	N	Mean HAZ	Std. deviation	95% CI for mean	
						Lower bound	Upper bound
CHILD CHARACTERISTICS							
Age (months)	6-8	17.5	242	-0.55	1.25	-0.75	-0.35
	9-11	15.1	208	-0.92	1.48	-1.14	-0.70
	12-17	33.5	463	-1.41	1.23	-1.54	-1.28
	18-23	33.9	468	-1.46	1.32	-1.60	-1.31
Sex	Male	52.0	718	-1.28	1.34	-1.39	-1.16
	Female	48.0	663	-1.12	1.35	-1.24	-1.0
Birth weight	< 2.5kg	6.0	83	-1.48	1.50	-1.85	-1.11
	2.5 < 4.0kg	83.4	1152	-1.16	1.33	-1.25	-1.07
	≥ 4.0kg	3.4	46	-0.82	1.10	-1.17	-0.46
	Missing/not weighed	7.2	100	-1.63	1.38	-1.91	-1.36
Morbidity	No	56.5	781	-1.16	1.40	-1.27	-1.05
	Yes	43.5	601	-1.26	1.28	-1.39	-1.13
MATERNAL CHARACTERISTICS							
Height	Normal stature (≥145cm)	94.8	1308	-1.16	1.34	-1.25	-1.08
	Short stature (<145cm)	5.2	71	-1.90	1.36	-2.21	-1.59
BMI	<18.5	15.6	215	-1.50	1.22	-1.68	-1.32
	18.5-24.99	69.2	952	-1.17	1.39	-1.27	-1.06
	≥ 25.0	15.2	209	-1.09	1.24	-1.31	-0.86
Highest education level	No education	12.0	166	-1.26	1.45	-1.50	-1.02
	Primary	55.6	768	-1.28	1.27	-1.40	-1.16
	Secondary/Higher	32.4	447	-1.04	1.42	-1.18	-0.91
Maternal employment	Not working	30.5	421	-1.19	1.22	-1.34	-1.04
	Low	37.5	517	-1.40	1.42	-1.54	-1.26
	High	32.0	441	-0.98	1.34	-1.12	-0.84
HOUSEHOLD CHARACTERISTICS							
Household Wealth Index	Poorest	25.4	345	-1.48	1.49	-1.65	-1.32
	Poorer	18.2	248	-1.32	1.41	-1.50	-1.14
	Middle	19.9	271	-1.23	1.18	-1.41	-1.05
	Richer	17.7	241	-1.0	1.21	-1.20	-0.80
	Richest	18.8	256	-0.86	1.17	-1.04	-0.67
Urban/rural residence	Rural	86.4	1193	-1.25	1.23	-1.34	-1.16
	Urban	13.6	188	-0.88	1.97	-1.06	-0.70

For peer review only - http://bmjopen.bmj.com/site/about/guidelines.xhtml

Table 1 contd. Summary characteristics of children 6-23 months from the Cambodian DHS (2014) (n=1,381*)

Indicator		%	N	Mean HAZ	Std. deviation	95% CI for mean	
						Lower bound	Upper bound
Sex of household head	Male	77.0	1064	-1.20	1.33	-1.29	-1.10
	Female	23.0	317	-1.21	1.39	-1.40	-1.02
IYCF CHARACTERISTICS							
Breastfeeding	No	29.9	413	-1.15	1.39	-1.30	-0.99
	Yes	70.1	968	-1.22	1.33	-1.32	-1.12
Bottle feeding	No	66.0	911	-1.27	1.33	-1.38	-1.16
	Yes	34.0	469	-1.07	1.37	-1.20	-0.93
Dietary Diversity Tercile	Low	43.2	596	-1.30	1.20	-1.42	-1.17
	Middle	33.8	467	-1.29	1.46	-1.44	-1.13
	High	23.1	318	-0.90	1.41	-1.07	-0.73
Minimum meal frequency	No	27.4	378	-1.32	1.38	-1.48	-1.16
	Yes	72.6	1003	-1.16	1.33	-1.25	-1.06
Minimum acceptable diet	No	68.4	945	-1.23	1.32	-1.33	-1.13
	Yes	31.6	436	-1.13	1.41	-1.29	-0.98

* Sample size differs slightly from the sample used in the final models (*n*=1,365) due to missing data for some children on explanatory variables.

Results of structural path analysis

Underlying socioeconomic factors

Figure 2 represents the full structural path model; the standardized and unstandardized coefficients are presented in Supplementary Table S4. Overall, the fit of the model according to RMSEA (< 0.05) and CFI (>0.950) was good, and the model explained approximately 44% of the variance in HAZ scores (R^2=0.44).

Maternal education was significantly and positively associated with dietary diversity. One SD increase in the level of maternal education was associated with a 0.07 SD increase in dietary diversity (β = 0.07; *p*=0.023), but with no significant effect on breastfeeding status. Similarly, a one SD increase in household wealth was associated with a 0.18 SD increase in dietary diversity tercile (β = 0.18; *p*<0.001), but a one SD increase in household wealth was associated with -0.09 SD decrease in breastfeeding. A high level of maternal employment was associated with a positive, but

For peer review only

insignificant effect on dietary diversity tercile, but statistically significantly negatively associated with breastfeeding status (β=-0.07; *p*=0.008). Further analysis revealed that this effect on breastfeeding status was confirmed amongst younger age groups, but not in the 18-23 month age group (Supplementary table S5).

[Figure 2 Full structural path model, measuring how socioeconomic factors are associated with children's HAZ Z-scores directly and through mediators of dietary diversity and continued breastfeeding. Standardized coefficients. Cambodia (2014)]

Dietary diversity and continued breastfeeding beyond 6 months of age as mediating factors

Both hypothesised mediating variables – dietary diversity tercile and breastfeeding status – were independently and significantly associated with child HAZ score, although associations were in the opposite direction. One SD increase in dietary diversity was associated with a 0.06 SD increase in HAZ (*p*=0.017), whereas continued breastfeeding was associated with a -0.13 decrease in HAZ (*p*<0.001) (Figure 2 and Supplementary table S4). The only socioeconomic factor with a significant independent association with HAZ was household wealth, where a 1 SD increase in household wealth was associated with a 0.06 SD increase in HAZ (*p*=0.045).

To assess the role of dietary diversity and breastfeeding as mediators in the association between socioeconomic factors (maternal education, household wealth, maternal employment (high)) and HAZ, indirect pathways were examined (Table 2). In terms of maternal education, the standardized effects presented a case of 'inconsistent mediation',[[37]] where the direct and indirect effects do not have the same consistent sign. Furthermore, whilst the indirect effect is significant (*p*<0.001), the direct effect is not and hence, the indirect effect is larger than the total effect meaning that the effect of maternal education on HAZ is fully attributed to the indirect path which operates through household wealth, maternal employment, dietary diversity and breastfeeding status.

For peer review only - http://bmjopen.bmj.com/site/about/guidelines.xhtml

Table 2 Standardized coefficients of maternal education, household wealth, maternal employment and child height-for-age Z-score, with correlated residuals for dietary diversity and breastfeeding status

**Outcome**	**Direct effect (β)**	**Indirect effect (β)**	**Total effect**
HAZ			
Dietary diversity -> HAZ	0.061*	na	0.061*
Breastfeeding -> HAZ	-0.129***	-0.007	-0.136***
Maternal Education -> HAZ	-0.008	0.051***	0.043
Maternal employment (high) -> HAZ	0.054	0.039***	0.094**
Household wealth -> HAZ	0.057*	0.022**	0.080**

The significance levels are for the unstandardized solution.

*p-value<0.05, **p-value<0.01, ***p-value<0.001, na: not applicable

Although there was no direct effect of maternal employment on HAZ, statistically significant indirect effects suggested that 41% (0.039/0.094=0.41) of the effect of maternal employment on HAZ was mediated by either dietary diversity or breastfeeding status.

Household wealth was the only underlying (socioeconomic) factor with a statistically significant direct effect on HAZ, with 71% of the total effect on HAZ estimated to be direct (0.057/0.080=0.71), after controlling for dietary diversity and breastfeeding status, with the indirect effect of household wealth on HAZ an estimated 28% (0.022/0.080=0.28).

Further exploration of the specific indirect pathways between underlying (socioeconomic) factors and HAZ, through mediating variables of dietary diversity and breastfeeding, showed evidence of three indirect pathways that were statistically significant (Supplementary Table S6). High level of maternal employment had a small but significant standardized effect on HAZ, mediated by breastfeeding status of 0.01, z=2.30, p=0.022. Household wealth also had a small but statistically significant standardized effect on HAZ, mediated by both dietary diversity and breastfeeding status (0.01, z=2.13, p=0.033; 0.01, z=2.58, p=0.012 respectively).

For peer review only - http://bmjopen.bmj.com/site/about/guidelines.xhtml

DISCUSSION

The current study extends previous research examining factors associated with child stunting, to present a robust statistical model, which reflects the complex interactions between variables associated with children's height-for-age. Structural path analysis allows for the replication of a theoretical framework with underlying and proximate factors, to examine both the indirect and direct effect of factors known to be associated with child stunting. The structural path model presented could be applicable to other low- and middle-income country contexts and could examine whether our proposed pathways to stunting are consistent across geographical contexts.

Overall, the results of this study confirmed that improvements in socioeconomic status such as maternal education, household wealth and participation in employment, were associated with an increase in children's dietary diversity, but a decrease in continued breastfeeding beyond 6 months of age. The findings also suggested that dietary diversity and continued breastfeeding mediate the associations between socioeconomic status and height-for-age Z-scores. High level of maternal employment had a small but significant association with HAZ that was mediated by breastfeeding status, while the significant association between household wealth and HAZ was mediated by both dietary diversity and breastfeeding status. This suggests that interventions to improve child nutritional status should make due consideration for the underlying socioeconomic drivers which may influence infant and young child feeding practices. Women's employment conditions is one such factor that has been found to influence infant feeding practices globally, especially early weaning among mothers returning to work. [[38]] Although the female labour force participation rate is relatively high in Cambodia, with 74% of women participating in the labour force, [[39]] Cambodia has one of the highest proportions of informally employed (93.1 % among men and women), [[40]] which is often associated with a lack of access to maternity protection. [[41]]

The independent effect of maternal education on dietary diversity was small but significant, consistent with the findings of other studies conducted in low- and middle-income countries,[[42]-[45]] in addition to studies which confirmed a strong association between increasing household wealth and improvements in dietary diversity.[[15], [46]] The significant pathway between breastfeeding and dietary diversity demonstrated an additional, interesting observation, that continued breastfeeding beyond 6 months of age, was associated with a less diverse diet, even after adjusting for age. This could be indicative of the situation in poorer households where prolonged breastfeeding may be practiced to make up for the lack of complementary foods, as suggested from a study of feeding practices in Thailand.[[9]]

For peer review only - http://bmjopen.bmj.com/site/about/guidelines.xhtml

In light of the nutrition transition, which has been occurring in many low- and middle-income countries, Popkin and Bisgrove [[47]] reported that duration of breastfeeding was declining in urban areas as a result of the increased maternal education and employment opportunities that arise from urbanisation. Furthermore, the negative association between increasing household wealth and continued breastfeeding is perhaps underscored by the increasing trend in use of breast milk substitutes among children aged over six months of age in Cambodia.[[48]] Although the Cambodian government adopted many aspects of the *International Code of Marketing of Breast-milk Substitutes* [[49]] in the Cambodian *Sub-Decree on Marketing of Products for Infant and Young Child Feeding (Sub-Decree 133),* [[50]] a recent pilot implementation of a monitoring and enforcement system found significant non-compliance with this Sub-Decree at point-of-sales and in health facilities. [[51]]

Our study confirmed that dietary diversity and continued breastfeeding mediate associations between socioeconomic status and child stunting, however these findings were only significant for pathways originating from maternal employment and household wealth. The results of this study suggested a positive and direct association between Cambodian children's dietary diversity and height-for-age z-scores, which has also been supported by other studies conducted in Cambodia.[[52], [53]] Moreover, dietary diversity was a significant mediator of the association between household wealth and HAZ suggesting that the positive effect of increasing household wealth can partially be explained by improved dietary diversity. This highlights the need to prioritise targeted interventions within the poorest households to improve dietary diversity to prevent child stunting. A further 71% of the total effect of household wealth on HAZ was found to be direct, suggesting the importance of other mediating factors not included in this model but known to be associated with child stunting, such as access to health care, WASH facilities and immunization status.

On the other hand, breastfeeding after age 6 months was associated with a negative, direct effect on children's HAZ in Cambodia in the path model that adjusted for age of the child. This was also noted by Arimond and Ruel,[[27]] in a study using Cambodian DHS data from 2000, and in a review of 13 studies by Grummer-Strawn.[[54]] A negative effect of breastfeeding beyond age six months on children's height-for-age status has been discussed by many researchers. One proposed explanation is reverse causality, whereby the decision to wean is related to mother's perceptions of size and stature of the child, hence malnourished children may be more likely to be breastfed for longer durations.[[55]] However, as Martin[[56]] points out, due to the cross-sectional nature of structural path analysis, it is not possible to account for the direction in the relationship between prolonged

For peer review only - http://bmjopen.bmj.com/site/about/guidelines.xhtml

breastfeeding and HAZ. In terms of the indirect effects, structural path analysis revealed that breastfeeding was a small but significant mediator in the association between maternal employment and HAZ, as well as household wealth and HAZ.

STRENGTHS AND LIMITATIONS

This study used population-based data from the most recent nationally representative household survey from Cambodia, and our findings are generalizable to Cambodia. DHS data uses standard instruments to measure height by professionally trained investigators, which ensures accuracy of anthropometric measurements, however, the way in which continued breastfeeding and dietary diversity are reported using current-status data (from a 24 hour recall questionnaire) may introduce measurement bias, although this would apply equally to stunted and non-stunted children. Although the 2014 Cambodian DHS is the latest available household survey data for Cambodia, findings should be interpreted with caution when referring to the current context as the data is eight years old. Application of a causal hypothetical model to explain possible pathways to children's linear growth, presents the first study of its kind using Cambodian DHS data, and lays the foundation for similar studies in other geographical contexts. However, the cross-sectional nature of DHS data and the use of structural path analysis, typically a form of causal modelling, requires that the results be interpreted with some caution. Although structural path models may offer benefits over multivariable fixed effects models in allowing for complex interactions between variables, the theoretical model was built iteratively based on prior theory and thus, is by no means an exhaustive representation of the pathways to children's HAZ scores.

CONCLUSIONS

The theoretical structural path model presented in this study proposes a plausible illustration of the pathways associated with children's height-for-age in the Cambodian context. Overall, results confirmed that children's dietary diversity and continued breastfeeding status mediate the associations between socioeconomic status and linear growth, at least for pathways from maternal employment and household wealth. Analysis suggests that participation in maternal employment presents a significant barrier to continued breastfeeding beyond age six months of age, emphasising a need for maternity legislation that supports optimal breastfeeding practices, in line with WHO recommendations that infants be exclusively breastfed for six months, followed by continued breastfeeding in addition to complementary feeding up to two years of age.[[25]] Improvements to maternity legislation should also be inclusive of women working in the informal sector (of which 96%

For peer review only - http://bmjopen.bmj.com/site/about/guidelines.xhtml

of employed women in Cambodia participate). In low- and middle-income contexts such as Cambodia, where there are distinct urban and rural differentials in the incidence of stunting and where rapid urbanization has caused a shift in the geographical patterning of stunting, may warrant further investigation. Thus, future research may extend this analysis to further consider the moderating effect of urban/rural residence on the proposed structural path model using simultaneous path analysis.

CONTRIBUTORS

CMH conceptualised the study, prepared the dataset for research and conducted statistical analysis under the supervision of MLN and SSP. MLN and SSP contributed to the interpretation and revised paper for intellectual content. CMH, MLN and SSP conducted the final review and revised the manuscript for submission.

COMPETING INTERESTS

None declared.

FUNDING

This research was carried out as a part of a doctoral research project, supported by the UK Economic and Social Research Council (grant number ES/J500161/1). The Economic and Social Research Council had no role in the design, analysis or writing of this article.

DATA SHARING

Data are available in a public, open access repository. Available: https://dhsprogram.com/data/

ETHICS STATEMENT

For peer review only - http://bmjopen.bmj.com/site/about/guidelines.xhtml

This study is based on publicly available DHS data. CMH was granted access to the data by the MEASURE DHS/ICF International, Rockville, Maryland, USA.

REFERENCES

[dataset] [1] United Nations Children's Fund (UNICEF). Data from: Stunting (Survey Estimates, National and Disaggregated). April 11, 2021. https://data.unicef.org/topic/nutrition/malnutrition/

[2] Black RE, Victora CG, Walker SP, et al. Maternal and child undernutrition and overweight in low-income and middle-income countries. *The Lancet* 2013;382:427–451.

[3] Dewey KG, Mayers DR. Early child growth: How do nutrition and infection interact? *Matern Child Nutr* 2011;*7*(Suppl 3):129–142.

[4] Black RE, Allen LH, Bhutta ZA, et al. Maternal and child undernutrition: global and regional exposures and health consequences. *The Lancet* 2008;371:243–260

[5] Victora CG, Adair L, Fall C, et al. Maternal and child undernutrition: consequences for adult health and human capital. *The Lancet* 2008;371:340–357.

[6] Ozaltin E, Hill K, Subramanian SV. Association of maternal stature with offspring mortality, underweight, and stunting in low- to middle-income countries. *JAMA*, 2010;303:1507-1516.

[7] Mutunga M, Frison S, Rava M, et al. The Forgotten Agenda of Wasting in Southeast Asia: Burden, Determinants and Overlap with Stunting: A Review of Nationally Representative Cross-Sectional Demographic and Health Surveys in Six Countries. *Nutrients* 2020;12:559.

[8] Krasevec J, An X, Kumapley, R, et al. Diet quality and risk of stunting among infants and young children in low- and middle-income countries. *Matern Child Nutr* 2017;13(Suppl 2):1–11.

[9] Cetthakrikul N, Topothai C, Suphanchaimat R, et al. Childhood stunting in Thailand: When prolonged breastfeeding interacts with household poverty. *BMC Pediatr* 2018;18: 1–9

[10]Marquis GS, Habicht JP, Lanata CF, et al. Association of breastfeeding and stunting in Peruvian toddlers: An example of reverse causality. *Int J Epidemiol* 1997;26:349–356.

[11]Boyle M, Georgiades K, Racine Y, et al. The influence of economic development level, household wealth and maternal education on child health in the developing world. *Soc Sci Med* 2006;63:2242–2254.

For peer review only - http://bmjopen.bmj.com/site/about/guidelines.xhtml

[12]Caldwell JC. Education as a Factor in Mortality Decline An Examination of Nigerian Data. *Popul Stud (NY)* 1979;33:395–413.

[13]Caldwell JC. How is greater maternal education translated into lower child mortality? *Health Transit Rev* 1994;4:224–229.

[14]Popkin BM, Solon FS. Income, time, the working mother and child nutriture. *J Trop Pediatr* 1976;22:156–166.

[15]Smith LC, Ruel MT, Ndiaye A. Why is child malnutrition lower in urban than in rural areas? Evidence from 36 developing countries. *World Dev* 2005;33:1285–1305.

[16]Harvey CM, Newell ML, Padmadas SS. Socio-economic differentials in minimum dietary diversity among young children in minimum dietary diversity among young children in South-East Asia: evidence from Demographic and Health Surveys. *Public Health Nutr* 2018;21:3048-3057.

[17]Menon P, Ruel MT, Morris SS. Socio-economic differentials in child stunting are consistently larger in urban than rural areas. *Food Nutr Bull* 2000;21:282-289.

[18]Hanieh S, Ha TT, De Livera AM, et al. Antenatal and early infant predictors of postnatal growth in rural Vietnam: A prospective cohort study. *Arch Dis Child* 2015;100:165–173.

[19]Lander RL, Williams SM, Costa-Ribeiro H, et al. Understanding the complex determinants of height and adiposity in disadvantaged daycare preschoolers in Salvador, NE Brazil through structural equation modelling. *BMC Public Health* 2015;15:1–8.

[20]Cunningham K, Ferguson E, Ruel M, et al. Water, sanitation, and hygiene practices mediate the association between women's empowerment and child length-for-age z-scores in Nepal. *Matern Child Nutr* 2018;15:1–9.

[dataset] [21] National Institute of Statistics, Directorate General for Health, & ICF International. Data from: Cambodia Demographic and Health Survey 2014. The DHS Program, August 17, 2018. https://dhsprogram.com/methodology/survey/survey-display-464.cfm

[22]World Health Organization (WHO). *WHO Multicentre Growth Reference Study Group. WHO Child Growth Standards: Length/height-for-age, weight-for-age, weight-for-length, weight-for-height and body mass index-for-age: Methods and development*. Geneva: WHO, 2006. https://www.who.int/publications/i/item/924154693X

[23]Stewart CP, Iannotti L, Dewey KG, et al. Contextualising complementary feeding in a broader framework for stunting prevention. *Matern Child Nutr* 2013;9(Suppl 2): 27–45.

[24]Wamani H, Åstrøm AN, Peterson S, et al. Infant and young child feeding in Western Uganda: Knowledge, practices and socio-economic correlates. *J Trop Pediatr* 2005;51:356–361.

For peer review only

[25]World Health Organization (WHO) Continued breastfeeding for healthy growth and development of children [Internet]. WHO, 2017. Available: https://www.who.int/elena/titles/bbc/continued_breastfeeding/en/ [Accessed 02 Sept 2019].

[26]World Health Organization (WHO). *Indicators for assessing infant and young child feeding practices, Part 1: Definitions*. Geneva: WHO, 2008. https://apps.who.int/iris/bitstream/handle/10665/43895/9789241596664_eng.pdf;jsessionid=42E6F2DE11CA32FA51E4636A4301253A?sequence=1

[27]Arimond M, Ruel MT. Dietary diversity is associated with child nutritional status: evidence from 11 demographic and health surveys. *J Nutr* 2004;*134:*2579–2585.

[28]Rutstein SO. The DHS Wealth Index. DHS Working Paper. *USAID* 2008;60 [Working Paper] https://www.researchgate.net/publication/238706094_The_DHS_Wealth_Index_Approaches_for_Rural_and_Urban_Areas

[29]Nankinga O, Kwagala B, Walakira EJ. Maternal employment and child nutrition status in Uganda. *PloS One* 2019;14(e0226720). https://journals.plos.org/plosone/article?id=10.1371/journal.pone.0226720 (accessed 5 August 2020).

[30]Debela BL, Gehrke E, Qaim M. Links between Maternal Employment and Child Nutrition in Rural Tanzania. *Am J Agric Econ* 2020;103:812-830.

[31]Sebayang SK, Efendi F, Astutik E. Women's empowerment and the use of antenatal care services: analysis of demographic health surveys in five Southeast Asian countries. *Women Health* 2017;59:1-17.

[32][software] StataCorp. *Stata Statistical Software: Release 15*. StataCorp LLC 2017.

[33]Acock A. Discovering Structural Equation Modeling Using Stata. Texas, US: Stata Press 2013.

[34]Grace J, Bollen K. Interpreting the Results from Multiple Regression and Structural Equation Models. *Bulletin Ecological Society of America* 2005;86:283–295.

[35]Hox JJ, Bechger T. An Introduction to Structural Equation Modeling. *Family Science Review* 1999;11:354–373.

[36]Schreiber JB, Nora A, Stage FK. Reporting Structural Equation Modelling and Confirmatory Factor Analysis Results: A Review. *J Educ Res* 2006;99:323–338.

[37]Mackinnon DP, Fairchild AJ, Fritz MS. Mediation Analysis. *Annu Rev Psychol* 2007 ;58 :1–22.

[38]Rollins NC, Bhandari N, Hajeebhoy N, et al. Why invest, and what it will take to improve breastfeeding practices? *Lancet* 2016;387:491-504.

For peer review only

For peer review only - http://bmjopen.bmj.com/site/about/guidelines.xhtml

[39]ILOSTAT. Labour force participation rate, female – Cambodia. 2021. Available : https://data.worldbank.org/indicator/SL.TLF.CACT.FE.ZS?locations=KH (accessed 5 August 2020)

[40]International Labour Organization (ILO). *Women and men in the informal economy: a statistical picture (third edition).* Geneva: ILO, 2018. https://www.ilo.org/wcmsp5/groups/public/---dgreports/---dcomm/documents/publication/wcms_626831.pdf

[41]Addati L, Cassirer N, Gilchrist K. *Maternity and paternity at work: Law and practice across the world.* Geneva: ILO, 2014. https://www.ilo.org/wcmsp5/groups/public/---dgreports/---dcomm/---publ/documents/publication/wcms_242615.pdf

[42]Na M, Aguayo VM, Arimond M, et al. Trends and predictors of appropriate complementary feeding practices in Nepal: An analysis of national household survey data collected between 2001 and 2014. *Matern Child Nutr* 2018;14(Suppl 4):1–13.

[43]Patel A, Pusdekar Y, Badhoniya N, et al. Determinants of inappropriate complementary feeding practices in young children in India: secondary analysis of National Family Health Survey 2005 – 2006. *Matern Child Nutr* 2012;8(Suppl 1):28–44.

[44]Kabir I, Khanam M, Agho KE, et al. Determinants of inappropriate complementary feeding practices in infant and young children in Bangladesh: secondary data analysis of Demographic Health Survey 2007. *Matern Child Nutr* 2012;8(Suppl 1):11–27.

[45]Senarath U, Agho KE, Akram DES, et al. Comparisons of complementary feeding indicators and associated factors in children aged 6-23 months across five South Asian countries. *Matern Child Nutr* 2012;8(Suppl 1):89–106.

[46]Iannotti LL, Robles M, Pachón H, et al. Food Prices and Poverty Negatively Affect Micronutrient Intakes in Guatemala. *J Nutr* 2012;142:1568–1576.

[47]Popkin BM, Bisgrove EZ. Nutrition and Urbanization (Part 2): Urbanization and Nutrition in low-Income countries. *Food Nutr Bull* 1988;10:1–22.

[48]Prak S, Dahl MI, Oeurn S, et al. Breastfeeding Trends in Cambodia, and the Increased Use of Breast-Milk Substitute—Why Is It a Danger? *Nutrients* 2014;6:2920–2930.

[49]World Health Organization (WHO). *International Code of Marketing of Breastmilk Substitutes.* Geneva: WHO, 1981. https://apps.who.int/iris/handle/10665/40382

[50]Royal Government of Cambodia. *Sub-Decree on Marketing of Products for Infant and Young Child Feeding, No. 133, A Nor Kra Bor.* Phnom Penh: Royal Government of Cambodia, 2005.

For peer review only

[51]Hou L, Green M, Chum S, et al. Pilot implementation of monitoring and enforcement system for the International Code of Marketing of Breast-milk Substitutes in Cambodia. *Maternal and Child Nutrition* 2019; e12795.

[52]Greffeuille V, Sophonneary P, Laillou A, et al. Persistent inequalities in child undernutrition in Cambodia from 2000 until today. *Nutrients* 2016;8:1–17.

[53]Darapheak C, Takano T, Kizuki M, et al. Consumption of animal source foods and dietary diversity reduce stunting in children in Cambodia. *Int Arch Med* 2013;6:1–11.

[54]Grummer-Strawn L. Regression analysis of current-status data: an application to breast-feeding. *J Am Stat Assoc* 1993;88:758–765.

[55]Simondon K, Costes R, Delaunay V, et al. Children's height, health and appetite influence mothers' weaning decisions in rural Senegal. *Int J Epidemiol* 2001;30:476–481.

[56]Martin RM. Commentary: Does breastfeeding for longer cause children to be shorter? *Int J Epidemiol* 2001;30:481–484.

For peer review only

For peer review only - http://bmjopen.bmj.com/site/about/guidelines.xhtml

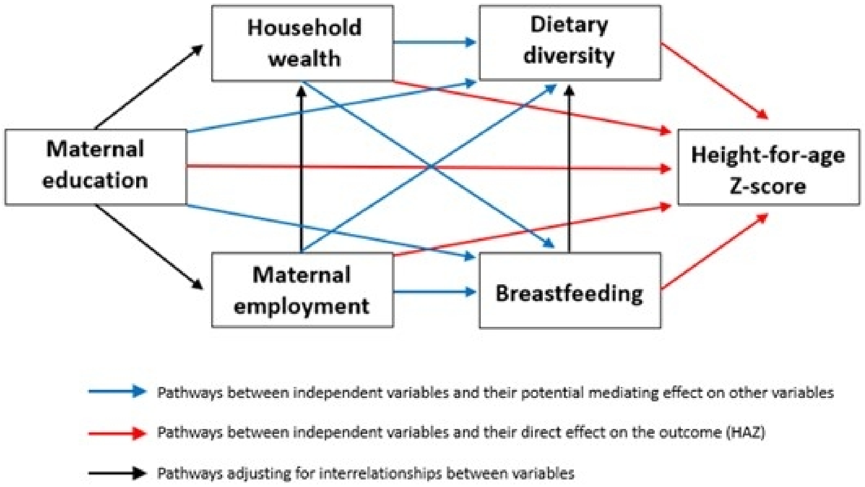

Figure 1 Conceptual model of pathways between selected socioeconomic factors, dietary diversity, breastfeeding and height-for-age (HAZ) Z-scores (HAZ)

162x107mm (96 x 96 DPI)

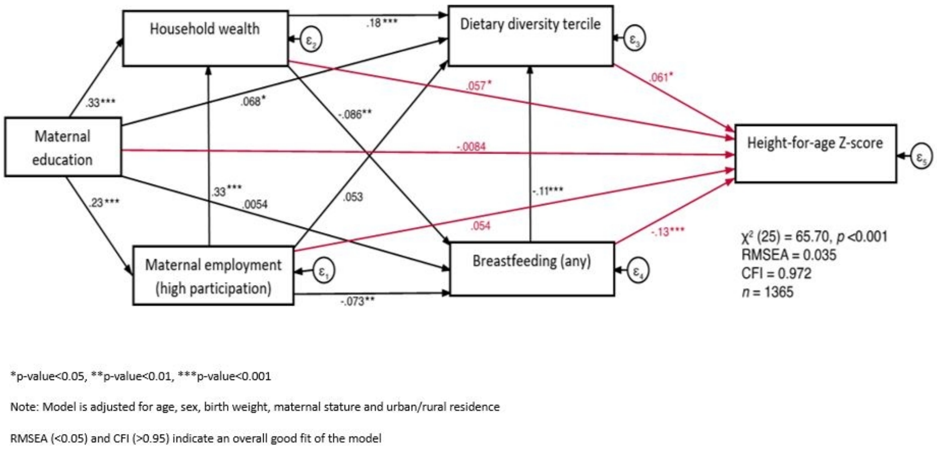

Figure 2 Full structural path model, measuring how socioeconomic factors are associated with children's HAZ Z-scores directly and through mediators of dietary diversity and continued breastfeeding. Standardized coefficients. Cambodia (2014)

259x137mm (96 x 96 DPI)

Supplementary Table S1. Sample size selection: children aged 6 to <24 months with plausible anthropometric measurements, Cambodia (2014)

	n
Children 6-23.9 months	2,127
Children 6-23.9 months with anthropometric measurements	1,397
**Children 6-23.9 months with plausible† anthropometric measurements**	**1,365**

† Plausible HAZ scores should fall between -6 and +6 standard deviations, according to the WHO SD flag limits (WHO, 2006)

Supplementary Table S2. Distribution of dietary diversity tercile by child's age, adjusted estimates, Cambodia (2014)

	**Age in months (*n* = 1,381)**								
	**6-8 (*n*=242)**		**9-11 (*n*=208)**		**12-17 (*n*=463)**		**18-23 (*n*=468)**		**6-23 (*n*=1,381)**
**Dietary Diversity Tercile**	%	**DDS range**	%	**DDS range**	%	**DDS range**	%	**DDS range**	%
Low	44.9	0-1 groups	41.0	0-2 groups	49.5	0-3 groups	37.0	0-3 groups	**43.2**
Middle	17.9	2 groups	43.0	3-4 groups	26.2	4 groups	45.4	4-5 groups	**33.8**
High	37.2	≥ 3 groups	16.0	≥ 5 groups	24.4	≥ 5 groups	17.6	≥ 6 groups	**23.1**

For peer review only

Supplementary Table S3. Association between IYCF indicators, socio-demographic factors and mean height-for-age Z-scores in children aged 6-23 months in Cambodia (2014), (estimates adjusted for survey design using appropriate sample weights)

Indicator	%	N	Mean HAZ	Std. deviation	95% CI for mean	Test statistic
Inherent (biological) factors						
Age (months)						
6-8	17.5	242	-0.55	1.25	-0.75, -0.35	F(3, 1362)=22.20, *p*=<0.001
9-11	15.1	208	-0.92	1.48	-1.14, -0.70	
12-17	33.5	463	-1.41	1.23	-1.54, -1.28	
18-23	33.9	468	-1.46	1.32	-1.60, -1.31	
Sex						
Male	52.0	718	-1.28	1.34	-1.39, -1.16	F(1, 1364)=3.33, *p*=0.068
Female	48.0	663	-1.12	1.35	-1.24, -1.00	
Birth weight						
< 2.5kg	6.0	83	-1.48	1.50	-1.85, -1.11	F(3, 1362)=5.65, *p*=<0.001
2.5 < 4.0kg	83.4	1152	-1.16	1.33	-1.25, -1.07	
≥ 4.0kg	3.4	46	-0.82	1.10	-1.17, -0.46	
Missing/not weighed	7.2	100	-1.63	1.38	-1.91, -1.36	
Maternal Height						
Normal stature (≥145cm)	94.8	1308	-1.16	1.34	-1.25, -1.08	F(1, 1362)=20.26, *p*=<0.001
Short stature (<145cm)	5.2	71	-1.90	1.36	-2.21, -1.59	
Maternal BMI						
<18.5	15.6	215	-1.50	1.22	-1.68, -1.32	F(2, 1360)=5.72, *p*=0.003
18.5-24.99	69.2	952	-1.17	1.39	-1.27, -1.06	
≥ 25.0	15.2	209	-1.09	1.24	-1.31, -0.86	
Proximate factors						
Morbidity						
No	56.5	781	-1.16	1.40	-1.27, -1.05	F(1, 1364)=1.45, *p*=0.229
Yes	43.5	601	-1.26	1.28	-1.39, -1.13	
Breastfeeding						
No	29.9	413	-1.15	1.39	-1.30, -0.99	F(1, 1364)=0.66, *p*=0.4178
Yes	70.1	968	-1.22	1.33	-1.32, -1.12	
Bottle feeding						
No	66.0	911	-1.27	1.33	-1.38, -1.16	F(1, 1363)=5.39, *p*=0.020
Yes	34.0	469	-1.07	1.37	-1.20, -0.93	

For peer review only

Indicator	%	N	Mean HAZ	Std. deviation	95% CI for mean	Test statistic
Dietary Diversity Tercile						
Low	43.2	596	-1.30	1.20	-1.42, -1.17	F(2, 1363)=7.80, *p*=<0.001
Middle	33.8	467	-1.29	1.46	-1.44, -1.13	
High	23.1	318	-0.90	1.41	-1.07, -0.73	
Minimum meal frequency						
No	27.4	378	-1.32	1.38	-1.48, -1.16	F(1, 1364)=2.93, *p*=0.087
Yes	72.6	1003	-1.16	1.33	-1.25, -1.06	
Minimum acceptable diet						
No	68.4	945	-1.23	1.32	-1.33, -1.13	F(1, 1364)=1.13, *p*=0.287
Yes	31.6	436	-1.13	1.41	-1.29, -0.98	
Distal (underlying) factors						
Highest education level						
No education	12.0	166	-1.26	1.45	-1.50, -1.02	F(1, 1364)=6.90, *p*=0.009
Primary	55.6	768	-1.28	1.27	-1.40, -1.16	
Secondary/Higher	32.4	447	-1.04	1.42	-1.18, -0.91	
Labour force participation						
Not working	30.5	421	-1.19	1.22	-1.34, -1.04	F(2, 1359)=8.34, *p*=<0.001
Low	37.5	517	-1.40	1.42	-1.54, -1.26	
High	32.0	441	-0.98	1.34	-1.12, -0.84	
Household Wealth Index						
Poorest	25.4	345	-1.48	1.49	-1.65, -1.32	F(4, 1347)=7.53, *p*=<0.001
Poorer	18.2	248	-1.32	1.41	-1.50, -1.14	
Middle	19.9	271	-1.23	1.18	-1.41, -1.05	
Richer	17.7	241	-1.00	1.21	-1.20, -0.80	
Richest	18.8	256	-0.86	1.17	-1.04, -0.67	
Urban/rural residence						
Rural	86.4	1193	-1.25	1.23	-1.34, -1.16	F(1,1364)=13.04, *p*=<0.001
Urban	13.6	188	-0.88	1.97	-1.06, -0.70	
Sex of household head						
Male	77.0	1064	-1.20	1.33	-1.29, -1.10	F(1, 1364)=0.01, *p*=0.920
Female	23.0	317	-1.21	1.39	-1.40, -1.02	

For peer review only

Supplementary Table S4 Unstandardized and standardized results of the full structural path model examining the association between selected socioeconomic factors, mediating feeding practices and child HAZ, Cambodia (2014)

Path	Unstandardized (β)	Standardized (β)	R^2
Maternal employment (high)			0.15
Maternal education	0.167***	0.234***	
Household wealth			0.24
Maternal employment (high)	0.993***	0.327***	
Maternal education	0.706***	0.326***	
Dietary diversity			0.11
Maternal employment (high)	0.088	0.053	
Household wealth	0.097***	0.176***	
Breastfeeding	-0.191***	-0.111***	
Maternal education	0.081*	0.068*	
Breastfeeding			0.24
Maternal employment (high)	-0.071**	-0.073**	
Household wealth	-0.027**	-0.086**	
Maternal education	0.004	0.005	
HAZ			0.12
Maternal employment (high)	0.168	0.054	
Household wealth	0.058*	0.057*	
Dietary diversity	0.113*	0.061*	
Breastfeeding	-0.413***	-0.129***	
Maternal education	-0.019	-0.008	
**Overall R^2**			0.44
**χ^2 (df)**	65.70 (25)		
**RMSEA**	0.035		
**CFI**	0.972		

*p-value<0.05, **p-value<0.01, ***p-value<0.001

For peer review only

Supplementary Table S5 Proportion of women who reported breastfeeding at time of survey, by level of maternal participation in employment and child age group, Cambodia (2014)

	Age in months (*n*=1,365)				
	**6-8**	**9-11**	**12-17**	**18-23**	**6-23**
Not working or maternal employment (low)	95.3	93.6	85.7	42.3	75.5
Maternal employment (high)	82.6	78.8	72.0	34.0	59.0
X^2 test	X^2=8.52(1), *p*=0.004	X^2=7.06(1), *p*=0.008	X^2=7.52(1), *p*=0.006	X^2=2.03(1), *p*=0.155	X^2=24.72(1), *p*<0.001

Supplementary Table S6 Results of non-linear combinations for testing specific standardized indirect effects

**Path**	**Indirect Effect**			
	**Coef.**	**z**	**P-value**	**95% CI**
Maternal education -> Dietary diversity -> HAZ	0.004	1.63	0.104	(-0.00, 0.01)
Maternal education -> Breastfeeding -> HAZ	-0.001	-0.20	0.842	(-0.01, 0.01)
Maternal employment (high) -> Dietary diversity -> HAZ	0.003	1.41	0.159	(-0.00, 0.01)
Maternal employment (high) -> Breastfeeding -> HAZ	0.009	2.30	0.022	(0.00, 0.02)
Household wealth -> Dietary diversity -> HAZ	0.011	2.13	0.033	(0.00, 0.02)
Household wealth -> Breastfeeding -> HAZ	0.011	2.58	0.012	(0.00, 0.02)

For peer review only

STROBE Statement—Checklist of items that should be included in reports of ***cross-sectional studies***

	Item No	Recommendation	Page No
**Title and abstract**	1	(*a*) Indicate the study's design with a commonly used term in the title or the abstract	1
		(*b*) Provide in the abstract an informative and balanced summary of what was done and what was found	2
**Introduction**			
Background/rationale	2	Explain the scientific background and rationale for the investigation being reported	4
Objectives	3	State specific objectives, including any prespecified hypotheses	5
**Methods**			
Study design	4	Present key elements of study design early in the paper	5
Setting	5	Describe the setting, locations, and relevant dates, including periods of recruitment, exposure, follow-up, and data collection	6
Participants	6	(*a*) Give the eligibility criteria, and the sources and methods of selection of participants	5
Variables	7	Clearly define all outcomes, exposures, predictors, potential confounders, and effect modifiers. Give diagnostic criteria, if applicable	6/7
Data sources/ measurement	8*	For each variable of interest, give sources of data and details of methods of assessment (measurement). Describe comparability of assessment methods if there is more than one group	*6/7*
Bias	9	Describe any efforts to address potential sources of bias	7-8
Study size	10	Explain how the study size was arrived at	
Quantitative variables	11	Explain how quantitative variables were handled in the analyses. If applicable, describe which groupings were chosen and why	5-6
Statistical methods	12	(*a*) Describe all statistical methods, including those used to control for confounding	7-9
		(*b*) Describe any methods used to examine subgroups and interactions	9
		(*c*) Explain how missing data were addressed	6
		(*d*) If applicable, describe analytical methods taking account of sampling strategy	6
		(*e*) Describe any sensitivity analyses	8/12-13
**Results**			
Participants	13*	(a) Report numbers of individuals at each stage of study—eg numbers potentially eligible, examined for eligibility, confirmed eligible, included in the study, completing follow-up, and analysed	5-6
		(b) Give reasons for non-participation at each stage	5-6
		(c) Consider use of a flow diagram	Supplementary S1
Descriptive data	14*	(a) Give characteristics of study participants (eg demographic, clinical, social) and information on exposures and potential confounders	10

For peer review only - http://bmjopen.bmj.com/site/about/guidelines.xhtml

		(b) Indicate number of participants with missing data for each variable of interest	10-12
Outcome data	15*	Report numbers of outcome events or summary measures	6
Main results	16	(*a*) Give unadjusted estimates and, if applicable, confounder-adjusted estimates and their precision (eg, 95% confidence interval). Make clear which confounders were adjusted for and why they were included	13/16
		(*b*) Report category boundaries when continuous variables were categorized	10
		(*c*) If relevant, consider translating estimates of relative risk into absolute risk for a meaningful time period	
Other analyses	17	Report other analyses done—eg analyses of subgroups and interactions, and sensitivity analyses	15-16
**Discussion**			
Key results	18	Summarise key results with reference to study objectives	17
Limitations	19	Discuss limitations of the study, taking into account sources of potential bias or imprecision. Discuss both direction and magnitude of any potential bias	19-20
Interpretation	20	Give a cautious overall interpretation of results considering objectives, limitations, multiplicity of analyses, results from similar studies, and other relevant evidence	17-20
Generalisability	21	Discuss the generalisability (external validity) of the study results	17-20
**Other information**			
Funding	22	Give the source of funding and the role of the funders for the present study and, if applicable, for the original study on which the present article is based	20

*Give information separately for exposed and unexposed groups.

Note: An Explanation and Elaboration article discusses each checklist item and gives methodological background and published examples of transparent reporting. The STROBE checklist is best used in conjunction with this article (freely available on the Web sites of PLoS Medicine at http://www.plosmedicine.org/, Annals of Internal Medicine at http://www.annals.org/, and Epidemiology at http://www.epidem.com/). Information on the STROBE Initiative is available at www.strobe-statement.org.

For peer review only
